# Environmental control programs the emergence of distinct functional ensembles from unconstrained chemical reactions

**DOI:** 10.1073/pnas.1813987116

**Published:** 2019-03-06

**Authors:** Andrew J. Surman, Marc Rodriguez-Garcia, Yousef M. Abul-Haija, Geoffrey J. T. Cooper, Piotr S. Gromski, Rebecca Turk-MacLeod, Margaret Mullin, Cole Mathis, Sara I. Walker, Leroy Cronin

**Affiliations:** ^a^WestCHEM, School of Chemistry, University of Glasgow, Glasgow, United Kingdom G12 8QQ;; ^b^School of Life Sciences, College of Medical, Veterinary and Life Sciences, University of Glasgow, Glasgow, United Kingdom G12 8QQ;; ^c^Beyond Center for Fundamental Concepts in Science, Arizona State University, Tempe, AZ 85287

**Keywords:** origin of life, chemomics, systems chemistry, combinatorial chemistry, peptides

## Abstract

Many approaches to the origin of life focus on how the molecules found in biology might be made in the absence of biological processes, from the simplest plausible starting materials. Another approach could be to view the emergence of the chemistry of biology as process whereby the environment effectively directs “primordial soups” toward structure, function, and genetic systems over time. This does not require the molecules found in biology today to be made initially, and leads to the hypothesis that environment can direct chemical soups toward order, and eventually living systems. Herein, we show how unconstrained condensation reactions can be steered by changes in the reaction environment, such as order of reactant addition, and addition of salts or minerals. Using omics techniques to survey the resulting chemical ensembles we demonstrate there are distinct, significant, and reproducible differences between the product mixtures. Furthermore, we observe that these differences in composition have consequences, manifested in clearly different structural and functional properties. We demonstrate that simple variations in environmental parameters lead to differentiation of distinct chemical ensembles from both amino acid mixtures and a primordial soup model. We show that the synthetic complexity emerging from such unconstrained reactions is not as intractable as often suggested, when viewed through a chemically agnostic lens. An open approach to complexity can generate compositional, structural, and functional diversity from fixed sets of simple starting materials, suggesting that differentiation of chemical ensembles can occur in the wider environment without the need for biological machinery.

## Significance

We show that materials with different structure and function can emerge from the same starting materials under different environmental conditions, such as order of reactant addition or inclusion of minerals. The discoveries we report were made possible by using analytical tools more common in omics/systems biology for functional and structural characterization, retasked for exploring and manipulating complex reaction networks. We not only demonstrate that environments can differentiate fixed sets of starting materials (both mixtures of pure amino acids and the classic Miller–Urey “prebiotic soup” model), but that this has functional consequences. It has been often said that biology is “chemistry with history” and this work shows how this process can start.

Modern synthetic chemistry takes a closed approach to complexity, with a focus on making single molecular targets in high yield, purity, and selectivity. Meanwhile, the exploration of complex mixtures or systems is focused on those formed within, or by, biology (1, 2), since biology imposes boundary conditions on molecular diversity (3) which abiotic chemistry lacks. As researchers interested in how functional ordered chemical systems might be produced from an inorganic world, to ultimately form biological/life-like systems (4, 5), we cannot avoid heterogeneity (3, 6–9). In recent decades, however, most chemists researching life-like systems (10) have moved from exploring high-energy unconstrained primordial soup reactions (11, 12), to examining the intricate mechanisms required for abiotic synthesis of nucleotides (13), polynucleotides (14–16), and peptides (17–20), and on toward the assembly of protocells (12–23), enzyme-mediated systems (24), and exploration of autocatalysis (25, 26). This transition arose from the expectation that unconstrained multicomponent reactions would undergo combinatorial explosion (3). Without some means of control, this would result in analytically intractable, undifferentiated mixtures in which any specific functional molecules would be vanishingly dilute, with no mechanism for the emergence of distinct functional systems or structures (1, 7, 8). However, we feel that this assumption could be challenged by exploring the process of developing chemical complexity over time with the environment directing or “acting” on mixtures of simple molecules.

**Fig. 1.**
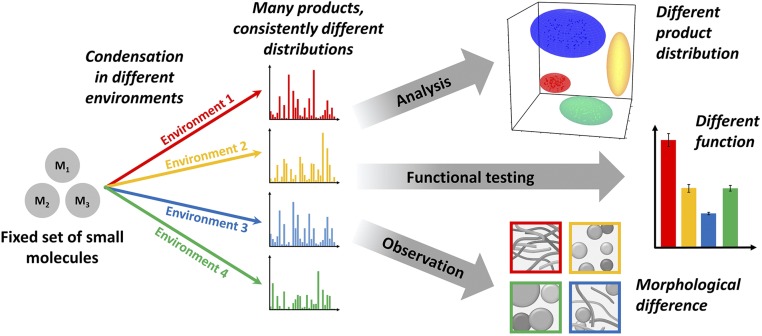
Concept: Uncontrolled condensation reactions make a mess, but they can be steered. Reactions where multifunctional building blocks yield combinatorial explosions may be steered by different environmental conditions to consistently yield different product distributions. These different product ensembles can be shown to have consistently different structural and functional properties.

In recent work, we (9) and others (27, 28) have begun to take a more open approach to complex mixtures; instead of avoiding complexity, we embrace it, and use modern analytical tools to observe otherwise-hidden patterns in complex synthetic systems. Here, we hypothesize that while unconstrained multicomponent reactions do produce a mess, they may be steered to different areas of chemical space. We show that performing a reaction of the same starting materials but under different environmental conditions will consistently yield different chemical ensembles (Fig. 1). These can lead to the emergence of distinct order, structure, and function, “programmed” by the environment, and challenge the view that a complexity-first approach, instead of targeting specific product molecules, will only yield intractable tar (7).

Initially, we chose activation-free dehydration-driven amino acid (AA) condensation as a model system to explore these ideas. Such reactions can be carried out simply by heating aqueous AA solutions to remove water and drive peptide-forming dehydration reactions (12, 18), but can potentially produce diverse ensembles of peptide oligomers with a range of structural and functional properties. In preliminary work, to confirm cross-reactivity between a range of AAs, we noticed that even small changes, like the presence of different soluble salts, could alter the distribution of products, both in the amount of peptide bonds formed and the resulting oligomer distribution. This implies that product distribution is neither random (29), nor completely determined by simple thermodynamic considerations, but rather subject to environmental control. Full experimental details are given in *SI Appendix*, sections S1–S4, including analytical details.

To test our key hypothesis, that environmental programming can produce significantly different chemical ensembles from a fixed set of AAs, we focused on condensation of glycine (G), alanine (A), and histidine (H). All undergo homo- and cross-oligomerization with different degrees of reactivity, and their varied incorporation into peptides might be expected to lead to different functional and structural properties. To assess extended structure formation, alanine (A), aspartic acid (D), and valine (V) were specifically chosen for their potential to form oligomers with hydrophobic and hydrophilic blocks, thus increasing the chance of constructing interesting structural motifs. Earlier studies have found the kind of complex mixtures these reactions produce to be analytically intractable, since robust identification and quantification of the many thousands of potential oligomer products is not feasible (e.g., combination of three AAs in oligomers up to 10 residues long potentially yields 59,049 distinct sequence permutations). Instead of attempting to identify all products, we have developed a chemomics “fingerprinting” approach to observe the resulting chemical ensemble (mixture), mirroring the approach of untargeted metabolomics studies in biological systems. Our workflow starts with liquid chromatography coupled with high-resolution mass spectrometry (LC-MS), which provides a powerful multidimensional means to sensitively resolve large numbers of species (27, 28). Scripted data analysis then allowed automated peak picking to identify features in the LC-MS data (identified by *m/z* and retention time coordinates, and characterized by intensity values for each sample). Finally, dimensionality-reduction approaches to represent data for comparative inspection (principal component analysis), and principal component differential function analysis (PC-DFA), allowed us to extract useful observations from the large volumes of data produced, without any need to assign molecular structures to features (see *SI Appendix*, section 2.2 for full details). This approach follows the example of untargeted metabolomics, rather than proteomics methods, since we also intended to address systems in which products are not restricted to peptides.

## Results and Discussion

We chose three types of environmental condition to vary: (*i*) the presence of soluble salts, (*ii*) the presence of minerals, and (*iii*) the mixing history (the order of precursor addition over multiple reaction cycles). Addition of salts and minerals are both known to interact with AAs in a variety of ways, causing either catalysis, complexation, sequestration, degradation, and/or templating (30–34). Minerals chosen were alumina, montmorillonite, mica, goethite, quartz, natrolite, and silica, while the soluble salts were NaCl, KCl, LiCl, NH_4_Cl, MgCl_2_, CuCl_2,_ and EuCl_3_. A solution containing equimolar amounts of the three AAs was added to these minerals or soluble salts under successive dehydration–hydration cycles (130 °C for 12 h at pH 2.5). Samples were then dialyzed (500–1,000-Da cutoff) to remove small species and soluble salts before analysis. Environmental contributions need not be limited to the material additions, or parameters such as temperature; the history of the material and the order of precursor combination/mixing also have a role (13). To explore this concept, we performed a series of reactions with multiple dehydration/hydration cycles, in which the monomers (G, A, and H) were added to the reaction in different orders with dehydration cycles between each addition.

**Fig. 2.**
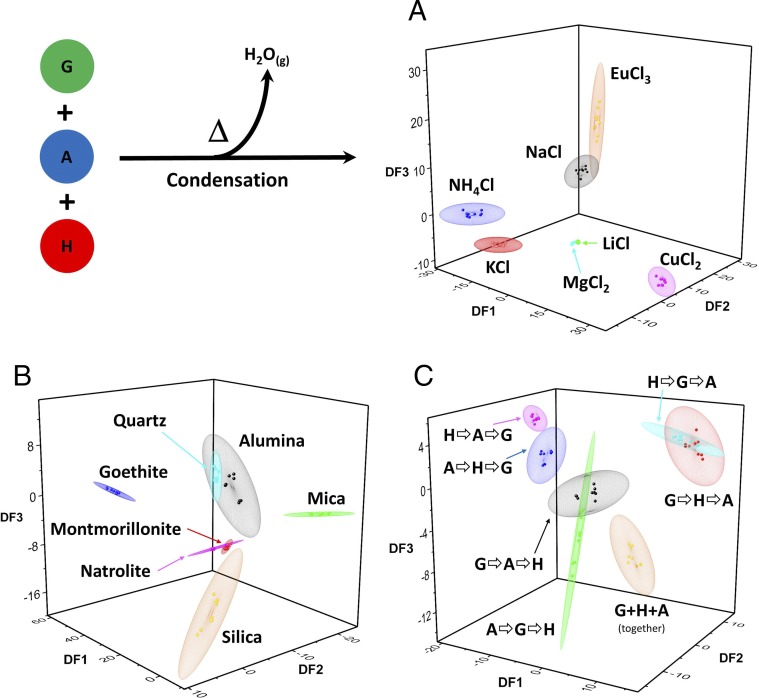
PC-DFA analysis of LC-MS data from condensation of G, A, and H in different environments/conditions: (*A*) different soluble salts, (*B*) different minerals, and (*C*) different mixing orders. Points represent individual measurements (nine measurements: three experimental replicates × three analytical replicates), and shaded bubbles represent a 2-SD space around their mean; see *SI Appendix*, section 2.2 for full details and other representations. Plots generated with Origin Pro-2016 (OriginLab).

Remarkably, we found that all three variations to the environmental conditions led to differentiation of consistently distinct chemical ensembles in terms of peak distribution and intensities from LC-MS analysis (Fig. 2). We used a peak-picking algorithm to define “features” in LC-MS chromatograms, resulting in hundreds to thousands of features for each dataset. Multivariate analysis then allowed us to compare the intensities of these features across the respective environmental parameters, resulting in a 3D interpretation of the uniqueness of chemical compositions between environments. Notably, ensembles resulting from different environments did not overlap, indicating their uniqueness, and individual measurements from different environments cluster together, indicating reproducibility. Furthermore, inspection of LC-MS data by eye (see *SI Appendix*, section 2.2, for full data), in the form of plots of feature intensities, and raw extracted ion chromatograms (EICs), confirms that robust systematic differences can be seen directly in the data.

With the exception of Li^+^, we observed that addition of monovalent soluble salts yielded ensembles with similar compositions. Li^+^ experiments produced compositional distributions similar to those yielded in the presence of Mg^2+^, while presence of Cu^2+^ or Eu^3+^ led to distributions which were clearly distinct from other salts. The reactions incorporating minerals gave ensembles whose compositional distributions were robustly distinguished in all of the analyses performed (with the exception of quartz and alumina). Broadly, the analyses on the experiments with different mixing orders resolve the ensembles yielded into three pairs (G⇨A⇨H & A⇨G⇨H; G⇨H⇨A & H⇨G⇨A; A⇨H⇨G & H⇨A⇨G), with the reaction in which all amino acids were added together clearly resolved from all others. The reaction pattern is consistent with the trends observed in preliminary binary cross-reactivity tests, where G/A heterooligomerization clearly dominates (*SI Appendix*, Fig. S1). For example, products of G⇨A reactions are likely to resemble A⇨G if G/A heterooligomerization rates are very much larger than either possible homooligomerization. While our approach in this work has been nondeterministic, focused on observing differences, these observations hint at the potential for deliberate programming using modeling of reaction rates, although simple models accounting for thermodynamic equilibrium alone are not sufficient.

**Fig. 3.**
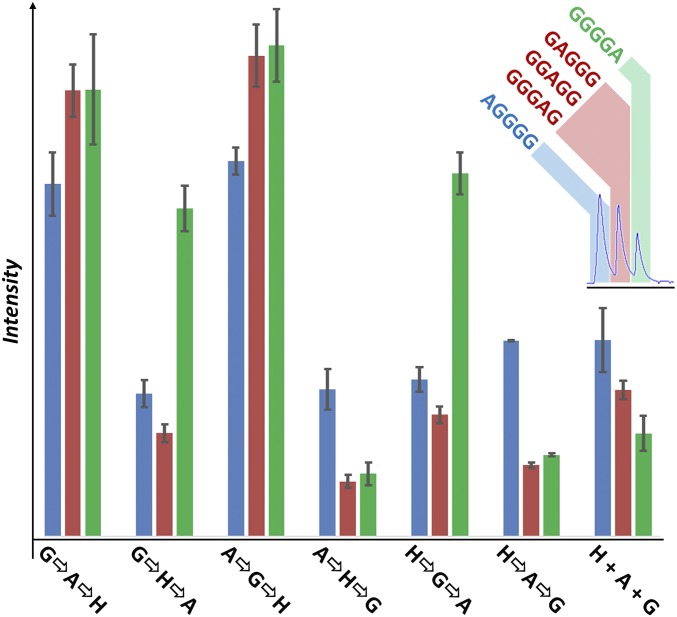
Plots revealing the sequence permutation distribution of G_4_A pentamers. Distribution of mean peak intensity from EICs (*m/z* = 318.141) of samples with different mixing histories, with error bars representing 1 SD. Assignment shown in top right inset. (See *SI Appendix* for peak identification and further details. Peak intensities were extracted using Bruker Data analysis; intensity values displayed are means of three experimental replicates × three analytical repeats.)

The sequence of peptide oligomers is crucial to their function, and while our aim is not to identify individual products, the question of whether product sequence distributions are altered, along with composition and yield, is of interest. While it is possible to match observed masses to be consistent with oligomer compositions (*SI Appendix*, Figs. S23–S25), it is not possible to resolve and quantify all of the myriad product oligomers in most cases, since it requires identifying and separating very similar species, including those of identical mass. In many cases such isomeric species are extremely difficult to resolve using chromatography, especially using a general method, rather than one optimized to resolve specific sequence variants. However, since the shape of the features in the EICs for many masses corresponding to putative oligomers products vary dramatically between populations, it is clear that oligomer sequence, as well as composition, is being steered. The five sequence permutations of G_4_A (*m/z* = 318.141) are a rare example of an oligomer product observed in our ensembles where different sequence permutations can be resolved through chromatography, and variation of their relative abundances observed (Fig. 3). Analysis of synthetic standards of the possible sequence permutations showed that GGGGA and AGGGG could be resolved from GAGGG, GGAGG, and GGGAG, which coelute. Comparison of the mean intensities of these peaks between samples from different mixing histories showed clear variation in the distribution of the sequence permutations (see *SI Appendix*, section 2.2.3 for further details).

**Fig. 4.**
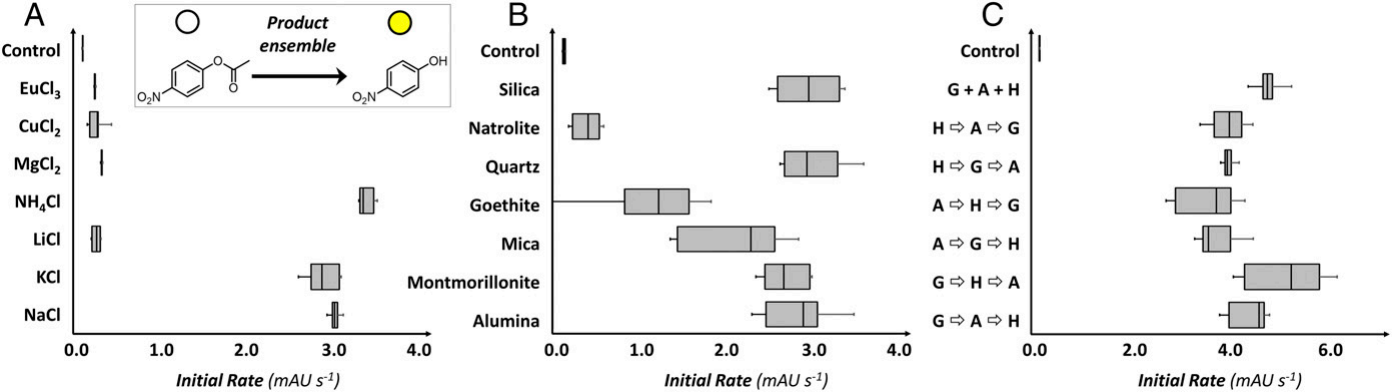
Different product ensembles differentially influence paranitrophenyl acetate conversion. (*Inset*) Decomposition of pNPA to release *p*NP produces a yellow color (measured as absorbance at 405 nm). Box plots comparing rates of *p*NP release on interaction with ensembles produced in different environments/conditions: an equimolar mixture of G, A, and H with (*A*) different soluble salts, and (*B*) different minerals, and (*C*) different mixing orders of G, A, and H over multiple cycles. In all cases the control experiment was with no product present. (Boxes represent middle quartiles, their middle line represents the mean, whiskers represent outlying quartiles.)

Having established that variations to the environment can guide condensation reactions to yield chemical ensembles which are distinct in composition and oligomer sequence, we must then ask if this can also drive functional differences. To assess this, we first observed the effect of the different G/A/H-derived chemical ensembles on the progression of a simple and well-known reaction system, the decomposition of *para*-nitrophenyl acetate (*p*NPA, colorless) to release *para*-nitrophenol (*p*NP, yellow) (35). While absolute rates of the reaction in the presence of our ensembles were much lower than would be expected for catalysis by pure evolved/designed peptide catalysts (35) we found clear and reproducible differences between the effects of many of the environmentally differentiated ensembles. Interestingly, the parameter most significantly affecting differences in esterase activity appears to be soluble salt content; soluble salts have previously been proposed to direct chemistry in dehydrated environments (36). Reproducible differences in the rate of *p*NP release were observed in all of the sets of comparable ensembles, despite *p*NPA being known to interact with a broad range of catalysts (Fig. 4). It is important to note that since the same amount of AA starting materials was used in all condensation reactions, all differences in the effect on the resulting chemical ensembles on *p*NPA reactions are mediated by the environmentally programmed differences between those ensembles.

**Fig. 5.**
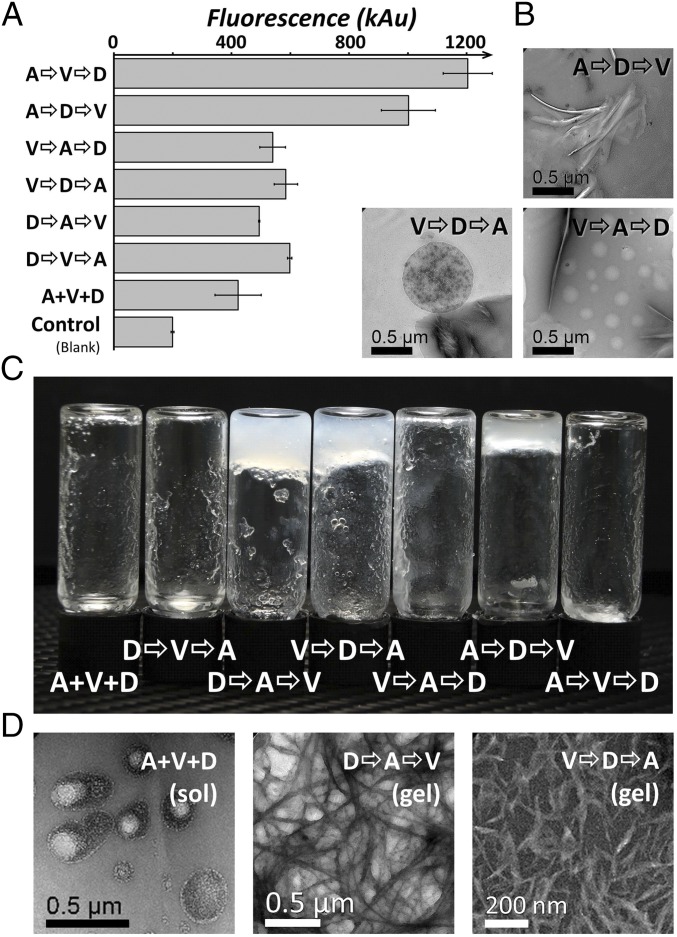
Recognition, assembly, and gelation properties differ between product ensembles. (*A*) ThT assay reveals considerable differences in binding properties of product ensembles from A, V, and D condensation with different mixing orders, and (*B*) TEM images of the same products reveal assembly of qualitatively different structures. (*C*) On addition of Ca^2+^ salts, some ensembles form self-supporting gels, which remain in place when vials are inverted (others leave clear solutions, which flow to the bottom of vials on inversion, *SI Appendix*), and (*D*) TEM inspection of these samples reveals the assembly of fibers in the gelled samples (“gel”), and discrete globular structures in the clear solutions (“sol”).

Molecular recognition is another important class of functionality in macromolecules, so a further set of condensation reactions were performed with different mixing histories, this time with alanine (A), aspartic acid (D), and valine (V). The dye Thioflavin T (ThT) is known to be recognized by hydrophobic sites in peptide assemblies/aggregates, and ThT recognition is frequently used to assess the formation of amyloids, where it is bound with some degree of selectivity (37). Fig. 5*A* shows the fluorescence responses on mixing ThT with the resulting chemical ensembles, and robust differences are observed between some ensembles. This indicates different binding of the dye by these distinct ensembles, themselves yielded by different environments. Based on our previous results with G/A/H oligomerization, this difference in recognition may be sequence dependent. Furthermore, binding of ThT suggests the formation of potentially amyloid-like structures (such assembly is not uncommon in a range of compounds) (38).

We then investigated whether the assembly of nano- or microscale structures in our ensembles (inferred from the ThT experiments) could be observed directly. Transmission electron microscopy (TEM) revealed that different ensembles often assemble into distinct classes of structures (Fig. 5*B* and *SI Appendix*). These range from fiber-like structures to larger globular assembles, some of which appear to incorporate internal structures. Furthermore, addition of Ca^2+^ salts to the A/D/V-derived ensembles leads to gelation in some, while others remain clear, free-flowing, solutions (Fig. 5*C* and *SI Appendix*). TEM imaging of gelled ensembles reveals the assembly of fibrous structures, while that of those in clear solutions reveals discrete assemblies (Fig. 5*D* and *SI Appendix*). It is interesting to note that not all difference/similarity is clearly correlated, their relationship reflecting the complexity of the ensembles. For example, the “V⇨A⇨D” and “V⇨D⇨A” ensembles behave similarly in the ThT interaction measurements (Fig. 5*A*), and yet on addition of Ca^2+^ they behave strikingly differently (one produces a persistent gel-like material, while the other does not; Fig. 5 *C* and *D*). This observation highlights one advantage of an open exploratory approach, over one optimizing only for a specific molecular target or parameter.

Taken together, these results show that the reactivity, assembly, and molecular recognition properties of chemical ensembles yielded by condensation of fixed sets of amino acids can be consistently controlled, or programmed, by their reaction environment, with functional consequences. However, these reactions have only incorporated a small set of pure AA building blocks, which may produce a large but restricted range of products. We then wondered whether the same phenomenon could be observed on applying our approach to a far more complex mixture of starting materials. For this we chose a classic primordial soup: the products of the Miller–Urey type spark discharge experiment (“SD Mix,” hereafter) (11, 39). Starting from a simple mixture of gases (H_2_, CH_4_, NH_3_) and refluxing water, with the gases passing through a high-energy spark discharge between two tungsten electrodes (9), this experiment produces a notoriously complex product mixture. Since SD Mix is known to contain a very wide variety of species, including carboxylic acids, alcohols, aromatic species, and amino acids (11, 39) the possibility of forming condensation products is clear.

**Fig. 6.**
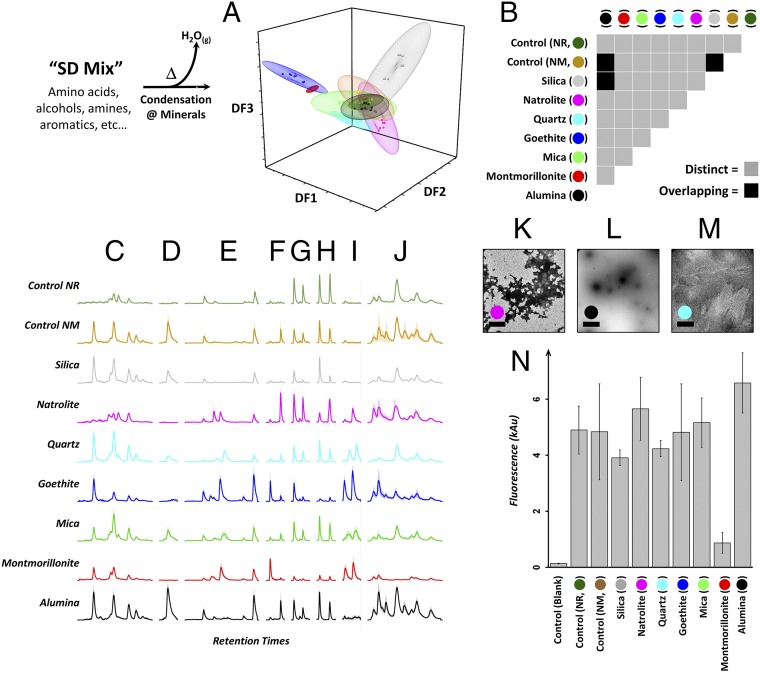
Minerals can direct emergence of distinct ensembles from a primordial soup model. Complex mixtures (SD Mix) were dehydrated in the presence of different minerals. LC-MS data show almost all ensembles are compositionally different: (*A*) LC-MS features as PC-DFA analysis; plot generated with Origin Pro-2016 (OriginLab) (*B*) matrix demonstrating ensemble overlap in PC-DFA [“control (blank),” assay buffer blank; “control NM,” no mineral present; “control NR,” no condensation reaction]. All data are from nine measurements: three experimental replicates × three analytical replicates; Example EICs (*C*–*J*, ordered by ascending *m/z*) selected to demonstrate a range of diversity in the data. Some show almost-complete selectivity between three isobaric species. (*C*, *m/z* = 101.0715; *D*, *m/z* = 102.0918; *E*, *m/z* = 166.0245; *F*, *m/z* = 174.0582; *G*, *m/z* = 244.1907; *H*, *m/z* = 278.0520; *I*, *m/z* = 255.0590; *J*, *m/z* = 321.0014; see *SI Appendix* for further examples.) TEM of ensembles produced in the presence of natrolite (*K*), alumina (*L*), and quartz (*M*) reveals marked morphological differences (Scale bar, 2 μm.) (See *SI Appendix* for full collection of TEM images.) (*N*) Some ensembles interact differently with ThT.

To explore whether condensation reactions of such a complex mixture could be directed by the environment, we performed a series of experiments in which we subjected a standardized SD Mix to similar condensation conditions in the presence of the same series of minerals with which we previously treated AA reactions (see *SI Appendix* for full details). LC-MS fingerprinting of the resulting chemical ensembles (9) (following dialysis to remove salts and small molecules) revealed that reaction in different mineral environments yielded chemical ensembles with a range of compositions which are almost all reproducibly distinct (Fig. 6 *A* and *B*). Closer analysis revealed a wide range of species (up to ∼1,800 features identified; see *SI Appendix*, Fig. S23 for full plot, although some of these may reflect electrospray adducts of the same species with different cations), many of which are not observed in SD Mix controls where no condensation reaction had been carried out. EICs observed in these mineral-programmed ensembles reveal a range of selectivities (see Fig. 6 *C*–*J* for a representative selection, and *SI Appendix* for further examples), with some ensembles manifesting almost complete selectivity for the presence/absence of possible isomeric products. Inspection of EICs and raw data also confirm that while the ensembles are diverse, none is completely lacking in features––their compositions are simply different. While as before, identification of molecular species is not the aim here, tentative assignment of molecular composition for key features can be found in *SI Appendix*, Fig. S41). Furthermore, ThT recognition tests (Fig. 6*N*) revealed that some of these mineral programmed ensembles possess different recognition and assembly properties. For example, ensembles formed in the presence of montmorillonite, silica, and alumina all reproducibly recognize ThT very differently (and all distinct to a control lacking condensation products). TEM morphological examination also shows differences (see Fig. 6 *K*–*M* for representative samples, and *SI Appendix* for additional data). Where some mineral-programmed ensembles give similar results in the ThT recognition assay, they do not necessarily appear to produce morphologically similar assemblies when observed by TEM (e.g., those with goethite and natrolite are clearly distinct). Demonstrating the differentiation of distinct ensembles from what have been seen as classic “intractable” (8) reaction systems adds strength to the increasing argument that “it is possible that the difficulty that chemical heterogeneity presents to early life has been exaggerated” (16) and that this open approach to systems complexity may be more instructive in these contexts than traditional metrics of success in organic chemistry (yield, purity).

## Conclusions

Exploring the mechanism by which complexity and function emerge, and are differentiated, in chemical systems is important for establishing potential origins of evolution (40, 41), pointing to how a variety of ordered systems might emerge from the “clutter wrought by prebiotic chemistry” (42). Indeed, our demonstration of salts and minerals guiding the differentiation of distinct functional ensembles from simple building blocks are an experimental demonstration of Cairns-Smith’s ideas that inorganic materials can program complex organic chemistry to yield differently fit populations (30), beyond simply selecting for particular molecular targets (13, 43). This should be seen as a complement to research identifying particular sets of target molecules (13, 43) which may have been involved in a historical origin of life (44). Our approach to make these observations, using tools from omics sciences with no requirement for target products (9), represents a promising alternative approach to understanding the emergence of complex functional systems where outcomes are tuned by the environment (45). Unlike more familiar approaches (27), it is expandable to address increasingly complex systems, wherein selectivity may be driven by competition and complexity (46), and where approaches based on expectations of particular products are limited (e.g., those relying on databases of known species, or de novo assignment of peptides).

## Materials and Methods

AA condensation experiments in different environments were conducted by first preparing solutions of the relevant AAs (in water, pH adjusted with HCl). To investigate the effect of salts or minerals on the condensation, the AA solutions were added to solutions/suspensions of the salts/minerals and then dehydrated at 130 °C for 12 h. The dry samples were rehydrated with fresh starting solutions and the cycle was repeated (total of 10 cycles were performed). For the investigation of different mixing orders, the experiment was similar but the individual AAs were added in sequence over the cycles. Dried samples were redissolved, dialyzed (500–1,000-Da cutoff) for 20 h, freeze-dried, and then taken up in HPLC-grade water for analysis. Reversed-phase HPLC-MS analyses were performed using a Dionex Ultimate 3000 system fitted with an Agilent Poroshell 120 EC-C18 column and coupled to a Bruker MaXis Impact MS instrument, calibrated for the 50–1,200-Da range. Each reaction (performed in triplicate) was analyzed three times in LC-MS, giving a total of nine repeats (three experimental × three analytical repeats). A qualitative overview of product distribution vs. LC-MS intensity was obtained using bespoke scripts under the R environment, followed by peak picking and more in-depth analysis. Full details of experimental parameters, HPLC-MS methods, and data analysis can be found in the accompanying *SI Appendix*.

## Supplementary Material

Supplementary File
