# Supplementary material for: Environmental control programs the emergence of distinct functional ensembles from unconstrained chemical reactions
Source: Proc Natl Acad Sci U S A. 2019 Mar 6;116(12):5387–92. doi: 10.1073/pnas.1813987116 (PMC6431231; doi:10.1073/pnas.1813987116)
Supplement: Supplementary File [file pnas.1813987116.sapp.pdf]

Environmental control programs the emergence of functional ensembles from unconstrained chemical reaction networks.

Andrew J. Surman,[#a] Marc Rodriguez Garcia,[#a] Yousef M. Abul-Haija,[a] Geoffrey J. T. Cooper,[a] Piotr S. Gromski,[a] Rebecca Turk-MacLeod,[a] Margaret Mullin,[b] Cole Mathis,[c] Sara I. Walker,[c] and Leroy Cronin*[a]

a) *WestCHEM, School of Chemistry, University of Glasgow, Glasgow, UK.*

b) *School of Life Sciences, MVLS, University of Glasgow, Glasgow, UK.*

c) *Beyond Center for Fundamental Concepts in Science, Arizona State University, Tempe, AZ, USA.*

*Corresponding author: Lee.Cronin@glasgow.ac.uk

Table of contents

1 General experimental details 3

1.1 Reagents 3

1.2 General Conditions of RP-HPLC-MS Analysis 3

1.3 Amino Acids 4

1.4 Peptide synthesis 4

2 Environment-Directed Amino Acid (AA) Condensation Experiments 5

2.1 Environment-Directed AA Condensation Experiments: Synthesis 5

2.1.1 Effect of Soluble Salts (G, A, H) 5

2.1.2 Effect of Minerals (G, A, H) 6

2.1.3 Effect of Mixing History (G, A, H) 7

2.1.4 Effect of Mixing History (A, V, D) 8

2.2 Environment-Directed AA Condensation Experiments: Product Analysis 9

2.2.1 Untargeted LC-MS & fingerprinting analysis approach 9

2.2.2 Differing populations: Untargeted LC-MS analysis Results & Discussion 11

2.2.3 Sequence permutation distribution difference between populations 38

www.pnas.org/cgi/doi/10.1073/pnas.1813987116

2.3 Environment-Directed AA Condensation Experiments: Functional Examination.......41
2.3.1 Reactivity testing using *p*NPA.......41
2.3.2 Recognition assay using ThT.......44
2.3.3 Inspection of Assembly/Aggregation using TEM.......45
2.3.4 Observation of different properties of gels produced on addition of Ca^{2+} salts. ...46
3 Environment-Directed Complex Mixture Condensation Experiments.......48
3.1 Spark Discharge Mixture Preparation.......48
3.2 Environment-Directed Complex Mixture Experiments: Synthesis.......49
3.3 Environment-Directed Complex Mixture Experiments: Product Analysis.......50
3.4 Environment-Directed Complex Mixture Experiments: Functional Examination.......57
3.4.1 Recognition assay using ThT.......59
3.4.2 Inspection of Assembly/Aggregation using TEM.......59
4 References.......62

1 General experimental details

1.1 Reagents

All solvents used in synthesis were HPLC grade or higher; all solvents used in LC-MS analyses were LC-MS grade (VWR). Glycine, L-Alanine, L-Aspartic Acid, L-Histidine, L-Valine, *p*-nitrophenyl acetate, sodium chloride, potassium chloride, lithium chloride, magnesium chloride, europium (II) chloride, fumed silica, montmorillonite, Goethite, and Thioflavin T were purchased from Sigma Aldrich. Alumina was purchased from Acros Organics. Copper (II) chloride was purchased from Lancaster/Alfa Aesar. Natrolite and quartz were obtained from Richard Tayler Minerals, Cobham, Surrey, England, and used (crushed in a Teflon ball mill) without further purification. Mica was obtained from Agar Scientific, and used (crushed in a Teflon ball mill) without further purification. "Nanovan" negative stain for TEM was purchased from Nanoprobes. Deuterium oxide was supplied by Goss Scientific. Spectra/Por® Float-A-Lyzer® G2 dialysis tubes were purchased from Spectrum Labs. Gas mixtures were supplied pre-mixed by the British Oxygen Company (BOC) and CK Special Gases Ltd.

1.2 General Conditions of RP-HPLC-MS Analysis

Reversed-phase LC were performed using a Dionex Ultimate 3000 system fitted with an Agilent Poroshell 120 EC-C18 (4.6 x 150 mm, 2.7 μm) column. Samples were typically injected in 2-5 μL aliquots and eluted with a linear gradient mixture of solvents A (water w/0.1% *v/v* formic acid) and B (acetonitrile w/0.1% *v/v* formic acid) over 26 mins as follows: 0 min – 0% B; 4 min – 0% B; 16 min – 70% B; 19 min – 100% B; 23 min – 0% B. The column oven was maintained at 30 °C. The LC system was coupled to a MS apparatus: a Bruker MaXis Impact instrument, calibrated for the 50 – 1200 Da range using sodium formate solution. The eluent stream was introduced directly into the source (no splitting) following the DAD detector, at a dry gas temperature of 200 °C. The ion polarity for all MS scans recorded was positive, with the voltage of the capillary tip set at 4800 V, end plate offset at −500 V, funnel 1 RF at 400 Vpp and funnel 2 RF at 400 Vpp, hexapole RF at 100 Vpp, ion energy 5.0 eV, collision energy at 5 eV, collision cell RF at 200 Vpp, transfer time at 100.0 μs, and the pre-pulse storage time at 1.0 μs. In any MS/MS experiments CID energies were optimised according to products (typically between 20 and 30 eV).

All data acquisition was controlled by the Compass software suite, with DCMS Link/Chromeleon XPress. More complex analyses were performed using bespoke scripts in the R Environment.[1] To facilitate this, data files were converted to the open .mzML format, using Proteowizard MSConvert.[2]

Samples were used directly from the synthesis procedures, as described. Where too much material was present (causing saturation of MS detector through excess signal), all samples in the series were diluted by a 1 in 10 dilution, to allow injection in the 2-5 μl volume range while optimising MS signal. The instrument was calibrated before each set of analytical replicates (each of which was completed before progressing to the next analytical replicate).

1.3 Amino Acids

Where amino acids (AAs) are discussed, they are frequently identified using standard single-letter notation: A = alanine; D = aspartic acid; G = glycine; H = histidine; V = valine. All those incorporating stereocentres are the L- enantiomer.

1.4 Peptide synthesis

Peptide standards (for identification of different G_4A sequence permutations) were synthesised separately using a standard solid phase technique (Fmoc Ala and Gly Wang resin; coupling with DIC/HOBT and a TFA cleavage; Fmoc deprotection with 20% Piperidine/DMF) using a Biotage Initiator+ Alstra Petide Synthesiser. DIC and TFA were purchased from Sigma Aldrich, and protected amino acids and Wang resin were purchased from Activotec.

2 Environment-Directed Amino Acid (AA) Condensation Experiments

2.1 Environment-Directed AA Condensation Experiments: Synthesis

2.1.1 Effect of Soluble Salts (G, A, H)

In this set of experiments, one solution containing an equimolar amount of three different amino acids was reacted to different soluble salts under successive dehydration-hydration cycles.

1. A solution containing three different amino acids (G, A, H) was prepared to a final concentration of 0.033 M (each) and adjusted to pH=2.5 by adding HCl.
2. 7 different soluble salt solutions were prepared at a final concentration of 1 M.

	Experiment Label						
Cycle	NaCl	KCl	LiCl	NH_4Cl	$MgCl_2$	$CuCl_2$	$EuCl_3$
1	G+A+H	G+A+H	G+A+H	G+A+H	G+A+H	G+A+H	G+A+H
2	H_2O	H_2O	H_2O	H_2O	H_2O	H_2O	H_2O
3	H_2O	H_2O	H_2O	H_2O	H_2O	H_2O	H_2O
4	G+A+H	G+A+H	G+A+H	G+A+H	G+A+H	G+A+H	G+A+H
5	H_2O	H_2O	H_2O	H_2O	H_2O	H_2O	H_2O
6	H_2O	H_2O	H_2O	H_2O	H_2O	H_2O	H_2O
7	G+A+H	G+A+H	G+A+H	G+A+H	G+A+H	G+A+H	G+A+H
8	H_2O	H_2O	H_2O	H_2O	H_2O	H_2O	H_2O
9	H_2O	H_2O	H_2O	H_2O	H_2O	H_2O	H_2O

3. 1ml of a 1 M soluble salt solution was added in cycle 1 to each individual experiment.
4. 3.5 ml of the amino acids solutions were added in cycles 1, 4 and 7.
5. 3.5 ml of HPLC water were added in cycles 2, 3, 5, 6, 8 and 9.
6. Each dehydration-hydration cycle was performed on a multiwell hotplate at 130 °C for 12 h (a fixed arbitrary cycle time; all repeat reactions performed together to avoid error).
7. Once finished, all the samples were diluted by adding 6 ml of HPLC water.
8. 500 µl were taken for LC-MS analysis. The remaining sample was dialysed with a G2 Float-a-lyser (500-1000 Da) cut-off (5 ml) for 20 h.
9. Once the dialysis was completed, the samples were left to freeze-dry for 48 h.
10. The solid product material was redissolved in 6 ml of water, filtered through 0.22 µm syringe filters, and stored at 4°C to be used without further treatment.

2.1.2 Effect of Minerals (G, A, H)

In this set of experiments, one solution containing an equimolar amount of three different amino acids was reacted to different minerals under successive dehydration-hydration cycles.

1. A solution containing three different amino acids (G, A, H) was prepared to a final concentration of 0.033 M (each) and adjusted to pH=2.5 by adding HCl.

	Experiment Label						
**Cycle**	**Alumina**	**Montmorillonite**	**Mica**	**Goethite**	**Quartz**	**Natrolite**	**Silica**
1	G+A+H	G+A+H	G+A+H	G+A+H	G+A+H	G+A+H	G+A+H
2	H_2O	H_2O	H_2O	H_2O	H_2O	H_2O	H_2O
3	H_2O	H_2O	H_2O	H_2O	H_2O	H_2O	H_2O
4	G+A+H	G+A+H	G+A+H	G+A+H	G+A+H	G+A+H	G+A+H
5	H_2O	H_2O	H_2O	H_2O	H_2O	H_2O	H_2O
6	H_2O	H_2O	H_2O	H_2O	H_2O	H_2O	H_2O
7	G+A+H	G+A+H	G+A+H	G+A+H	G+A+H	G+A+H	G+A+H
8	H_2O	H_2O	H_2O	H_2O	H_2O	H_2O	H_2O
9	H_2O	H_2O	H_2O	H_2O	H_2O	H_2O	H_2O

2. 0.2 g of a powdered mineral were added in cycle 1 to each individual experiment.
3. 3.5 ml of the amino acids solutions were added in cycles 1, 4 and 7.
4. 3.5 ml of HPLC water were added in cycles 2, 3, 5, 6, 8 and 9.
5. Each dehydration-hydration cycle was performed on a multiwell hotplate at 130 °C for 12 h (a fixed arbitrary cycle time; all repeat reactions performed together to avoid error).
6. Once finished, all the samples were diluted by adding 6 ml of HPLC water.
7. 500 µl were taken for LC-MS analysis. The remaining sample was dialysed with a G2 Float-a-lyser (500-1000 Da) cut-off (5 ml) for 20 h.
8. Once the dialysis was completed, the samples were left to freeze-dry for 48 h.
9. The solid product material was redissolved in 6 ml of water, filtered through 0.22 µm syringe filters, and stored at 4°C to be used without further treatment.

2.1.3 Effect of Mixing History (G, A, H)

In this set of experiments, three different amino acid solutions were added in a different order of addition under successive dehydration-hydration cycles.

1. Three individual solutions of amino acids (glycine, alanine, histidine) were prepared to a final concentration of 0.1 M and adjusted to pH=2.5 by adding HCl. A mixture of the three ("G+A+H") was prepared by mixing these solutions 1:1:1 (*v/v*).
2. The order in which the different amino acid solutions were added was decided.

	Experiment Label						
**Cycle**	**G⇨A⇨H**	**G⇨H⇨A**	**A⇨G⇨H**	**A⇨H⇨G**	**H⇨G⇨A**	**H⇨A⇨G**	**G + A + H**
1	G	G	A	A	H	H	G+A+H
2	H_2O	H_2O	H_2O	H_2O	H_2O	H_2O	H_2O
3	H_2O	H_2O	H_2O	H_2O	H_2O	H_2O	H_2O
4	A	H	G	H	G	A	G+A+H
5	H_2O	H_2O	H_2O	H_2O	H_2O	H_2O	H_2O
6	H_2O	H_2O	H_2O	H_2O	H_2O	H_2O	H_2O
7	H	A	H	G	A	G	G+A+H
8	H_2O	H_2O	H_2O	H_2O	H_2O	H_2O	H_2O
9	H_2O	H_2O	H_2O	H_2O	H_2O	H_2O	H_2O

3. 3.5 ml of a 0.1 M solution of each amino acid were added in cycles 1, 4 and 7.
4. In the experiment where a mixture of the three amino acid solutions was added together, 1.16 ml of each amino acid solution was added.
5. 3.5 ml of HPLC water were added in cycles 2, 3, 5, 6, 8 and 9.
6. Each dehydration-hydration cycle was performed on a multiwell hotplate at 130 °C for 12 h (a fixed arbitrary cycle time; all repeat reactions performed together to avoid error).
7. Once finished, all the samples were diluted by adding 6 ml of HPLC water.
8. 500 µl were taken for LC-MS analysis. The remaining sample was dialysed with a G2 Float-a-lyser (500-1000 Da) cut-off (5 ml) for 20 h.
9. Once the dialysis was completed, the samples were left to freeze-dry for 48 h.
10. The solid product material was redissolved in 6 ml of water, filtered through 0.22 µm syringe filters, and stored at 4°C to be used without further treatment.

    (n.b. A separate set of experiments were also performed in which, instead of dissolving material in a fixed volume of water, a 0.5 mg/ml solution was made up; this is referred to in Section 2.3.1 as 'Constant Concentration' solutions, "CC", rather than 'Constant Volume', "CV").

2.1.4 Effect of Mixing History (A, V, D)

In this set of experiments, three different amino acid solutions were added in a different order of addition under successive dehydration-hydration cycles.

1. Three individual solutions of amino acids (alanine, valine, and aspartic acid) were prepared to a final concentration of 0.1 M. The pH of each solution was acidified to below pH 2.5 by adding 3 ml of 5 M H_3PO_4 followed by adjusting the pH to 2.5 by adding a minimum amount of 5M NaOH.
2. The order in which the different amino acid solutions were added was decided.

	**Experiment Label**						
**Cycle**	**1**	**2**	**3**	**4**	**5**	**6**	**7**
1	A+V+D	D	D	V	V	A	A
2	----	V	A	D	A	D	V
3	----	A	V	A	D	V	D

3. 3.5 ml of a 0.1 M solution of each amino acid were added in cycles 1, 2 and 3.
4. In the experiment where a mixture of the three amino acid solutions was added together, 3.5 ml of each amino acid solution was added.
5. Each single dehydration-hydration cycle was performed on a multiwell hotplate at 130 °C for 12 h (a fixed arbitrary cycle time; all repeat reactions performed together to avoid error).
6. Once finished, all the samples were dissolved in 1.5 ml of HPLC water (by vortex and sonication for 5 min).
7. 500 µl were taken for LC-MS analysis. The remaining sample was pH adjusted to pH=7.0-7.5 with 500 µl of 5 M NaOH then filtered using 0.22 µm nylon syringe filters and stored at 4ºC for further analysis (TEM analysis, ThT assay and gel formation).

2.2 Environment-Directed AA Condensation Experiments: Product Analysis

2.2.1 Untargeted LC-MS & fingerprinting analysis approach

Each reaction (performed in triplicate) was analysed three times in LC-MS, giving a total of 9 repeats (3 experimental x 3 analytical repeats). A qualitative overview of product distribution *vs* LC-MS intensity was obtained using bespoke script, under the R environment,[1] with files input in the ".mzML" format, and the xcms library[3] for data extraction and peak picking functions. The procedure was as follows (results in following sections):

i. Input all data in groups (9 experiments, in 7 groups).

ii. Independently 'pick peaks' (*i.e.* detect features in signal, identified by *m/z* and retention time (*rt*) coordinates and characterised intensity values for each sample).
[xcms 'Centwave' algorithm; 25 ppm error; peak prefilter requiring 7 data points of intensity > 1000; S/N required ≥ 3; scanrange excluding 'column wash' part of LC cycle to minimise 'noise' contributions]

iii. 'Group' peaks/features observed in many experiments with the same *m/z* and *rt*.
[using xcms grouping; bw = 15; mzwid = 0.005]

iv. 'Fill in' missing data. *i.e.* where particular peaks were absent in some samples, extract intensity values at same *rt* and *m/z* values from samples where they were present. *[xcms 'fillPeaks' function]*
This produced a complete table of coordinates (m/z, retention time) for hundreds/thousands of features, along with intensity data for each LC-MS analysis.

v. Perform Principal Component Analysis (PCA) of intensity variation of these picked peaks between analyses. PCA implemented using the FactoMineR library (with scaling),[11] and the first three PCs plotted using the rgl library (version 0.96.0)[12] or Origin Pro 2016,[13] with 'bubbles' plotted around each set of experiments (each environment) representing two standard deviations around their mean (using ellipse3d function from the rgl library).

vi. Principal component discriminant function analysis (PC-DFA) was also performed (using the MASS library),[14] using the first five principal components (these accounted for the overwhelming majority of variance in all cases, see Section 2.2.2). This facilitated sharper observation of the differences between product populations (plotting the first three DFs), but was qualitatively similar to the results of simple (unsupervised) PCA analysis.

Notes and variations on this process:

- No attempt at this stage was made to identify unknown products – the intention of this analysis is to obtain an overview of product distribution, a 'fingerprint', since thorough quantification & identification of every species present is neither practical nor necessary.
- Given these aims, peak picking algorithm settings were deliberately not stringent, to include as many features as possible. We note that while some noise may have been included as a result, its effect is likely to have been negligible: this is demonstrated through the observation that qualitatively similar differentiation of populations is observed when product peaks are filtered to include only potential product peptide masses from the AAs used (see Figure S3) and of the systematic variation of several peaks (see Figures S5 to S7 for example EICs). Furthermore, LC-MS/MS analysis of some species to identify isomers (see Figure S27c for typical example) demonstrates that peptide products are present as expected.
- Isobaric species (those with the same mass) are not resolved in MS detection, and since chromatographic separation frequently did not completely resolve manifolds of isobaric species resulting from different sequence permutations (*e.g.* GGGAG, GGAGG, GAGGG), in many cases it is likely that several species may have been included in the same 'feature' – manifested as broad manifolds of coeluting peaks. Since in many cases the shape (composition distribution) and size (amount of species present) of these features tends to vary in a robust (reproducible) manner, this is not problematic for the conclusions drawn.

2.2.2 Differing populations: Untargeted LC-MS analysis results & discussion

General observations

- In all experiments, analysis reveals that many product populations are clearly and consistently different as a result of the variation of reaction environment: this can be observed in PCA (Figure S3) and PC-DFA analysis (Figure S2), and in extracted ion chromatogram (see Figures S5 to S7 for selected examples, demonstrating reproducible differences) and peak intensity data (Figures S8 to S10).
- PCA yields qualitatively similar results to PC-DFA in demonstrating this, but with less sharp separation. That is, the populations which can be observed to be similar, and those which are clearly resolved, in plots of PCA (Figure S3) are generally those of which similar observations can be made in PC-DFA plots (Figure S2). That PC-DFA, a supervised technique, provides sharper resolution than PCA (an unsupervised technique) is unsurprising; the qualitative similarity reflects the robust and reproducible nature of the difference between populations.
- In all cases, plotting contributions (Figure S4) to the principal components demonstrates that population difference is not defined by a few 'key' features/species; instead, many provide similar (small) contributions.
- Since in most cases experimental repeats produced extremely similar results, in cases where results are not very similar (large 'bubbles') we suspect that this largely due to material loss during sample work-up (filtering; dialysis; filtering; dissolution), for example inconsistency in dialysis membranes. This is consistent with observations during work on these systems (e.g. LC-MS analysis of undialysed samples).
- When the feature list was 'filtered' to exclude all masses not corresponding to a plausible oligomer or the amino acids used (from a combinatorial list of possible peptide products from the AAs combined, as "Peptide mass product distributions", +/- 0.01 Da), the resulting plots (Figure S3) are qualitatively broadly similar to those unbiased by product expectations (the same populations are resolved/unresolved), demonstrating the robustness of the approach and that differences result from 'real' condensation products, not analytical artefacts.

Experiments varying soluble salts present

(Section 2.1.1; MCl_x salts, where M= Na^+, K^+, Li^+, NH_4^+, Mg^{2+}, Cu^{2+}, or Eu^{3+})

- Monovalent salts produced similar product distributions (those from Na^+, K^+, and NH_4^+ unresolved in simple PCA; resolved by PC-DFA, but adjacent), except Li^+.
- Li^+ experiments produced a product distribution similar to that produced in the presence of Mg^{2+}. Presence of Cu^{2+} or Eu^{3+} leads to distributions which are clearly distinct from other salts.

Experiments varying minerals present

(Section 2.1.2; Minerals: Alumina, Montmorillonite, Mica, Goethite, Quartz, Natrolite, Silica)

- Most of the reactions incorporating minerals yielded product ensemble distributions which were robustly distinguished in all analyses performed (supervised and unsupervised), except the alumina/quartz pair.

Experiments varying amino acid mixing history

(Section 2.1.3; Orders: all permutations of sequential addition of G, A, and H; Shorthand "G⇨A⇨H" means G added first, followed by condensation cycles, followed by addition of A, followed by condensation cycles, followed by addition of H, followed by condensation cycles; Shorthand "G+H+A" means all amino acids added together

- Broadly, most of the analyses resolve the ensembles into three pairs (G⇨A⇨H & A⇨G⇨H; G⇨H⇨A & H⇨G⇨A; A⇨H⇨G & H⇨A⇨G), with the reaction in which all amino acids were added together clearly resolved from all. In PC-DFA some of these pairs are resolved (although clearly adjacent), but this separation is not robustly observed across all analyses.
- The reaction pattern is consistent with the trends observed in preliminary binary cross-reactivity tests ("Intensity"=sum of MS intensity accounted-for by putative combinatorial products) where G/A hetero-oligomerisation clearly dominates. For example, products of G⇨A reactions are likely to resemble A⇨G if G/A hetero-oligomerisation rates are very much larger than either possible homo-oligomerisation. While our approach in this work has been non-deterministic, interested in observing difference, these observations point to the potential for deliberate 'programming', using modelling of rate measurements, however, as we observe that simple thermodynamic considerations are not adequate, this will require a more advanced approach.

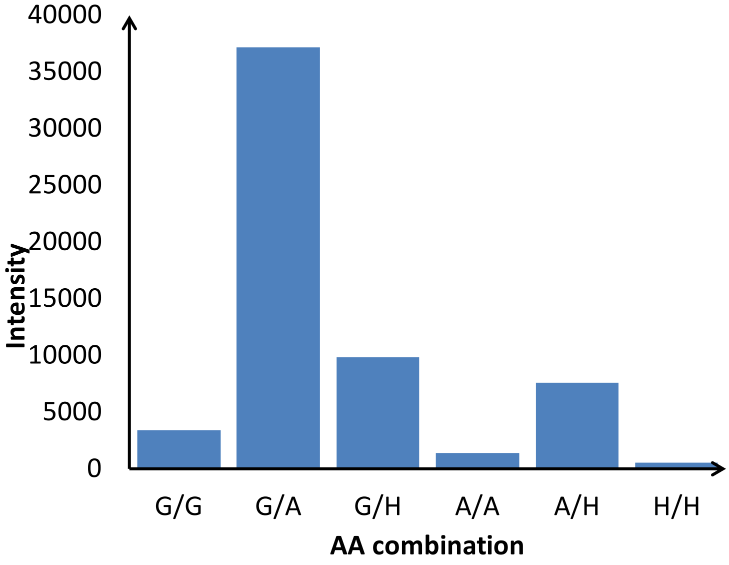

Figure S1. Plot of data from preliminary cross-reactivity investigation for different G, A and H amino acid combinations. “Intensity” is the combined intensity corresponding to the masses of putative oligomeric products (trimer and larger) produced when reacted in simple binary mixtures in the same conditions as used in Section 2.

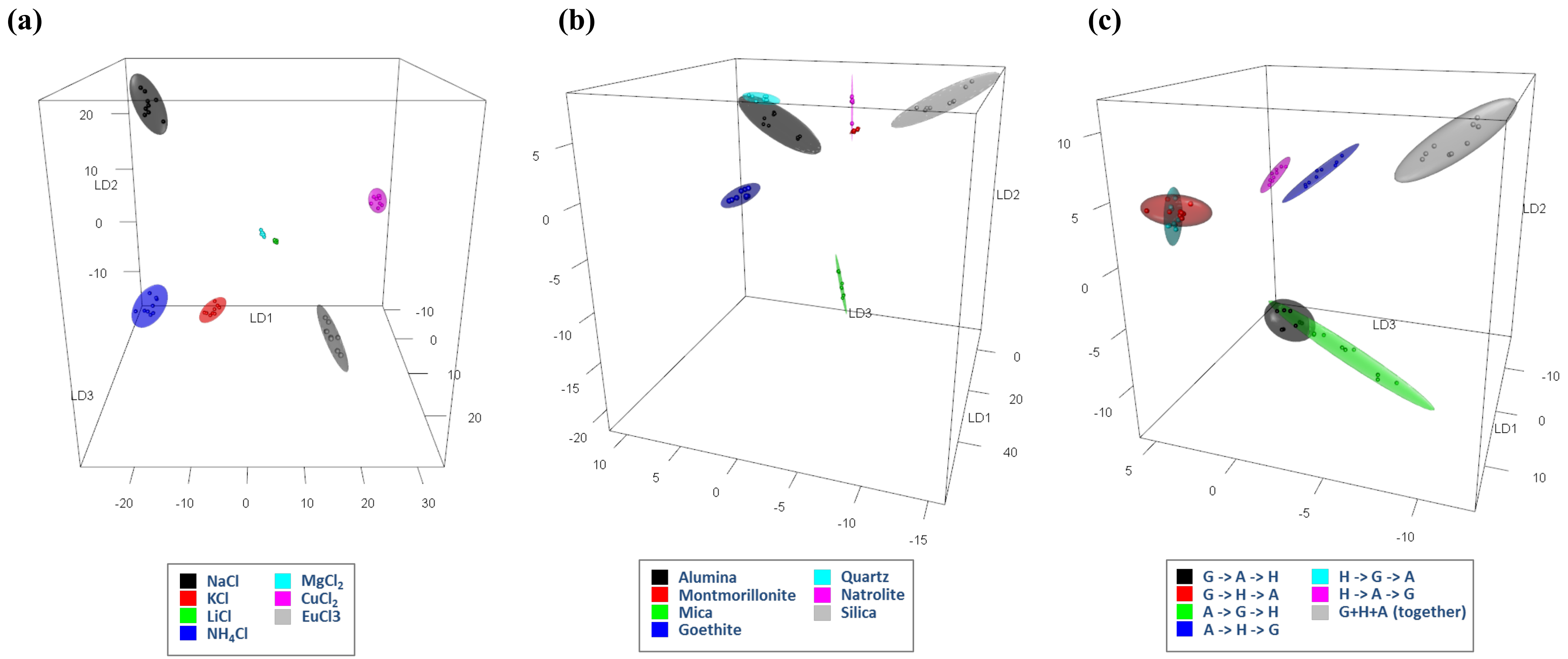

Figure S2. *Plots of PC-DFA analysis (using first 5 PCs) of results from experiments changing (a) soluble salts present, (b) minerals present, and (c) amino acid mixing history; in each case 'spots' represent individual measurements & 'bubbles' represent two standard deviations around their mean. Analysis conducted in R, calculated and plotted using rgl library.*

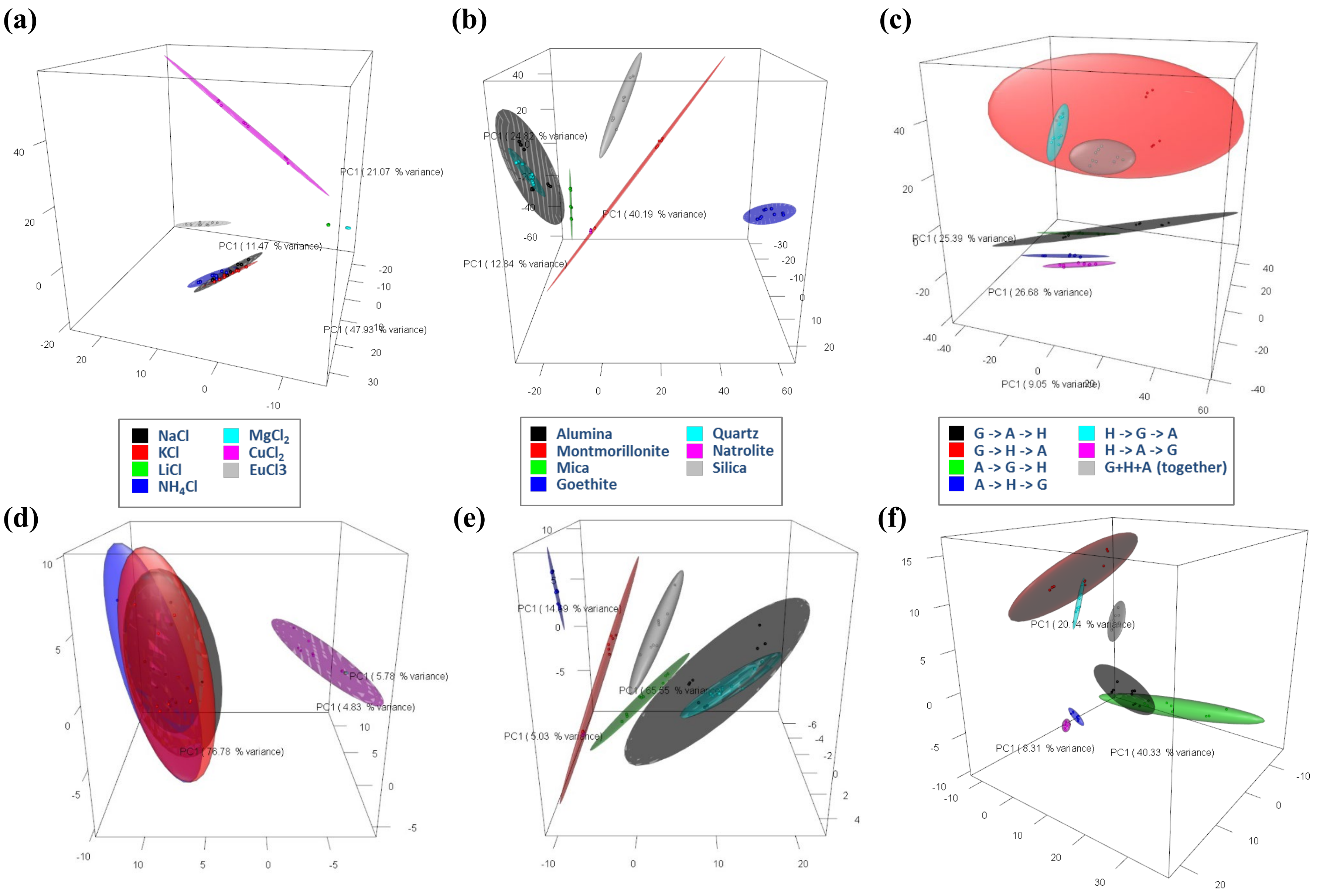

Figure S3. *Plots of PCA analysis comparing the full product distributions (a-c) to 'peptide mass product distributions' (d-f), produced by filtering the feature list for putative peptide products. Data drawn from experiments changing (a & d) soluble salts present, (b & e) minerals present, and (c & f) amino acid mixing history; in each case 'spots' represent individual measurements & 'bubbles' represent two standard deviations around their mean. Analysis conducted in R, calculated and plotted using rgl library.*

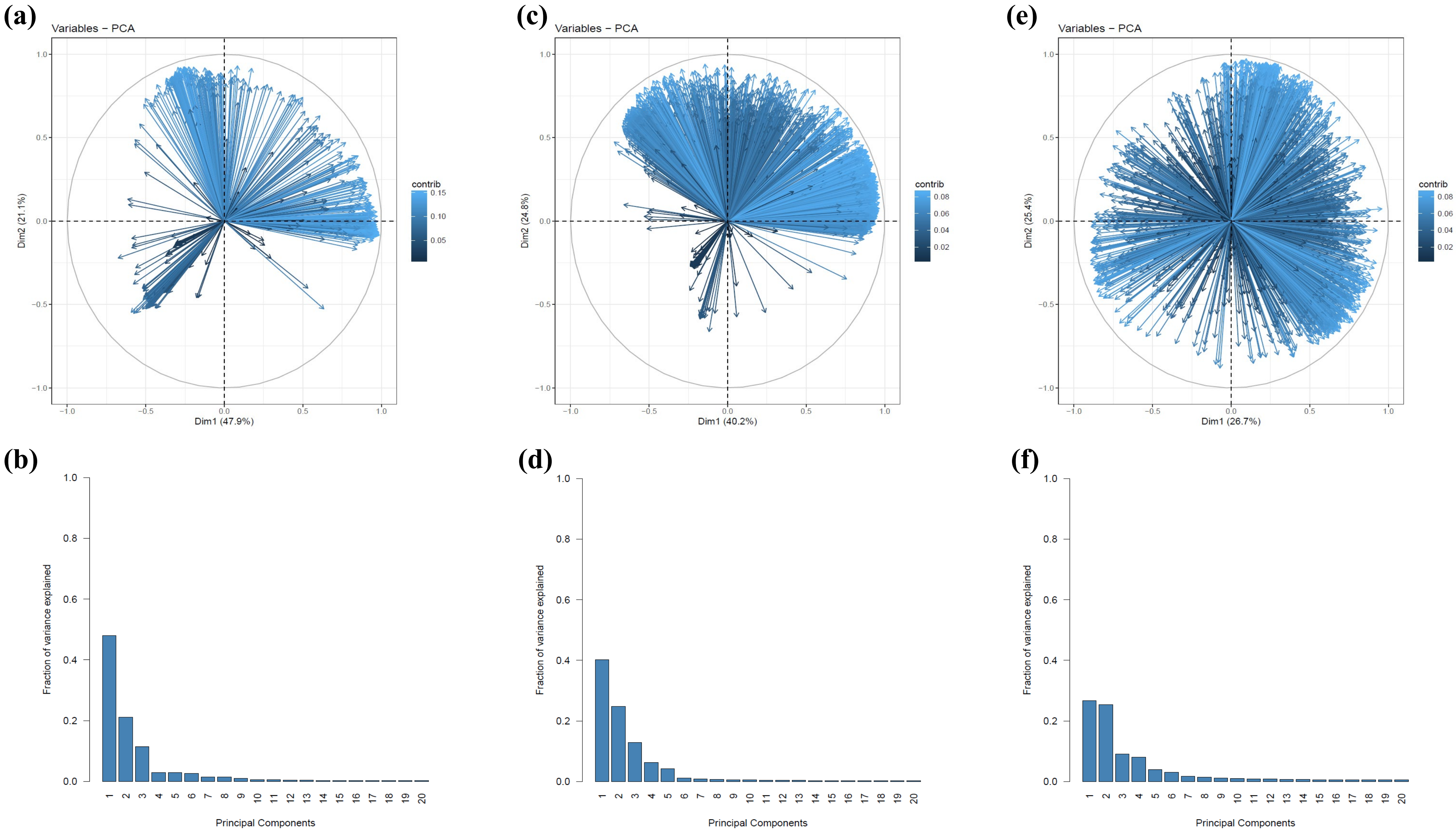

Figure S4. *Plots showing the fraction of variance explained by the principal components in Figure S3 (a-c) and the distribution of contributions to the first two principal components: changing soluble salts present (a & b), changing minerals present (c & d), and amino acid mixing history (e & f).*

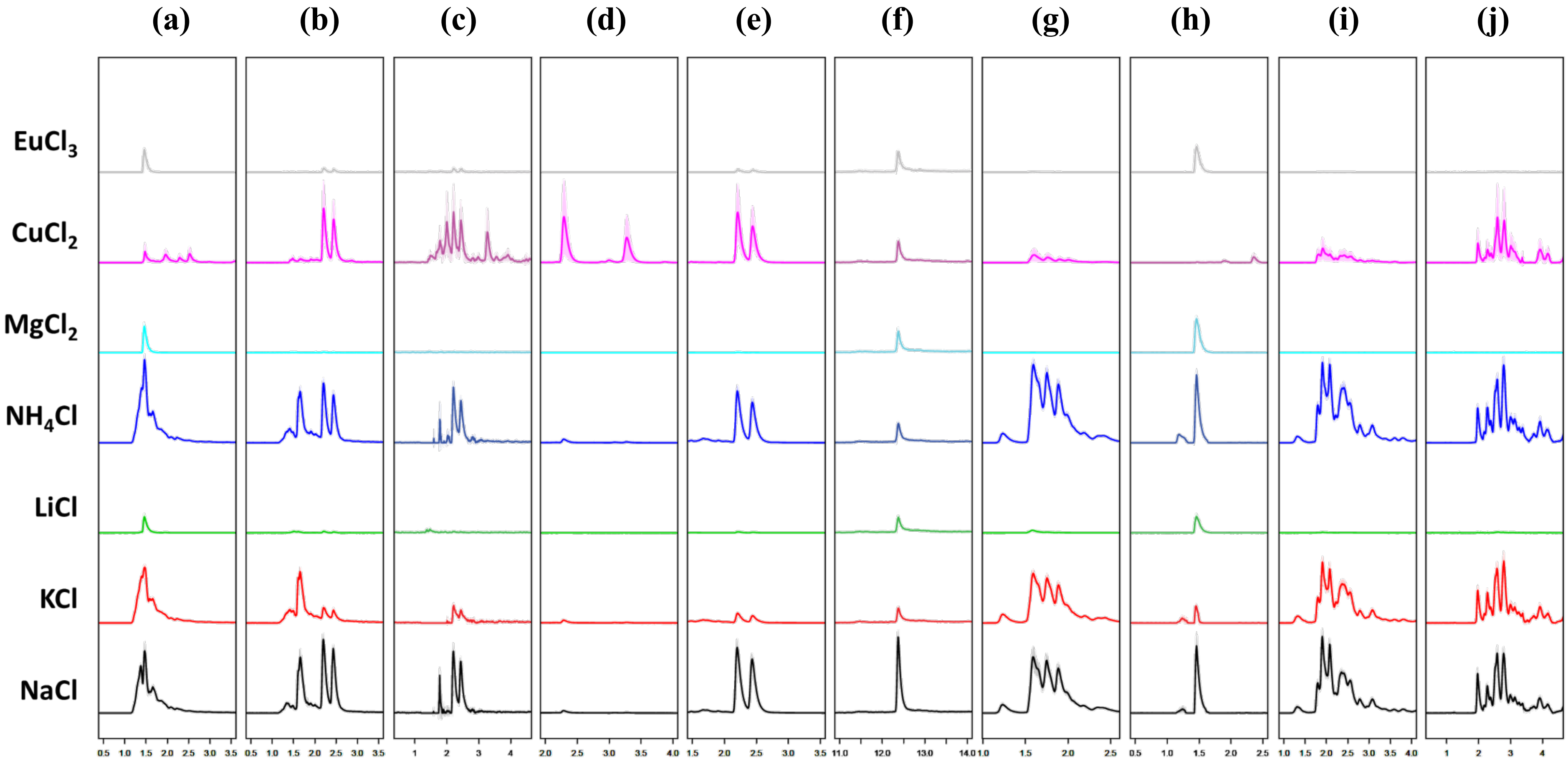

Figure S5. *Selected extracted ion chromatograms illustrating product distribution variance in products from experiments varying soluble salt present (ordered by ascending m/z). (a) m/z = 156.077; (b) m/z = 164.082; (c) m/z = 167.066; (d) m/z = 198.089; (e) m/z = 209.104; (f) m/z = 229.141; (g) m/z = 284.135; (h) m/z = 312.151; (i) m/z = 355.172; (j) m/z = 369.188 [lines = mean intensity from all 9 measurements (3 experimental replicates x 3 analytical replicates); shading around line represents one standard deviation around mean; intensities normalised relative to largest value in each plot]*

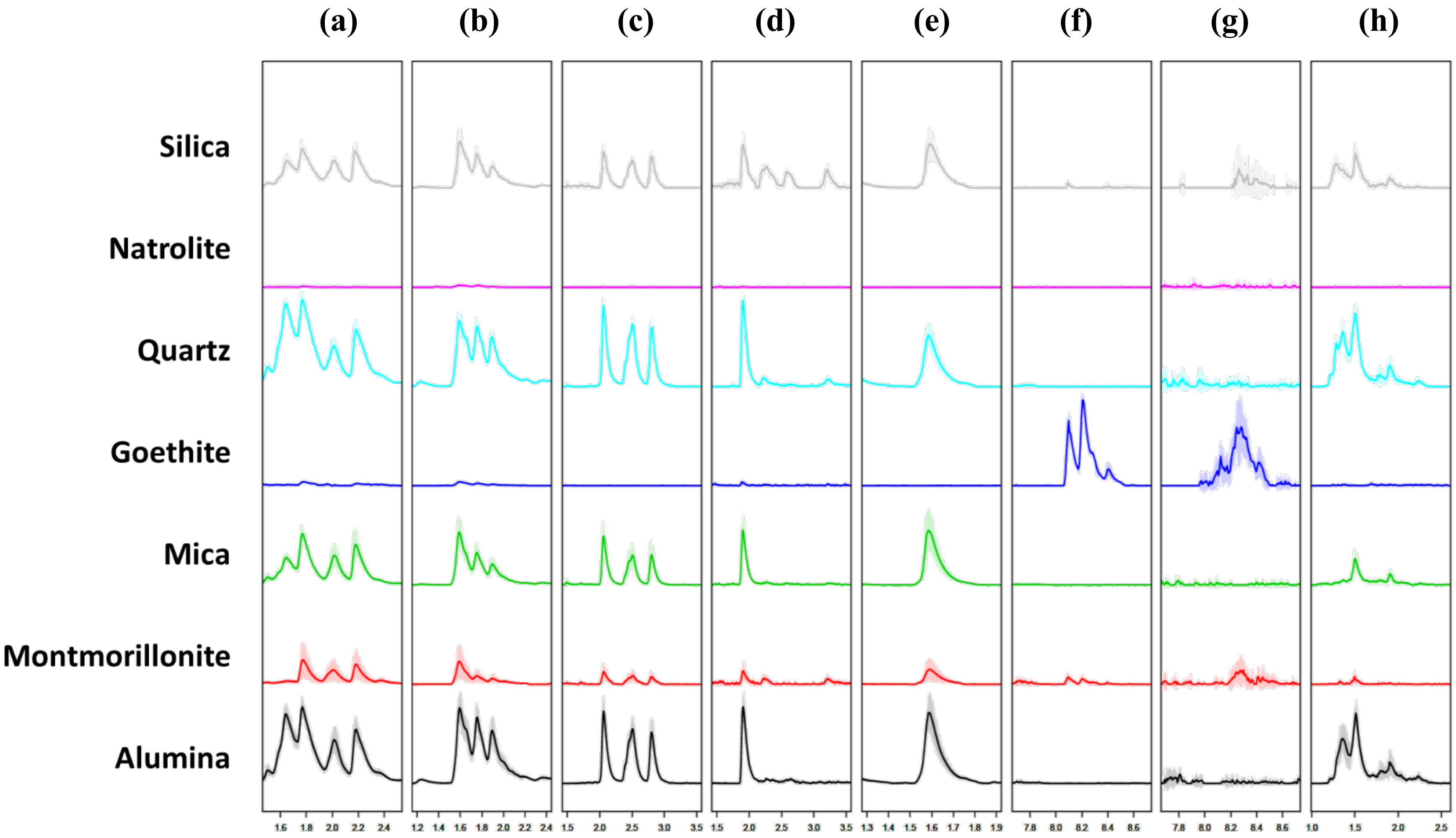

Figure S6. *Selected extracted ion chromatograms illustrating product distribution variance in products from experiments varying which mineral is present (ordered by ascending m/z). (a) m/z = 261.119; (b) m/z = 284.135; (c) m/z = 432.184; (d) m/z = 475.190; (e) m/z = 567.253; (f) m/z = 569.243; (g) m/z = 683.286; (h) m/z = 695.312 [lines = mean intensity from all 9 measurements (3 experimental replicates x 3 analytical replicates); shading around line represents one standard deviation around mean; intensities normalised relative to largest value in each plot]*

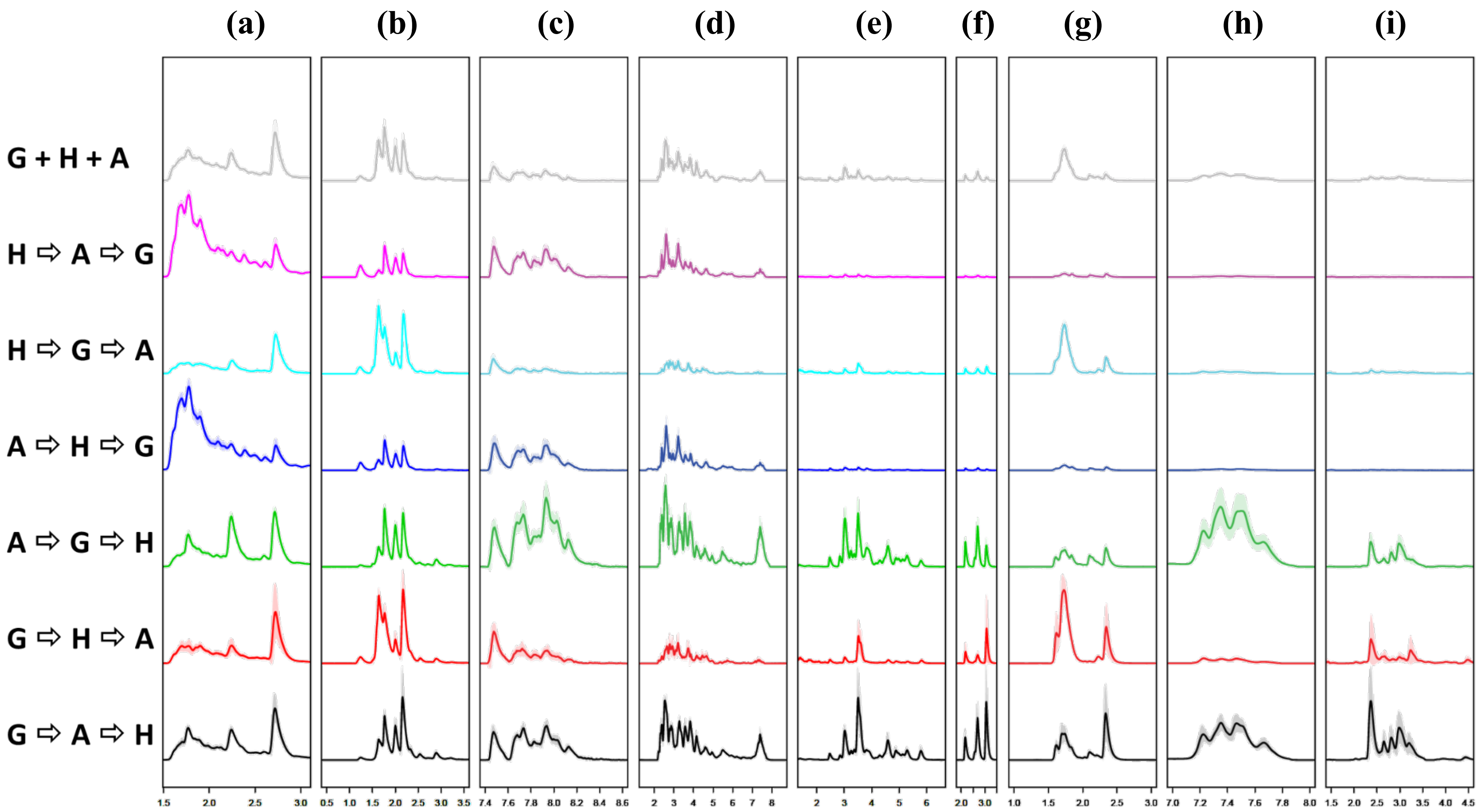

Figure S7. *Selected extracted ion chromatograms illustrating product distribution variance in products from experiments varying amino acid mixing history (ordered by ascending m/z). (a) m/z = 218.114; (b) m/z = 261.119; (c) m/z = 374.203; (d) m/z = 426.210; (e) m/z = 446.119; (f) m/z = 489.205; (g) m/z = 517.237; (h) m/z = 635.264; (i) m/z = 683.286 [lines = mean intensity from all 9 measurements (3 experimental replicates x 3 analytical replicates); shading around line represents one standard deviation around mean; intensities normalised relative to largest value in each plot]*

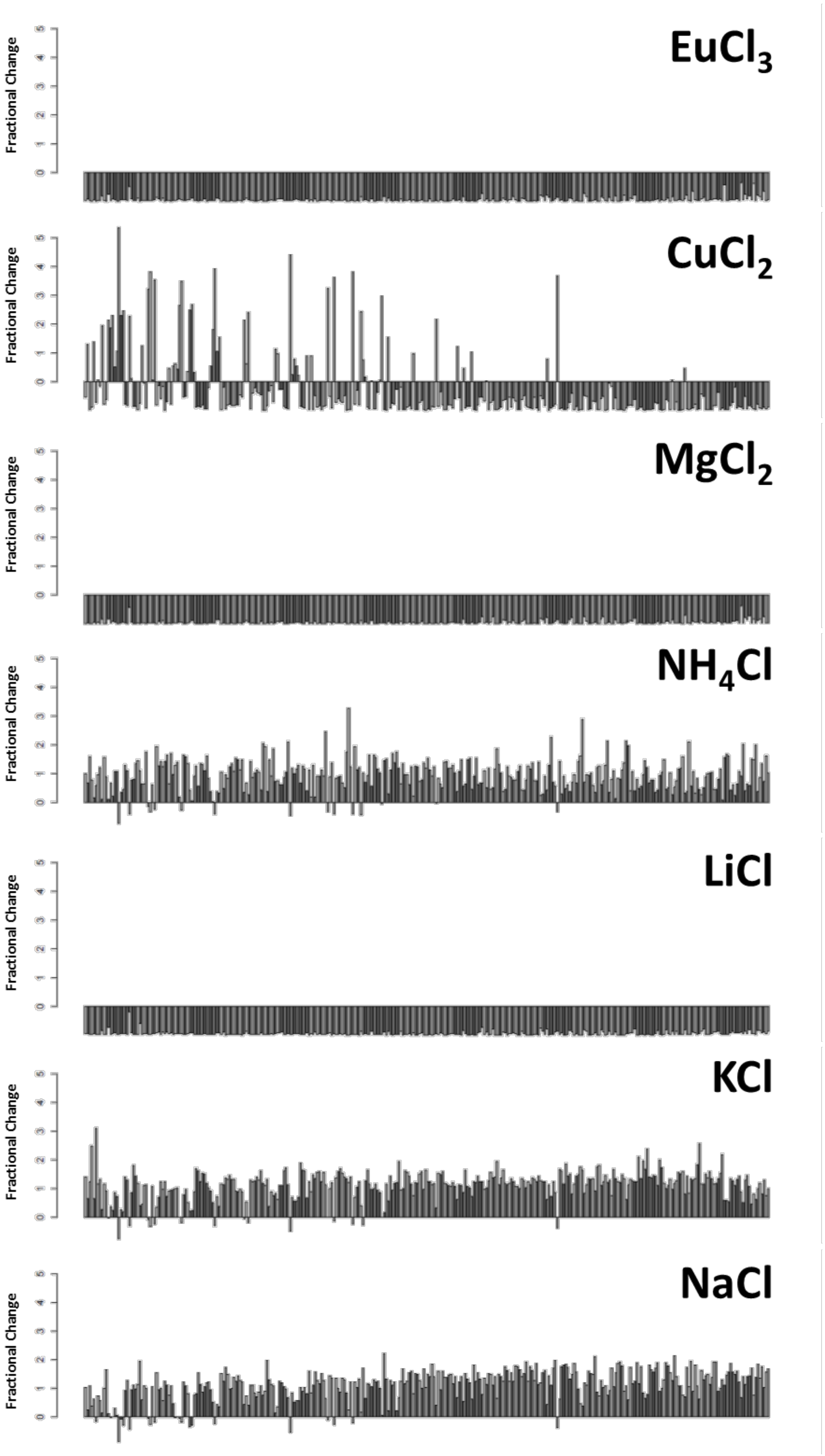

Figure S8. *The intensity of each of the features picked (on which PCA, etc was performed), expressed as a fractional difference from a mean for all the peaks within a set to visualise variation in data.*
[i.e. (Mean^{NaCl} – Mean^{AllSalt}) / Mean^{AllSalt}; feature m/z and rt coordinates unlabelled, ordered by ascending m/z from left to right; Note: Fractional intensities can obscure smaller variations in intensity values below mean]

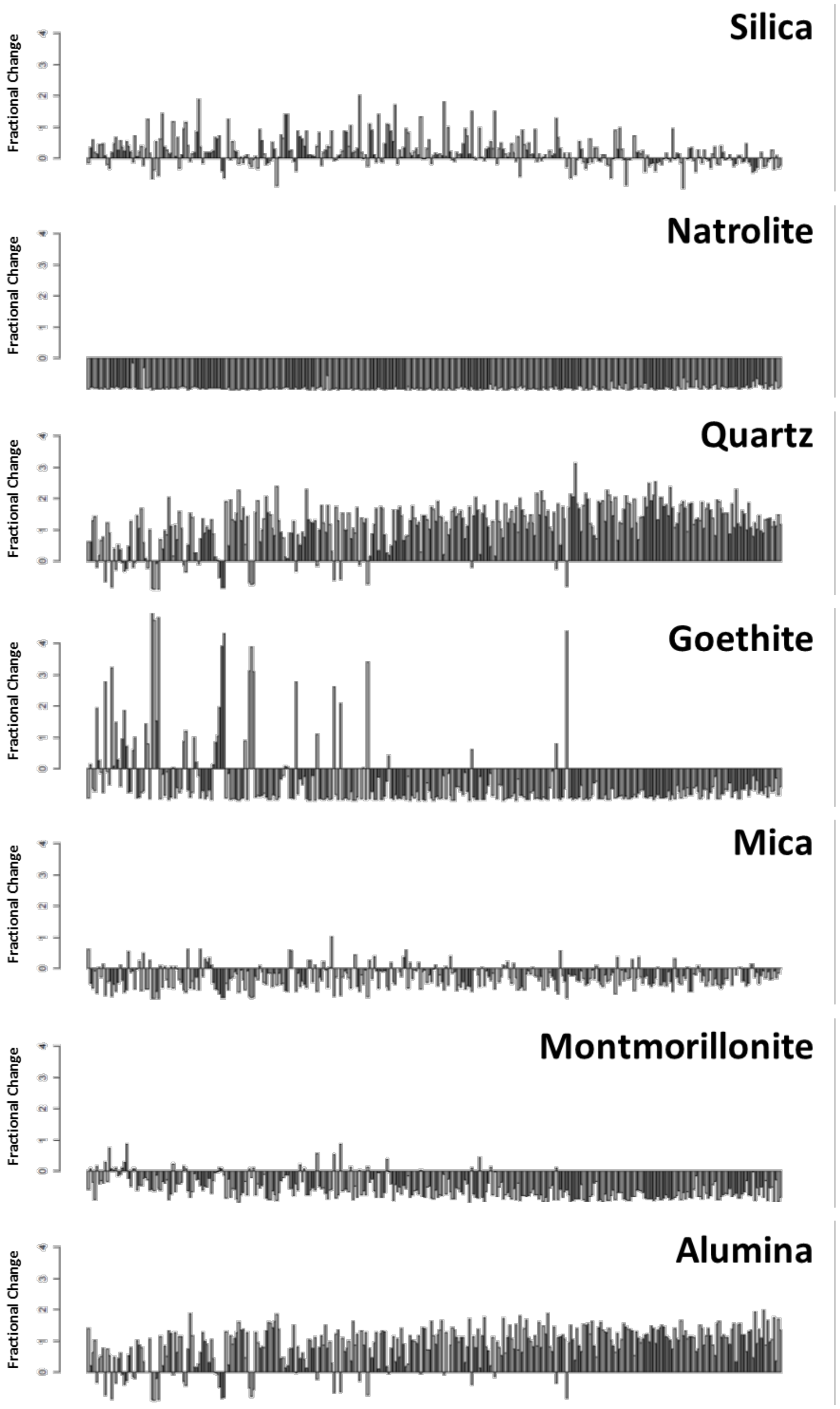

Figure S9. *The intensity of each of the features picked (on which PCA, etc was performed), expressed as a fractional difference from a mean for all the peaks within a set to visualise variation in data.*
[i.e. ($Mean^{Mica}$ – $Mean^{AllMin}$) / $Mean^{AllMin}$; feature m/z and rt coordinates unlabelled, ordered by ascending m/z from left to right; Note: Fractional intensities can obscure smaller variations in intensity values below mean]

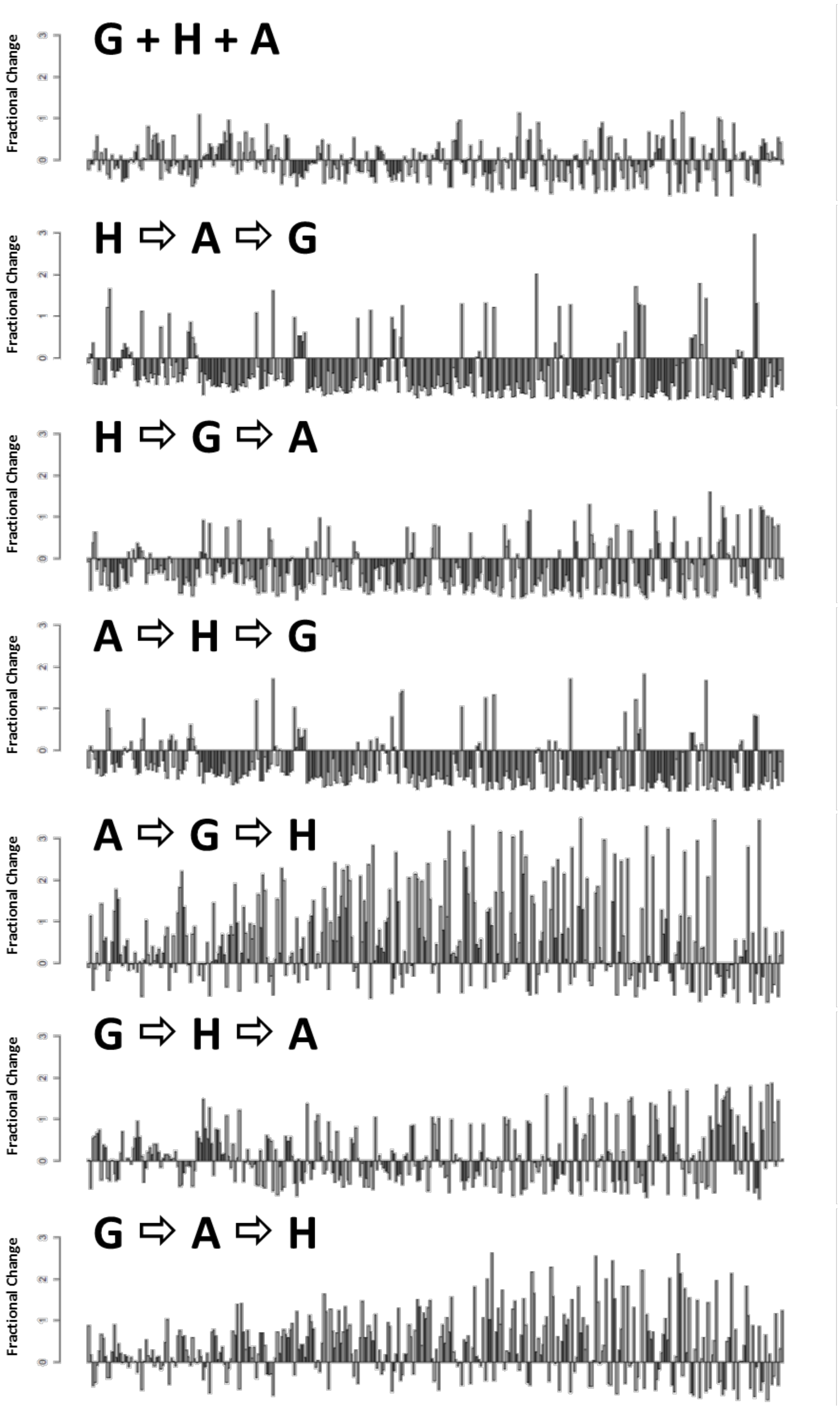

Figure S10. *The intensity of each of the features picked (on which PCA, etc was performed), expressed as a fractional difference from a mean for all the peaks within a set to visualise variation in data.*
[i.e. ($Mean^{G+A+H}$ *–* $Mean^{AllHist}$*) /* $Mean^{AllHist}$*; feature m/z and rt coordinates unlabelled, ordered by ascending m/z from left to right; Note: Fractional intensities can obscure smaller variations in intensity values below mean]*

m/z	RT	NaCl	KCl	LiCl	NH4Cl	MgCl2	CuCl2	EuCl3
95.0614	1.47	81291	111777	27499	139067	49595	26716	39130
110.0721	2.27	438569	337910	3324	412821	1321	259433	5103
110.0723	1.48	4091141	2842384	564957	3759391	954859	289740	795382
111.0753	1.48	249333	203500	32437	440125	53526	16868	44732
121.9674	1.38	4440	3834	419	5065	221	578729	29494
123.9658	1.38	722	351	269	439	247	336082	14816
131.9632	1.40	532	384	619	371	398	221911	8176
143.0819	2.91	18578	28539	1057	31230	114	229529	438
147.0730	1.41	597673	863446	19260	1148462	395	15177	1868
148.9546	1.37	476	492	572	533	313	394289	11515
150.9505	1.40	614	549	460	757	617	179467	5583
152.5805	1.41	372699	353268	4356	474570	82	1144	416
156.0769	2.49	107418	89710	735	85522	481	215217	563
156.0770	1.48	1667356	2654349	346691	3240184	544851	170734	462555
156.0788	1.77	1872180	1998466	159072	2253800	247273	245254	230923
157.0809	1.48	104102	158055	26000	183609	40486	14029	34027
167.0934	1.79	513367	133556	10181	372304	1136	167400	9762
175.5832	1.47	405052	1120653	7383	428945	168	5868	1046
182.9637	1.34	1604	334	457	398	76569	554	325
184.0718	1.97	9422	14020	2547	17693	174	173063	651
184.9620	1.34	877	240	1042	445	23125	1800	382
194.1185	7.80	12629	10981	14451	12310	17354	10645	13083
195.0886	1.80	2783962	491586	37181	2080047	8649	1092272	68293
195.8985	1.40	1355	437	962	383	309	10117	155830
195.9186	1.29	5648	1082	1209	745	812	2699	403399
197.8988	1.41	1231	273	168	208	136	8076	155843
197.9189	1.28	5807	920	460	442	345	2491	452150
200.9744	1.34	309	319	651	252	95054	404	454
201.9737	1.34	282	233	278	267	28106	349	201
202.9715	1.34	275	150	184	216	27424	243	133
204.0953	1.67	181351	466368	1949	114356	235	12478	638
209.1038	2.22	2519913	433848	43118	1778546	15176	1853248	120314
210.1061	2.22	244075	45924	5279	199250	1755	204394	14303
211.1027	1.79	244346	458588	2658	287230	407	17022	960
213.0987	1.60	606675	598152	68881	709448	7065	95742	12671
213.1463	14.53	28464	27997	28197	28487	28556	29122	30237
215.6014	1.33	578071	513889	1648	609688	188	603	511
218.9840	1.34	190	192	147	220	238911	1367	148
219.9865	1.34	646	275	142	481	67241	1092	158
220.9812	1.34	247	247	119	240	71035	319	177
227.0795	2.29	48567	63017	963	44381	90	417951	981
227.1156	1.67	397435	372189	55985	568910	4135	85754	9156
239.6130	1.81	454720	327138	1453	296123	109	7270	469
240.9169	1.28	17374	2312	933	1061	911	829	1505588
242.0015	1.34	174	155	81	166	39965	159	156
242.9182	1.28	19780	2721	1229	1136	960	982	1705296
244.1135	1.34	622346	594080	1654	624014	302	516	457
246.2444	13.85	17274	16973	17413	17866	16832	17457	17355
255.1087	2.54	12348	16189	190	17764	58	380745	233
258.9276	1.28	11363	1664	906	692	778	1095	1087083
260.8539	1.40	3484	682	420	370	465	14541	590439
260.9291	1.28	12554	1793	950	808	915	3225	1187401
270.1215	1.67	442474	310065	15465	421304	92	10403	2811
274.2761	15.33	32374	32178	31739	32512	31605	32463	33021
275.1235	1.50	1494631	799074	32794	2095374	4287	110282	65051
275.1283	1.51	977387	438472	37333	1187790	4418	80934	67382
276.9384	1.28	9044	1397	825	646	673	1559	818361
278.8650	1.40	2962	618	417	426	394	29052	503661
278.9399	1.29	9468	1325	918	658	732	7196	901200
281.9436	1.29	1377	358	592	273	464	2125	177332
283.9453	1.29	1548	320	401	285	357	1386	196250
284.1327	1.91	251775	237533	15810	341876	347	65069	4229
284.1378	1.91	442873	406552	13517	576827	160	47836	1555
293.1381	1.44	309414	310656	10110	462540	1762	5653	1289
294.9490	1.29	4964	577	556	375	586	1426	424602
296.9504	1.29	3215	458	277	301	208	245	321751
301.9558	1.29	1346	293	255	183	326	180	140891
311.1484	1.47	107596	26003	32899	112823	67145	601	48710
333.1184	1.48	8851	5855	2360	4019	29337	280	3963
339.7792	1.40	2112	3462	134	1318	36	241848	2374
341.7777	1.40	345	587	168	321	23	330433	3058
350.1550	1.41	733300	881125	6948	1267554	280	2781	863
350.1600	1.41	822775	917556	8072	1280548	299	3139	915
364.1755	1.50	321495	445925	8016	615381	409	8036	2768
400.9430	1.34	66	55	77	77	29006	63	811
407.1804	1.46	352275	478800	1471	475317	118	1724	547
421.1984	1.58	534376	518929	3345	612085	290	10250	1140
466.7091	1.40	2844	3006	66	1768	16	290545	324
468.7076	1.40	258	314	31	281	51	194447	240

Figure S11. *Table of selected features ordered by m/z from the experiments where the soluble salt present was varied. Features were selected from a full list based on absolute MS intensity (appearing in top 20 for at least one condition); this is an arbitrary reduction of data for more detailed display, and it is important to note that no conclusion should be drawn on the significance of this selection due to the non-linear relationship between abundance and intensity. Intensities are averaged over experimental and analytical replicates.*

m/z	RT	NaCl	KCl	LiCl	NH4Cl	MgCl2	CuCl2	EuCl3
242.9182	**1.28**	19780	2721	1229	1136	960	982	1705296
240.9169	**1.28**	17374	2312	933	1061	911	829	1505588
197.9189	**1.28**	5807	920	460	442	345	2491	452150
260.9291	**1.28**	12554	1793	950	808	915	3225	1187401
258.9276	**1.28**	11363	1664	906	692	778	1095	1087083
276.9384	**1.28**	9044	1397	825	646	673	1559	818361
195.9186	**1.29**	5648	1082	1209	745	812	2699	403399
278.9399	**1.29**	9468	1325	918	658	732	7196	901200
296.9504	**1.29**	3215	458	277	301	208	245	321751
294.9490	**1.29**	4964	577	556	375	586	1426	424602
281.9436	**1.29**	1377	358	592	273	464	2125	177332
301.9558	**1.29**	1346	293	255	183	326	180	140891
283.9453	**1.29**	1548	320	401	285	357	1386	196250
215.6014	**1.33**	578071	513889	1648	609688	188	603	511
200.9744	**1.34**	309	319	651	252	95054	404	454
201.9737	**1.34**	282	233	278	267	28106	349	201
220.9812	**1.34**	247	247	119	240	71035	319	177
202.9715	**1.34**	275	150	184	216	27424	243	133
184.9620	**1.34**	877	240	1042	445	23125	1800	382
218.9840	**1.34**	190	192	147	220	238911	1367	148
244.1135	**1.34**	622346	594080	1654	624014	302	516	457
400.9430	**1.34**	66	55	77	77	29006	63	811
182.9637	**1.34**	1604	334	457	398	76569	554	325
219.9865	**1.34**	646	275	142	481	67241	1092	158
242.0015	**1.34**	174	155	81	166	39965	159	156
148.9546	**1.37**	476	492	572	533	313	394289	11515
121.9674	**1.38**	4440	3834	419	5065	221	578729	29494
123.9658	**1.38**	722	351	269	439	247	336082	14816
131.9632	**1.40**	532	384	619	371	398	221911	8176
260.8539	**1.40**	3484	682	420	370	465	14541	590439
195.8985	**1.40**	1355	437	962	383	309	10117	155830
150.9505	**1.40**	614	549	460	757	617	179467	5583
466.7091	**1.40**	2844	3006	66	1768	16	290545	324
468.7076	**1.40**	258	314	31	281	51	194447	240
339.7792	**1.40**	2112	3462	134	1318	36	241848	2374
278.8650	**1.40**	2962	618	417	426	394	29052	503661
341.7777	**1.40**	345	587	168	321	23	330433	3058
197.8988	**1.41**	1231	273	168	208	136	8076	155843
147.0730	**1.41**	597673	863446	19260	1148462	395	15177	1868
350.1600	**1.41**	822775	917556	8072	1280548	299	3139	915
152.5805	**1.41**	372699	353268	4356	474570	82	1144	416
350.1550	**1.41**	733300	881125	6948	1267554	280	2781	863
293.1381	**1.44**	309414	310656	10110	462540	1762	5653	1289
407.1804	**1.46**	352275	478800	1471	475317	118	1724	547
95.0614	**1.47**	81291	111777	27499	139067	49595	26716	39130
311.1484	**1.47**	107596	26003	32899	112823	67145	601	48710
175.5832	**1.47**	405052	1120653	7383	428945	168	5868	1046
111.0753	**1.48**	249333	203500	32437	440125	53526	16868	44732
110.0723	**1.48**	4091141	2842384	564957	4759391	954859	289740	795382
157.0809	**1.48**	104102	158055	26000	183609	40486	14029	34027
333.1184	**1.48**	8851	5855	2360	4019	29337	280	3963
156.0770	**1.48**	1667356	2654349	346691	3240184	544851	170734	462555
275.1235	**1.50**	1494631	799074	32794	2095374	4287	110282	65051
364.1755	**1.50**	321495	445925	8016	615381	409	8036	2768
275.1283	**1.51**	977387	438472	37333	1187790	4418	80934	67382
421.1984	**1.58**	534376	518929	3345	612085	290	10250	1140
213.0987	**1.60**	606675	598152	68881	709448	7065	95742	12671
204.0953	**1.67**	181351	466368	1949	114356	235	12478	638
227.1156	**1.67**	397435	372189	55985	568910	4135	85754	9156
270.1215	**1.67**	442474	310065	15465	421304	92	10403	2811
156.0788	**1.77**	1872180	1998466	159072	2253800	247273	245254	230923
211.1027	**1.79**	244346	458588	2658	287230	407	17022	960
167.0934	**1.79**	513367	133556	10181	372304	1136	167400	9762
195.0886	**1.80**	2783962	491586	37181	2080047	8649	1092272	68293
239.6130	**1.81**	454720	327138	1453	296123	109	7270	469
284.1327	**1.91**	251775	237533	15810	341876	347	65069	4229
284.1378	**1.91**	442873	406552	13517	576827	160	47836	1555
184.0718	**1.97**	9422	14020	2547	17693	174	173063	651
209.1038	**2.22**	2519913	433848	43118	1778546	15176	1853248	120314
210.1061	**2.22**	244075	45924	5279	199250	1755	204394	14303
110.0721	**2.27**	438569	337910	3324	412821	1321	259433	5103
227.0795	**2.29**	48567	63017	963	44381	90	417951	981
156.0769	**2.49**	107418	89710	735	85522	481	215217	563
255.1087	**2.54**	12348	16189	190	17764	58	380745	233
143.0819	**2.91**	18578	28539	1057	31230	114	229529	438
194.1185	**7.80**	12629	10981	14451	12310	17354	10645	13083
246.2444	**13.85**	17274	16973	17413	17866	16832	17457	17355
213.1463	**14.53**	28464	27997	28197	28487	28556	29122	30237
274.2761	**15.33**	32374	32178	31739	32512	31605	32463	33021

Figure S12. *Table of selected features ordered by RT from the experiments where the soluble salt present was varied. Features were selected from a full list based on absolute MS intensity (appearing in top 20 for at least one condition); this is an arbitrary reduction of data for more detailed display, and it is important to note that no conclusion should be drawn on the significance of this selection due to the non-linear relationship between abundance and intensity. Intensities are averaged over experimental and analytical replicates.*

m/z	RT	NaCl	KCl	LiCl	NH4Cl	MgCl2	CuCl2	EuCl3
**204.0923**	3.02	5181	6166	87	5424	52	8949	54
**204.0953**	1.67	181351	466368	1949	114356	235	12478	638
**218.1094**	1.93	122244	108328	1082	166526	221	12835	803
**218.1134**	2.62	14570	26775	428	29046	33	22173	117
**261.1160**	1.78	60499	86010	655	57240	160	4227	475
**270.1215**	1.67	442474	310065	15465	421304	92	10403	2811
**275.1277**	7.26	9387	8098	365	9174	259	2944	309
**275.1283**	1.51	977387	438472	37333	1187790	4418	80934	67382
**275.1326**	2.66	51860	55589	487	62228	299	10189	325
**284.1327**	1.91	251775	237533	15810	341876	347	65069	4229
**284.1378**	1.91	442873	406552	13517	576827	160	47836	1555
**298.1533**	2.25	44642	38966	486	66118	50	11997	443
**327.1430**	1.78	159347	142156	977	188869	96	5184	574
**332.1482**	1.36	133398	166117	944	152817	217	1056	259
**341.1523**	1.33	118881	142657	548	105014	152	7620	100
**341.1565**	2.17	160772	141577	779	160775	162	13724	395
**341.1601**	2.18	187583	154770	766	224560	160	39037	395
**350.1550**	1.41	733300	881125	6948	1267554	280	2781	863
**350.1600**	1.41	822775	917556	8072	1280548	299	3139	915
**355.1677**	1.59	31698	39450	166	14645	119	1720	103
**355.1702**	1.99	112959	74790	1216	76827	114	30833	638
**355.1757**	1.92	149316	124614	1290	166874	117	26526	675
**364.1755**	1.50	321495	445925	8016	615381	409	8036	2768
**369.1904**	2.78	14769	14560	182	18881	83	10212	184
**384.1650**	1.64	223278	199601	722	234681	68	2175	790
**398.1741**	1.87	112325	103148	554	193086	118	4973	346
**398.1789**	1.89	194539	179934	1036	199310	225	11920	824
**407.1804**	1.46	352275	478800	1471	475317	118	1724	547
**417.2034**	2.43	14933	2607	67	11394	35	9800	158
**421.1984**	1.58	534376	518929	3345	612085	290	10250	1140
**430.1945**	1.37	136750	114699	441	147853	192	149	117
**430.1989**	1.30	135843	127486	409	147897	178	124	116
**435.2125**	1.70	103067	105607	650	110105	63	9635	482
**487.2180**	1.33	272083	237889	345	278866	96	160	278
**567.2564**	1.24	12303	9566	38	15493	36	26	598
**638.2966**	1.26	11334	15619	48	14555	66	31	1314

Figure S13. *Table of selected features ordered by m/z from the experiments where the soluble salt present was varied. Features were selected from a full list based on absolute MS intensity, filtered to include masses consistent with peptides, (appearing in top 20 for at least one condition); this is an arbitrary reduction of data for more detailed display, and it is important to note that no conclusion should be drawn on the significance of this selection due to the non-linear relationship between abundance and intensity. Intensities are averaged over experimental and analytical replicates.*

m/z	RT	NaCl	KCl	LiCl	NH4Cl	MgCl2	CuCl2	EuCl3
567.2564	**1.24**	12303	9566	38	15493	36	26	598
638.2966	**1.26**	11334	15619	48	14555	66	31	1314
430.1989	**1.30**	135843	127486	409	147897	178	124	116
341.1523	**1.33**	118881	142657	548	105014	152	7620	100
487.2180	**1.33**	272083	237889	345	278866	96	160	278
332.1482	**1.36**	133398	166117	944	152817	217	1056	259
430.1945	**1.37**	136750	114699	441	147853	192	149	117
350.1600	**1.41**	822775	917556	8072	1280548	299	3139	915
350.1550	**1.41**	733300	881125	6948	1267554	280	2781	863
407.1804	**1.46**	352275	478800	1471	475317	118	1724	547
364.1755	**1.50**	321495	445925	8016	615381	409	8036	2768
275.1283	**1.51**	977387	438472	37333	1187790	4418	80934	67382
421.1984	**1.58**	534376	518929	3345	612085	290	10250	1140
355.1677	**1.59**	31698	39450	166	14645	119	1720	103
384.1650	**1.64**	223278	199601	722	234681	68	2175	790
204.0953	**1.67**	181351	466368	1949	114356	235	12478	638
270.1215	**1.67**	442474	310065	15465	421304	92	10403	2811
435.2125	**1.70**	103067	105607	650	110105	63	9635	482
327.1430	**1.78**	159347	142156	977	188869	96	5184	574
261.1160	**1.78**	60499	86010	655	57240	160	4227	475
398.1741	**1.87**	112325	103148	554	193086	118	4973	346
398.1789	**1.89**	194539	179934	1036	199310	225	11920	824
284.1327	**1.91**	251775	237533	15810	341876	347	65069	4229
284.1378	**1.91**	442873	406552	13517	576827	160	47835	1555
355.1757	**1.92**	149316	124614	1290	166874	117	26526	675
218.1094	**1.93**	122244	108328	1082	166526	221	12835	803
355.1702	**1.99**	112959	74790	1216	76827	114	30833	638
341.1565	**2.17**	160772	141577	779	160775	162	13724	395
341.1601	**2.18**	187583	154770	766	224560	160	39037	395
298.1533	**2.25**	44642	38966	486	66118	50	11997	443
417.2034	**2.43**	14933	2607	67	11394	35	9800	158
218.1134	**2.62**	14570	26775	428	29046	33	22173	117
275.1326	**2.66**	51860	55589	487	62228	299	10189	325
369.1904	**2.78**	14769	14560	182	18881	83	10212	184
204.0923	**3.02**	5181	6166	87	5424	52	8949	54
275.1277	**7.26**	9387	8098	365	9174	259	2944	309

Figure S14. *Table of selected features ordered by RT from the experiments where the soluble salt present was varied. Features were selected from a full list based on absolute MS intensity, filtered to include masses consistent with peptides, (appearing in top 20 for at least one condition); this is an arbitrary reduction of data for more detailed display, and it is important to note that no conclusion should be drawn on the significance of this selection due to the non-linear relationship between abundance and intensity. Intensities are averaged over experimental and analytical replicates.*

m/z	RT	Alumina	Montmoril.	Mica	Goethite	Quartz	Natrolite	Silica
**95.0607**	1.47	145157	289066	138974	342833	146885	38298	234199
**110.0713**	7.41	364164	351148	84117	568845	435159	11725	958289
**110.0715**	1.81	1035866	687981	446650	1142986	2783821	131242	1759590
**111.0742**	1.48	225188	77487	150542	79025	241627	39939	165199
**147.0716**	1.40	740358	51620	398704	10448	749182	9076	350371
**152.5797**	1.40	357541	16051	134391	3551	341267	6916	128251
**154.9073**	1.33	337	441	6695	210	12237	140056	2254
**156.0764**	7.40	185999	99617	67575	121150	248873	12003	664988
**156.0765**	1.59	2024089	724691	1831942	1023374	2309911	419797	1880253
**156.0767**	3.77	21543	62783	7596	574864	22279	1512	69405
**157.0793**	1.65	155643	64398	114414	66553	150392	29600	122255
**167.0925**	1.64	173668	138856	220184	59014	198455	7332	91836
**168.0768**	1.44	19697	345645	31007	453617	19130	24846	117762
**175.0974**	1.63	81035	247721	242281	48134	42781	20891	244586
**175.5824**	1.48	546595	27370	201717	5460	404957	7829	213271
**179.9021**	1.33	222	349	3436	1159	6429	76960	1260
**180.0763**	5.35	3083	26431	941	343724	2102	504	19676
**180.0764**	6.38	4334	44252	1767	424188	2994	463	47418
**180.0765**	2.95	10720	71250	3826	660407	7113	1453	38173
**180.9015**	1.33	255	430	3994	2566	7377	91702	1405
**181.1078**	2.08	300721	152646	225367	36155	175437	2490	449602
**181.9025**	1.33	562	887	6174	2370	11727	137192	2148
**182.0917**	1.49	13042	169030	21222	201906	24374	17222	27646
**182.9022**	1.33	1405	2779	40968	1180	77493	938129	13335
**184.0717**	1.98	32902	214129	21188	352553	33487	30513	119416
**195.0876**	1.80	3560203	2007331	2897008	390669	2019202	34010	5572833
**196.0901**	1.80	287575	163615	231349	31968	163565	3560	457434
**198.0868**	3.30	6111	115601	4644	141804	4203	612	56031
**209.0669**	2.29	1291658	694515	1016808	119483	689164	11990	878018
**209.1030**	2.22	3893142	2101418	2971439	380200	1969699	28795	6189056
**210.1054**	2.23	348576	181170	275068	40840	195902	3370	552761
**211.0686**	4.66	1599	26368	941	342118	1604	409	9850
**211.1010**	1.72	219607	14722	77880	18768	339656	4001	102547
**213.0977**	1.78	311076	85252	166383	58108	121052	113360	351759
**215.6010**	1.33	304632	5532	92751	1542	336540	2333	138847
**227.0769**	2.30	29228	49306	16908	200786	15244	1583	248325
**227.1133**	1.67	379633	87850	175657	53876	358354	25397	183543
**239.6115**	1.77	338982	11252	101541	2438	284942	1909	139587
**241.0928**	2.05	21817	132535	18589	174107	27104	4429	90458
**244.1118**	1.34	381586	6170	104192	3535	410070	1199	136826
**246.2419**	13.84	34852	34129	33625	33828	33949	34868	32923
**246.8617**	1.36	399	872	419	432553	2090	352	244
**248.1140**	1.66	90834	509572	519967	287227	72684	14117	1276975
**264.8731**	1.36	424	667	372	375514	1603	155	176
**267.1082**	1.53	2662	1649	1881	1145	3661	97539	2443
**269.1236**	6.38	4850	45283	2195	535933	3111	607	60119
**269.1241**	3.76	9435	76840	2128	1098840	5416	726	39584
**269.1241**	5.35	5368	36910	1708	497269	4103	507	32830
**270.1189**	1.71	476984	57820	251635	12659	584752	30845	208252
**274.2736**	15.32	73172	73482	73860	75945	74789	75705	73976
**275.1253**	1.50	1680287	320607	848873	36275	1332334	13956	1264051
**282.8828**	1.35	438	589	394	370686	1403	148	196
**284.1340**	1.90	336839	39968	157867	13967	320152	8342	173959
**296.1212**	8.21	1314	17654	686	380718	1428	388	19261
**311.1458**	1.48	179388	157869	195744	153345	114290	46401	253705
**350.1566**	1.41	784568	33536	260495	4827	769266	5518	260848
**407.1786**	1.48	434421	15572	150927	1370	319420	1372	187852
**410.8026**	1.33	172	191	137	4143	325	72328	190
**421.1932**	1.56	382467	27989	153837	2216	449483	2512	149212
**478.2160**	1.64	268450	14308	100801	671	291750	809	105140
**505.8861**	1.14	216	83	613655	6882	636	121	218
**514.8905**	1.15	771	176	2671729	32395	2305	356	714
**515.8902**	1.14	185	78	310071	4921	393	97	157
**523.8958**	1.14	189	53	623647	6700	498	89	206
**528.8886**	1.14	162	91	334769	4334	382	61	152
**532.9017**	1.14	280	89	578900	8100	669	104	231

Figure S15. *Table of selected features ordered by m/z from the experiments where the mineral present was varied. Features were selected from a full list based on absolute MS intensity (appearing in top 20 for at least one condition); this is an arbitrary reduction of data for more detailed display, and it is important to note that no conclusion should be drawn on the significance of this selection due to the non-linear relationship between abundance and intensity. Intensities are averaged over experimental and analytical replicates.*

m/z	RT	Alumina	Montmoril.	Mica	Goethite	Quartz	Natrolite	Silica
515.8902	**1.14**	185	78	310071	4921	393	97	157
505.8861	**1.14**	216	83	613655	6882	636	121	218
528.8886	**1.14**	162	91	334769	4334	382	61	152
523.8958	**1.14**	189	53	623647	6700	498	89	206
532.9017	**1.14**	280	89	578900	8100	669	104	231
514.8905	**1.15**	771	176	2671729	32395	2305	356	714
182.9022	**1.33**	1405	2779	40968	1180	77493	938129	13335
410.8026	**1.33**	172	191	137	4143	325	72328	190
154.9073	**1.33**	337	441	6695	210	12237	140056	2254
181.9025	**1.33**	562	887	6174	2370	11727	137192	2148
180.9015	**1.33**	255	430	3994	2566	7377	91702	1405
179.9021	**1.33**	222	349	3436	1159	6429	76960	1260
215.6010	**1.33**	304632	5532	92751	1542	336540	2333	138847
244.1118	**1.34**	381586	6170	104192	3535	410070	1199	136826
282.8828	**1.35**	438	589	394	370686	1403	148	196
246.8617	**1.36**	399	872	419	432553	2090	352	244
264.8731	**1.36**	424	667	372	375514	1603	155	176
152.5797	**1.40**	357541	16051	134391	3551	341267	6916	128251
147.0716	**1.40**	740358	51620	398704	10448	749182	9076	350371
350.1566	**1.41**	784568	33536	260495	4827	769266	5518	260848
168.0768	**1.44**	19697	345645	31007	453617	19130	24846	117762
95.0607	**1.47**	145157	289066	138974	342833	146885	38298	234199
175.5824	**1.48**	546595	27370	201717	5460	404957	7829	213271
311.1458	**1.48**	179388	157869	195744	153345	114290	46401	253705
111.0742	**1.48**	225188	77487	150542	79025	241627	39939	165199
407.1786	**1.48**	434421	15572	150927	1370	319420	1372	187852
182.0917	**1.49**	13042	169030	21222	201906	24374	17222	27646
275.1253	**1.50**	1680287	320607	848873	36275	1332334	13956	1264051
267.1082	**1.53**	2662	1649	1881	1145	3661	97539	2443
421.1932	**1.56**	382467	27989	153837	2216	449483	2512	149212
156.0765	**1.59**	2024089	724691	1831942	1023374	2309911	419797	1880253
175.0974	**1.63**	81035	247721	242281	48134	42781	20891	244586
167.0925	**1.64**	173668	138856	220184	59014	198455	7332	91836
478.2160	**1.64**	268450	14308	100801	671	291750	809	105140
157.0793	**1.65**	155643	64398	114414	66553	150392	29600	122255
248.1140	**1.66**	90834	509572	519967	287227	72684	14117	1276975
227.1133	**1.67**	379633	87850	175657	53876	358354	25397	183543
270.1189	**1.71**	476984	57820	251635	12659	584752	30845	208252
211.1010	**1.72**	219607	14722	77880	18768	339656	4001	102547
239.6115	**1.77**	338982	11252	101541	2438	284942	1909	139587
213.0977	**1.78**	311076	85252	166383	58108	121052	113360	351759
196.0901	**1.80**	287575	163615	231349	31968	163565	3560	457434
195.0876	**1.80**	3560203	2007331	2897008	390669	2019202	34010	5572833
110.0715	**1.81**	1035866	687981	446650	1142986	2783821	131242	1759590
284.1340	**1.90**	336839	39968	157867	13967	320152	8342	173959
184.0717	**1.98**	32902	214129	21188	352553	33487	30513	119416
241.0928	**2.05**	21817	132535	18589	174107	27104	4429	90458
181.1078	**2.08**	300721	152646	225367	36155	175437	2490	449602
209.1030	**2.22**	3893142	2101418	2971439	380200	1969699	28795	6189056
210.1054	**2.23**	348576	181170	275068	40840	195902	3370	552761
209.0669	**2.29**	1291658	694515	1016808	119483	689164	11990	878018
227.0769	**2.30**	29228	49306	16908	200786	15244	1583	248325
180.0765	**2.95**	10720	71250	3826	660407	7113	1453	38173
198.0868	**3.30**	6111	115601	4644	141804	4203	612	56031
269.1241	**3.76**	9435	76840	2128	1098840	5416	726	39584
156.0767	**3.77**	21543	62783	7596	574864	22279	1512	69405
211.0686	**4.66**	1599	26368	941	342118	1604	409	9850
180.0763	**5.35**	3083	26431	941	343724	2102	504	19676
269.1241	**5.35**	5368	36910	1708	497269	4103	507	32830
180.0764	**6.38**	4334	44252	1767	424188	2994	463	47418
269.1236	**6.38**	4850	45283	2195	535933	3111	607	60119
156.0764	**7.40**	185999	99617	67575	121150	248873	12003	664988
110.0713	**7.41**	364164	351148	84117	568845	435159	11725	958289
296.1212	**8.21**	1314	17654	686	380718	1428	388	19261
246.2419	**13.84**	34852	34129	33625	33828	33949	34868	32923
274.2736	**15.32**	73172	73482	73860	75945	74789	75705	73976

Figure S16. *Table of selected features ordered by RT from the experiments where the mineral present was varied. Features were selected from a full list based on absolute MS intensity (appearing in top 20 for at least one condition); this is an arbitrary reduction of data for more detailed display, and it is important to note that no conclusion should be drawn on the significance of this selection due to the non-linear relationship between abundance and intensity. Intensities are averaged over experimental and analytical replicates.*

m/z	RT	Alumina	Montmoril.	Mica	Goethite	Quartz	Natrolite	Silica
**190.0821**	106.33	81845	14625	64412	2696	63245	663	28069
**204.0917**	99.46	219778	11223	91490	21652	261853	3306	97548
**204.0962**	127.55	60094	19200	46597	17070	52550	697	47758
**218.1088**	109.19	55221	65487	23351	55581	63822	1646	35161
**218.1124**	165.03	43244	23870	29818	10113	35623	414	40421
**261.1164**	107.09	78025	16103	38199	3676	94540	851	40628
**261.1189**	130.35	48288	14500	31787	2333	52898	326	31099
**270.1166**	446.33	12546	10604	4614	22820	12965	739	37557
**270.1189**	102.58	476984	57820	251635	12659	584752	30845	208252
**270.1193**	203.53	4498	3642	1570	11178	4786	190	5796
**270.1253**	437.36	7112	7826	2475	22493	8374	302	13584
**270.1260**	382.91	1335	4881	498	73487	1266	174	8236
**270.1262**	225.49	2809	10693	969	144392	2762	182	8725
**270.1265**	320.95	1393	5365	613	69264	1364	200	5434
**275.1253**	90.10	1680287	320607	848873	36275	1332334	13956	1264051
**275.1331**	160.07	76346	25521	43215	5518	80817	465	56189
**284.1340**	114.07	336839	39968	157867	13967	320152	8342	173959
**298.1506**	121.59	63768	11648	64090	10665	54499	909	84583
**318.1343**	103.55	32158	5603	10901	4434	33418	738	12331
**327.1409**	105.98	178029	25541	96241	1735	189785	3062	97626
**327.1484**	302.93	1444	9041	546	40870	1214	126	5067
**327.1486**	368.38	1657	7770	602	46555	1385	203	3226
**332.1463**	81.18	80929	4818	18979	2747	102345	876	35578
**341.1558**	435.57	10500	4917	2327	13382	11155	101	7557
**341.1559**	129.56	221120	24203	90918	4794	220460	3278	98063
**341.1613**	455.36	3939	11639	836	37175	2328	150	7381
**341.1642**	380.41	1520	5570	551	33667	1799	116	5046
**341.1642**	455.34	4847	11560	1037	33941	2998	189	10215
**350.1566**	84.62	784568	33536	260495	4827	769266	5518	260848
**355.1716**	125.59	105409	16154	51034	3063	97258	496	51696
**364.1713**	88.05	265593	25441	128196	6017	219282	3101	111938
**384.1613**	111.56	86028	20459	45497	72	119769	356	43594
**398.1759**	126.59	80955	22512	52355	1015	77728	363	56508
**398.1835**	454.95	3730	18770	1417	31025	4187	199	11083
**407.1786**	89.09	434421	15572	150927	1370	319420	1372	187852
**412.1975**	463.38	873	3593	347	14039	920	108	2437
**421.1932**	93.83	382467	27989	153837	2216	449483	2512	149212
**441.1834**	102.61	137738	10334	74373	578	144104	458	67026
**455.1984**	124.55	117057	9560	41989	409	109966	176	41297
**464.1984**	91.94	170895	8581	81254	677	187251	876	82476
**478.2160**	98.11	268450	14308	100801	671	291750	809	105140
**487.2164**	80.10	93232	840	14317	324	93229	218	38993
**492.2312**	105.05	66624	6390	36886	734	109139	303	36040
**535.2355**	103.62	164530	6789	46567	538	175398	338	59685

Figure S17. *Table of selected features ordered by m/z from the experiments where the mineral present was varied. Features were selected from a full list based on absolute MS intensity, filtered to include masses consistent with peptides, (appearing in top 20 for at least one condition); this is an arbitrary reduction of data for more detailed display, and it is important to note that no conclusion should be drawn on the significance of this selection due to the non-linear relationship between abundance and intensity. Intensities are averaged over experimental and analytical replicates.*

m/z	RT	Alumina	Montmoril.	Mica	Goethite	Quartz	Natrolite	Silica
487.2164	**80.10**	93232	840	14317	324	93229	218	38993
332.1463	**81.18**	80929	4818	18979	2747	102345	876	35578
350.1566	**84.62**	784568	33536	260495	4827	769266	5518	260848
364.1713	**88.05**	265593	25441	128196	6017	219282	3101	111938
407.1786	**89.09**	434421	15572	150927	1370	319420	1372	187852
275.1253	**90.10**	1680287	320607	848873	36275	1332334	13956	1264051
464.1984	**91.94**	170895	8581	81254	677	187251	876	82476
421.1932	**93.83**	382467	27989	153837	2216	449483	2512	149212
478.2160	**98.11**	268450	14308	100801	671	291750	809	105140
204.0917	**99.46**	219778	11223	91490	21652	261853	3306	97548
270.1189	**102.58**	476984	57820	251635	12659	584752	30845	208252
441.1834	**102.61**	137738	10334	74373	578	144104	458	67026
318.1343	**103.55**	32158	5603	10901	4434	33418	738	12331
535.2355	**103.62**	164530	6789	46567	538	175398	338	59685
492.2312	**105.05**	66624	6390	36886	734	109139	303	36040
327.1409	**105.98**	178029	25541	96241	1735	189785	3062	97626
190.0821	**106.33**	81845	14625	64412	2696	63245	663	28069
261.1164	**107.09**	78025	16103	38199	3676	94540	851	40628
218.1088	**109.19**	55221	65487	23351	55581	63822	1646	35161
384.1613	**111.56**	86028	20459	45497	72	119769	356	43594
284.1340	**114.07**	336839	39968	157867	13967	320152	8342	173959
298.1506	**121.59**	63768	11648	64090	10665	54499	909	84583
455.1984	**124.55**	117057	9560	41989	409	109966	176	41297
355.1716	**125.59**	105409	16154	51034	3063	97258	496	51696
398.1759	**126.59**	80955	22512	52355	1015	77728	363	56508
204.0962	**127.55**	60094	19200	46597	17070	52550	697	47758
341.1559	**129.56**	221120	24203	90918	4794	220460	3278	98063
261.1189	**130.35**	48288	14500	31787	2333	52898	326	31099
275.1331	**160.07**	76346	25521	43215	5518	80817	465	56189
218.1124	**165.03**	43244	23870	29818	10113	35623	414	40421
270.1193	**203.53**	4498	3642	1570	11178	4786	190	5796
270.1262	**225.49**	2809	10693	969	144392	2762	182	8725
327.1484	**302.93**	1444	9041	546	40870	1214	126	5067
270.1265	**320.95**	1393	5365	613	69264	1364	200	5434
327.1486	**368.38**	1657	7770	602	46555	1385	203	3226
341.1642	**380.41**	1520	5570	551	33667	1799	116	5046
270.1260	**382.91**	1335	4881	498	73487	1266	174	8236
341.1558	**435.57**	10500	4917	2327	13382	11155	101	7557
270.1253	**437.36**	7112	7826	2475	22493	8374	302	13584
270.1166	**446.33**	12546	10604	4614	22820	12965	739	37557
398.1835	**454.95**	3730	18770	1417	31025	4187	199	11083
341.1642	**455.34**	4847	11560	1037	39941	2998	189	10215
341.1613	**455.36**	3939	11639	836	37175	2328	150	7381
412.1975	**463.38**	873	3593	347	14039	920	108	2437

Figure S18. *Table of selected features ordered by RT from the experiments where the mineral present was varied. Features were selected from a full list based on absolute MS intensity, filtered to include masses consistent with peptides, (appearing in top 20 for at least one condition); this is an arbitrary reduction of data for more detailed display, and it is important to note that no conclusion should be drawn on the significance of this selection due to the non-linear relationship between abundance and intensity. Intensities are averaged over experimental and analytical replicates.*

m/z	RT	G > A > H	G > H > A	A > G > H	A > H > G	H > G > A	H > A > G	G + A + H
**110.0714**	2.89	471128	543529	536493	265449	451612	272511	645694
**110.0714**	7.35	667261	838782	654055	471790	435025	398691	1038814
**110.0716**	1.40	4310865	12937166	4929553	14183757	11389903	15484259	9653760
**115.0503**	2.09	838965	682842	432764	159365	390690	216411	405412
**124.0691**	1.38	427297	622792	351939	525940	629125	682310	480923
**147.0721**	1.42	1053929	1307296	1135616	861143	965312	694040	930390
**147.0762**	2.32	474473	376912	704564	268544	415692	348935	526425
**152.5798**	1.42	255707	572162	242183	271360	626590	233528	576079
**156.0768**	1.49	1732736	3069003	6133024	2393722	3222443	2499476	4856198
**167.0926**	1.79	425341	802161	208834	223789	623431	309748	494064
**175.5825**	1.62	152386	946728	478523	518423	1113972	287992	507549
**181.1081**	2.20	522636	297973	119726	318220	100901	501177	256897
**182.5903**	1.72	532747	355364	261112	467896	276846	630746	571794
**195.0876**	1.80	2364110	3146026	664353	966257	1695550	1637330	2154442
**209.1032**	2.21	5976109	2965291	844627	2552330	702686	4923785	2246411
**211.1012**	1.78	542947	618478	454928	462284	486232	450113	757126
**213.0982**	1.85	559593	524542	809297	307778	577368	330075	518951
**215.6014**	1.34	1071839	1375281	987155	1667987	1125751	2014942	1068491
**239.6117**	2.10	529095	567038	875981	111648	113484	101609	531090
**244.1122**	1.35	575542	1567016	631913	611350	1418008	628841	1242060
**247.1299**	1.38	241005	414426	216241	453906	384545	548599	329979
**251.1193**	1.38	301352	198747	269873	1089853	153931	1090442	351229
**268.1224**	1.97	278874	680959	291488	351853	813757	211222	629518
**274.2742**	15.33	994574	725904	721863	572348	638025	799601	739803
**275.1255**	1.51	1298691	1327991	1395821	1906366	1297633	2696740	853237
**284.1338**	2.03	229407	517629	565688	299994	686321	437834	523436
**284.1359**	2.13	572644	473667	907752	517481	482644	548557	393422
**319.6496**	1.28	76304	92624	52832	429097	74009	506950	148239
**327.1410**	1.81	710154	557269	406481	250910	262924	144435	521404
**341.1570**	2.17	1275800	871469	1331522	385257	654204	403212	807927
**350.1572**	1.61	1127638	2487113	908794	911294	2544588	1009937	2071712
**398.1777**	1.91	3053892	757155	2480547	785277	1069829	475137	639337
**399.1802**	2.09	582613	88955	441184	144940	52256	62777	199140
**407.1786**	1.50	452036	1034357	549936	396684	1011150	314605	967019
**412.1932**	2.32	627135	153603	400534	140334	106363	163610	300995
**421.1929**	1.61	581778	586913	762372	846818	492980	363513	745658
**430.1946**	1.31	372267	551536	296702	531549	441449	787063	317248
**435.2102**	1.76	180496	165802	132560	903633	85607	688989	185113
**455.1968**	2.05	1350796	330202	1096215	247684	86830	208250	705904
**455.2000**	2.23	933996	339872	734942	139844	275646	136322	570742
**464.2003**	1.53	187840	623974	343493	148254	596959	96734	361115
**478.2153**	1.86	588171	506087	647919	433932	401571	362650	706492
**487.2154**	1.34	322126	1227204	305573	364005	1029196	415941	871706
**501.2325**	1.37	226611	169836	169664	898684	118565	924449	290690
**535.2378**	1.83	513340	553150	548248	224086	469476	184038	718262

Figure S19. *Table of selected features ordered by m/z from the experiments where the order of addition was varied. Features were selected from a full list based on absolute MS intensity (appearing in top 20 for at least one condition); this is an arbitrary reduction of data for more detailed display, and it is important to note that no conclusion should be drawn on the significance of this selection due to the non-linear relationship between abundance and intensity. Intensities are averaged over experimental and analytical replicates.*

m/z	RT	G > A > H	G > H > A	A > G > H	A > H > G	H > G > A	H > A > G	G + A + H
319.6496	**1.28**	76304	92624	52832	429097	74009	506950	148239
430.1946	**1.31**	372267	551536	296702	531549	441449	787063	317248
487.2154	**1.34**	322126	1227204	305573	364005	1029196	415941	871706
215.6014	**1.34**	1071839	1375281	987155	1667987	1125751	2014942	1068491
244.1122	**1.35**	575542	1567016	631913	611350	1418008	628841	1242060
501.2325	**1.37**	226611	169836	169664	898684	118565	924449	290690
251.1193	**1.38**	301352	198747	269873	1089853	153931	1090442	351229
124.0691	**1.38**	427297	622792	351939	525940	629125	682310	480923
247.1299	**1.38**	241005	414426	216241	453906	384545	548599	329979
110.0716	**1.40**	4310865	12937166	4929553	14183757	11389903	13484259	9653760
152.5798	**1.42**	255707	572162	242183	271360	626590	233528	576079
147.0721	**1.42**	1053929	1307296	1135616	861143	965312	694040	930390
156.0768	**1.49**	1732736	3069003	6133024	2393722	3222443	2499476	4856198
407.1786	**1.50**	452036	1034357	549936	396684	1011150	314605	967019
275.1255	**1.51**	1298691	1327991	1395821	1906366	1297633	2696740	853237
464.2003	**1.53**	187840	623974	343493	148254	596959	96734	361115
350.1572	**1.61**	1127638	2487113	908794	911294	2544588	1009937	2071712
421.1929	**1.61**	581778	586913	762372	846818	492980	363513	745658
175.5825	**1.62**	152386	946728	478523	518423	1113972	287992	507549
182.5903	**1.72**	532747	355364	261112	467896	276846	630746	571794
435.2102	**1.76**	180496	165802	132560	903633	85607	688989	185113
211.1012	**1.78**	542947	618478	454928	462284	486232	450113	757126
167.0926	**1.79**	425341	802161	208834	223789	623431	309748	494064
195.0876	**1.80**	2364110	3146026	664353	966257	1695550	1637330	2154442
327.1410	**1.81**	710154	557269	406481	250910	262924	144435	521404
535.2378	**1.83**	513340	553150	548248	224086	469476	184038	718262
213.0982	**1.85**	559593	524542	809297	307778	577368	330075	518951
478.2153	**1.86**	588171	506087	647919	433932	401571	362650	706492
398.1777	**1.91**	3053892	757155	2480547	785277	1069829	475137	639337
268.1224	**1.97**	278874	680959	291488	351853	813757	211222	629518
284.1338	**2.03**	229407	517629	565688	299994	686321	437834	523436
455.1968	**2.05**	1350796	330202	1096215	247684	86830	208250	705904
115.0503	**2.09**	838965	682842	432764	159365	390690	216411	405412
399.1802	**2.09**	582613	88955	441184	144940	52256	62777	199140
239.6117	**2.10**	529095	567038	875981	111648	113484	101609	531090
284.1359	**2.13**	572644	473667	907752	517481	482644	548557	393422
341.1570	**2.17**	1275800	871469	1331522	385257	654204	403212	807927
181.1081	**2.20**	522636	297973	119726	318220	100901	501177	256897
209.1032	**2.21**	5976109	2965291	844627	2552330	702686	4923785	2246411
455.2000	**2.23**	933996	339872	734942	139844	275646	136322	570742
147.0762	**2.32**	474473	376912	704564	268544	415692	348935	526425
412.1932	**2.32**	627135	153603	400534	140334	106363	163610	300995
110.0714	**2.89**	471128	543529	536493	265449	451612	272511	645694
110.0714	**7.35**	667261	838782	654055	471790	435025	398691	1038814
274.2742	**15.33**	994574	725904	721863	572348	638025	799601	739803

Figure S20. *Table of selected features ordered by RT from the experiments where the order of addition was varied. Features were selected from a full list based on absolute MS intensity (appearing in top 20 for at least one condition); this is an arbitrary reduction of data for more detailed display, and it is important to note that no conclusion should be drawn on the significance of this selection due to the non-linear relationship between abundance and intensity. Intensities are averaged over experimental and analytical replicates.*

m/z	RT	G > A > H	G > H > A	A > G > H	A > H > G	H > G > A	H > A > G	G + A + H
**261.1095**	14.99	6654	8580	5712	5250	8018	5567	6173
**303.1659**	4.35	4360	7439	4387	4066	3351	4617	2466
**318.1495**	1.26	4647	40085	3887	9022	32531	9227	23708
**360.1874**	6.20	6843	1481	11505	1879	834	1941	2495
**369.1870**	7.37	13644	1012	14550	7117	173	6905	2841
**389.1801**	1.24	5127	28436	5235	7555	25747	10203	20515
**421.1879**	1.20	782	5375	476	3523	7016	10662	4384
**441.1834**	3.77	6949	2457	11036	815	1020	735	2331
**503.2205**	6.67	17922	4023	9234	445	1165	370	1171
**517.2361**	5.45	21153	6037	21562	721	2450	596	2620
**526.2279**	1.29	6176	31774	5526	4980	25373	5090	19777
**535.2359**	7.24	2254	3177	4251	1027	2199	758	6080
**540.2512**	7.39	26052	3092	64913	2602	1034	2425	8300
**540.2524**	3.00	9392	1181	11635	681	364	604	2096
**540.2530**	3.82	12129	2249	22842	1237	1005	931	4551
**549.2433**	1.53	15118	10092	3363	11002	4239	35536	3506
**560.2398**	7.31	11881	3317	6207	530	897	454	1551
**560.2424**	4.45	43602	11237	28659	578	2981	500	2717
**589.2328**	2.15	15498	11479	17212	917	4321	685	2504
**603.2478**	3.29	49740	4405	35296	618	1932	482	3016
**603.2479**	2.54	10324	4333	14188	510	1603	489	1605
**617.2628**	7.26	22665	3677	18210	487	1172	463	1999
**617.2642**	4.24	8505	874	12216	236	425	165	1234
**625.3039**	7.55	7465	874	14754	1557	424	1305	3223
**631.2796**	7.54	56211	4217	78758	595	1678	501	5945
**640.2796**	2.68	19249	4022	13964	594	1183	458	2770
**643.3019**	1.70	2265	1637	2384	18354	744	13586	4647
**645.2954**	7.72	19605	1307	36882	449	513	355	3043
**668.3101**	7.46	21435	3018	60204	1578	1665	1228	9722
**674.2857**	7.35	19007	1828	12010	493	531	361	1301
**682.3268**	7.58	5430	577	12287	587	381	489	2030
**683.2855**	2.67	47599	18490	20066	512	2814	397	3264
**688.3007**	7.52	26192	1836	34844	450	530	357	2146
**695.3210**	1.82	6657	2889	7200	6008	1670	7735	4774
**702.3161**	7.70	11769	550	19995	295	448	241	1309
**704.3151**	1.23	3521	9112	941	6802	9743	18000	6582
**709.3290**	1.38	4274	4901	3611	37422	3219	33958	10926
**715.3107**	1.99	5135	9036	5957	817	2154	730	4517
**725.3310**	7.47	12126	1947	36525	729	850	615	4449
**749.3149**	2.41	30671	22498	9519	522	4333	388	1566
**761.3324**	1.22	958	17738	650	3889	17126	10013	8537
**763.3274**	2.66	11333	5995	5603	336	2317	231	3309
**768.3379**	7.38	15373	2268	27225	474	1007	359	3749
**772.3250**	1.63	7021	30100	3258	1611	22554	1150	11415
**775.3511**	1.24	678	939	140	6997	1100	15091	1772
**782.3533**	7.52	7457	419	18283	287	292	281	1571
**795.3382**	1.38	7345	54638	5588	5234	50632	5188	30129
**809.3562**	1.45	2896	10600	4756	5111	9392	4598	9731
**818.3554**	1.24	659	19509	567	1813	13690	4682	8101
**829.3456**	1.68	3187	18174	2060	702	13259	584	7975
**841.3808**	1.53	6471	4977	14277	13589	4687	9460	11581
**852.3630**	1.43	6374	18296	2588	4935	17179	5556	10408
**875.3790**	1.25	620	15059	455	959	11457	1594	9515

Figure S21. *Table of selected features ordered by m/z from the experiments where the order of addition was varied. Features were selected from a full list based on absolute MS intensity, filtered to include masses consistent with peptides, (appearing in top 20 for at least one condition); this is an arbitrary reduction of data for more detailed display, and it is important to note that no conclusion should be drawn on the significance of this selection due to the non-linear relationship between abundance and intensity. Intensities are averaged over experimental and analytical replicates.*

m/z	RT	G > A > H	G > H > A	A > G > H	A > H > G	H > G > A	H > A > G	G + A + H
421.1879	**1.20**	782	5375	476	3523	7016	10662	4384
761.3324	**1.22**	958	17738	650	3889	17126	10013	8537
704.3151	**1.23**	3521	9112	941	6802	9743	18000	6582
389.1801	**1.24**	5127	28436	5235	7555	25747	10203	20515
818.3554	**1.24**	659	19509	567	1813	13690	4682	8101
775.3511	**1.24**	678	939	140	6997	1100	15091	1772
875.3790	**1.25**	620	15059	455	959	11457	1594	9515
318.1495	**1.26**	4647	40085	3887	9022	32531	9227	23708
526.2279	**1.29**	6176	31774	5526	4980	25373	5090	19777
795.3382	**1.38**	7345	54638	5588	5234	50632	5188	30129
709.3290	**1.38**	4274	4901	3611	37422	3219	33958	10926
852.3630	**1.43**	6374	18296	2588	4935	17179	5556	10408
809.3562	**1.45**	2896	10600	4756	5111	9392	4598	9731
841.3808	**1.53**	6471	4977	14277	13589	4687	9460	11581
549.2433	**1.53**	15118	10092	3363	11002	4239	35536	3506
772.3250	**1.63**	7021	30100	3258	1611	22554	1150	11415
829.3456	**1.68**	3187	18174	2060	702	13259	584	7975
643.3019	**1.70**	2265	1637	2384	18354	744	13586	4647
695.3210	**1.82**	6657	2889	7200	6008	1670	7735	4774
715.3107	**1.99**	5135	9036	5957	817	2154	730	4517
589.2328	**2.15**	15498	11479	17212	917	4321	685	2504
749.3149	**2.41**	30671	22498	9519	522	4333	388	1566
603.2479	**2.54**	10324	4333	14188	510	1603	489	1605
763.3274	**2.66**	11333	5995	5603	336	2317	231	3309
683.2855	**2.67**	47599	18490	20066	512	2814	397	3264
640.2796	**2.68**	19249	4022	13964	594	1183	458	2770
540.2524	**3.00**	9392	1181	11635	681	364	604	2096
603.2478	**3.29**	49740	4405	35296	618	1932	482	3016
441.1834	**3.77**	6949	2457	11036	815	1020	735	2331
540.2530	**3.82**	12129	2249	22842	1237	1005	931	4551
617.2642	**4.24**	8505	874	12216	236	425	165	1234
303.1659	**4.35**	4360	7439	4387	4066	3351	4617	2466
560.2424	**4.45**	43602	11237	28659	578	2981	500	2717
517.2361	**5.45**	21153	6037	21562	721	2450	596	2620
360.1874	**6.20**	6843	1481	11505	1879	834	1941	2495
503.2205	**6.67**	17922	4023	9234	445	1165	370	1171
535.2359	**7.24**	2254	3177	4251	1027	2199	758	6080
617.2628	**7.26**	22665	3677	18210	487	1172	463	1999
560.2398	**7.31**	11881	3317	6207	530	897	454	1551
674.2857	**7.35**	19007	1828	12010	493	531	361	1301
369.1870	**7.37**	13644	1012	14550	7117	173	6905	2841
768.3379	**7.38**	15373	2268	27225	474	1007	359	3749
540.2512	**7.39**	26052	3092	64913	2602	1034	2425	8300
668.3101	**7.46**	21435	3018	60204	1578	1665	1228	9722
725.3310	**7.47**	12126	1947	36525	729	850	615	4449
782.3533	**7.52**	7457	419	18283	287	292	281	1571
688.3007	**7.52**	26192	1836	34844	450	530	357	2146
631.2796	**7.54**	56211	4217	78758	595	1678	501	5945
625.3039	**7.55**	7465	874	14754	1557	424	1305	3223
682.3268	**7.58**	5430	577	12287	587	381	489	2030
702.3161	**7.70**	11769	550	19995	295	448	241	1309
645.2954	**7.72**	19605	1307	36882	449	513	355	3043
261.1095	**14.99**	6654	8580	5712	5250	8018	5567	6173

Figure S22. *Table of selected features ordered by RT from the experiments where the order of addition was varied. Features were selected from a full list based on absolute MS intensity, filtered to include masses consistent with peptides, (appearing in top 20 for at least one condition); this is an arbitrary reduction of data for more detailed display, and it is important to note that no conclusion should be drawn on the significance of this selection due to the non-linear relationship between abundance and intensity. Intensities are averaged over experimental and analytical replicates.*

Picked m/z	RT (min)	Int (avg)	*Matched m/z*	*Difference*	Formula
204.0953	1.67	466368	*204.0977*	*-0.0024*	G2A
218.1134	2.62	29046	*218.1133*	*0.0000*	GA2
261.1160	1.78	86010	*261.1192*	*-0.0032*	G3A
270.1215	1.67	442474	*270.1195*	*0.0020*	G2H
275.1326	2.66	62228	*275.1349*	*-0.0022*	G2A2
284.1327	1.91	341876	*284.1351*	*-0.0025*	GAH
298.1533	2.25	66118	*298.1508*	*0.0025*	A2H
327.1430	1.78	188869	*327.1410*	*0.0020*	G3H
341.1565	2.17	160775	*341.1567*	*-0.0001*	G2AH
350.1550	1.41	1267554	*350.1569*	*-0.0019*	GH2
355.1702	1.99	112959	*355.1723*	*-0.0021*	GA2H
364.1755	1.50	615381	*364.1726*	*0.0029*	AH2
369.1904	2.78	18881	*369.1880*	*0.0025*	A3H
384.1650	1.64	234681	*384.1625*	*0.0025*	G4H
398.1789	1.89	199310	*398.1782*	*0.0008*	G3AH
407.1804	1.46	478800	*407.1785*	*0.0019*	G2H2
421.1984	1.58	612085	*421.1941*	*0.0043*	GAH2
430.1945	1.37	147853	*430.1944*	*0.0001*	H3
435.2125	1.70	110105	*435.2098*	*0.0028*	A2H2
487.2180	1.33	278866	*487.2159*	*0.0021*	GH3
567.2564	1.24	15493	*567.2533*	*0.0030*	H4

Figure S23. *Table of consistent peptide compositions for selected features from the experiments where the salts present were varied. Selected features were filtered to include masses consistent with peptides of 3- to 15-mer of G, A and H, and the list of features further refined by selecting only those that appeared in the top 20 (ranked by absolute intensity) for at least one condition in the experiment. Intensity for each feature is in absolute counts (averaged over experimental and analytical replicates). Note: these compositions are only consistent with the mass observed, but no further validation has been carried out. Furthermore, without sequence information little or no conclusion can be drawn from the formulae – see following section.*

Picked m/z	RT (min)	Int (avg)	*Matched m/z*	*Difference*	Formula
190.0821	1.77	81845	*190.0820*	*0.0001*	G3
204.0962	2.13	60094	*204.0977*	*-0.0015*	G2A
218.1124	2.75	43244	*218.1133*	*-0.0010*	GA2
261.1189	2.17	52898	*261.1192*	*-0.0003*	G3A
270.1193	3.39	11178	*270.1195*	*-0.0002*	G2H
275.1331	2.67	80817	*275.1349*	*-0.0017*	G2A2
284.1340	1.90	336839	*284.1351*	*-0.0012*	GAH
298.1506	2.03	84583	*298.1508*	*-0.0002*	A2H
327.1409	1.77	189785	*327.1410*	*-0.0001*	G3H
341.1559	2.16	221120	*341.1567*	*-0.0007*	G2AH
350.1566	1.41	784568	*350.1569*	*-0.0003*	GH2
355.1716	2.09	105409	*355.1723*	*-0.0007*	GA2H
364.1713	1.47	265593	*364.1726*	*-0.0013*	AH2
384.1613	1.86	119769	*384.1625*	*-0.0012*	G4H
398.1759	2.11	80955	*398.1782*	*-0.0023*	G3AH
407.1786	1.48	434421	*407.1785*	*0.0001*	G2H2
412.1975	7.72	14039	*412.1938*	*0.0037*	G2A2H
421.1932	1.56	449483	*421.1941*	*-0.0009*	GAH2
441.1834	1.71	144104	*441.1840*	*-0.0006*	G5H
455.1984	2.08	117057	*455.1997*	*-0.0013*	G4AH
464.1984	1.53	187251	*464.2000*	*-0.0016*	G3H2
478.2160	1.64	291750	*478.2156*	*0.0004*	G2AH2
487.2164	1.33	93232	*487.2159*	*0.0005*	GH3
492.2312	1.75	109139	*492.2313*	*0.0000*	GA2H2
535.2355	1.73	175398	*535.2371*	*-0.0016*	G3AH2

Figure S24. *Table of consistent peptide compositions for selected features from the experiments where the minerals present were varied. Selected features were filtered to include masses consistent with peptides of 3- to 15-mer of G, A and H, and the list of features further refined by selecting only those that appeared in the top 20 (ranked by absolute intensity) for at least one condition in the experiment. Intensity for each feature is in absolute counts (averaged over experimental and analytical replicates). Note: these compositions are only consistent with the mass observed, but no further validation has been carried out. Furthermore, without sequence information little or no conclusion can be drawn from the formulae – see following section.*

Picked m/z	RT (min)	Int (avg)	*Matched m/z*	*Difference*	Formula
303.1659	4.35	7439	*303.1662*	*-0.0002*	A4
360.1874	6.20	11505	*360.1877*	*-0.0003*	GA4
369.1870	7.37	14550	*369.1880*	*-0.0009*	A3H
389.1801	1.24	28436	*389.1779*	*0.0023*	G4A2
441.1834	3.77	11036	*441.1840*	*-0.0006*	G5H
503.2205	6.67	17922	*503.2209*	*-0.0004*	G6A2
517.2361	5.45	21562	*517.2365*	*-0.0005*	G5A3
535.2359	7.24	6080	*535.2371*	*-0.0012*	G3AH2
540.2524	3.00	11635	*540.2525*	*-0.0001*	G3A3H
560.2424	4.45	43602	*560.2424*	*0.0000*	G7A2
589.2328	2.15	17212	*589.2326*	*0.0002*	G10
603.2479	2.54	14188	*603.2483*	*-0.0004*	G9A
617.2642	4.24	12216	*617.2639*	*0.0003*	G8A2
625.3039	7.55	14754	*625.3053*	*-0.0014*	G2A5H
631.2796	7.54	78758	*631.2796*	*0.0000*	G7A3
640.2796	2.68	19249	*640.2798*	*-0.0003*	G6A2H
643.3019	1.70	18354	*643.3059*	*-0.0040*	A3H3
645.2954	7.72	36882	*645.2952*	*0.0002*	G6A4
668.3101	7.46	60204	*668.3111*	*-0.0011*	G4A4H
674.2857	7.35	19007	*674.2854*	*0.0003*	G9A2
682.3268	7.58	12287	*682.3268*	*0.0000*	G3A5H
683.2855	2.67	47599	*683.2857*	*-0.0002*	G8AH
688.3007	7.52	34844	*688.3011*	*-0.0003*	G8A3
702.3161	7.70	19995	*702.3167*	*-0.0006*	G7A4
704.3151	1.23	18000	*704.3123*	*0.0028*	H5
709.3290	1.38	37422	*709.3277*	*0.0013*	A2H4
725.3310	7.47	36525	*725.3327*	*-0.0016*	G5A4H
761.3324	1.22	17738	*761.3338*	*-0.0014*	GH5
763.3274	2.66	11333	*763.3232*	*0.0042*	G7AH2
768.3379	7.38	27225	*768.3385*	*-0.0006*	G7A3H
772.3250	1.63	30100	*772.3234*	*0.0015*	G6H3
775.3511	1.24	15091	*775.3495*	*0.0017*	AH5
782.3533	7.52	18283	*782.3542*	*-0.0009*	G6A4H
795.3382	1.38	54638	*795.3394*	*-0.0012*	G4H4
809.3562	1.45	10600	*809.3550*	*0.0011*	G3AH4
818.3554	1.24	19509	*818.3553*	*0.0001*	G2H5
829.3456	1.68	18174	*829.3450*	*0.0006*	G7H3
852.3630	1.43	18296	*852.3609*	*0.0021*	G5H4
875.3790	1.25	15059	*875.3768*	*0.0021*	G3H5

Figure S25. *Table of consistent peptide compositions for selected features from the experiments where the order of addition was varied. Selected features were filtered to include masses consistent with peptides of 3- to 15-mer of G, A and H, and the list of features further refined by selecting only those that appeared in the top 20 (ranked by absolute intensity) for at least one condition in the experiment. Intensity for each feature is in absolute counts (averaged over experimental and analytical replicates). Note: these compositions are only consistent with the mass observed, but no further validation has been carried out. Furthermore, without sequence information little or no conclusion can be drawn from the formulae – see following section.*

2.2.3 Sequence permutation distribution difference between populations

As outlined above, our aim in LC-MS analysis was to characterise product distribution without the bias/distraction associated with product expectations. We see clearly, both in population-level analyses, and in simple observation of extracted ion chromatograms (EICs) of particular *m/z* values, that product distribution differs clearly and consistently. Since the (secondary & higher) structure and function of oligomeric species depend not only on their composition (e.g. which AAs are incorporated), but also on the sequence of monomers, it is instructive to ask: 'Is the sequence of oligomer products altered by the conditions being manipulated?".

To answer this question unequivocally is difficult; however, it requires identifying and separating very similar species, including those of identical mass. In many cases such isomeric species are extremely difficult to resolve using chromatography – even more so when the chromatography method is general, rather than optimised to resolve specific sequence variants. Below, we show an example where discrete peaks in chromatograms can be assigned to correspond to particular species and demonstrate that different product ensembles can incorporate different sequence permutation distributions (Figure S26; the basis of these assignments explained in Figure 27.

Further examples of sets of isobaric species (likely isomeric/different sequence permutations in many cases) in which both relative and absolute amounts observed in different ensembles vary markedly can be seen in Figures S8 to S10.

(a) (b)

(c) (d) (e)

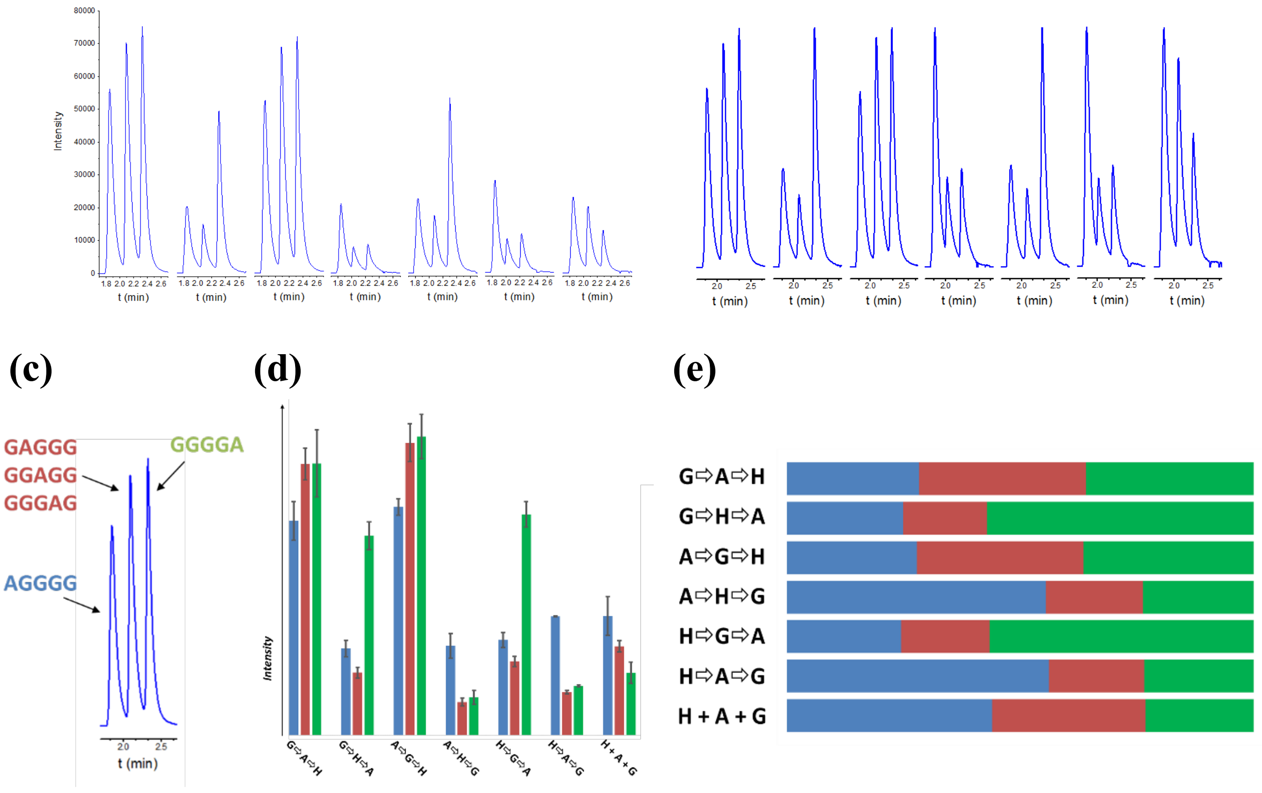

Figure S26. *Plots revealing the sequence permutation distribution of G_4A pentamers. (a) EICs of m/z = 318.141 from products of a mixing history experiment; (b) EICs from part (a) normalised to respective maxima; (c) Identification of sequence permutations contributing to each peak; labels colours followed in intensity plots; (d) Distribution of mean intensity for samples of different mixing histories, with error bars representing one standard deviation; (e) Distribution of mean intensity for samples of different mixing histories, normalised to respective maxima. [EICs were extracted using Bruker Data Analysis; peaks intensities were extracted using Bruker Data analysis as integrated intensities following peak picking; intensity values displayed are means of all 9 data sets (3 experimental reps x 3 analytical reps), with error bars representing one standard deviation]*

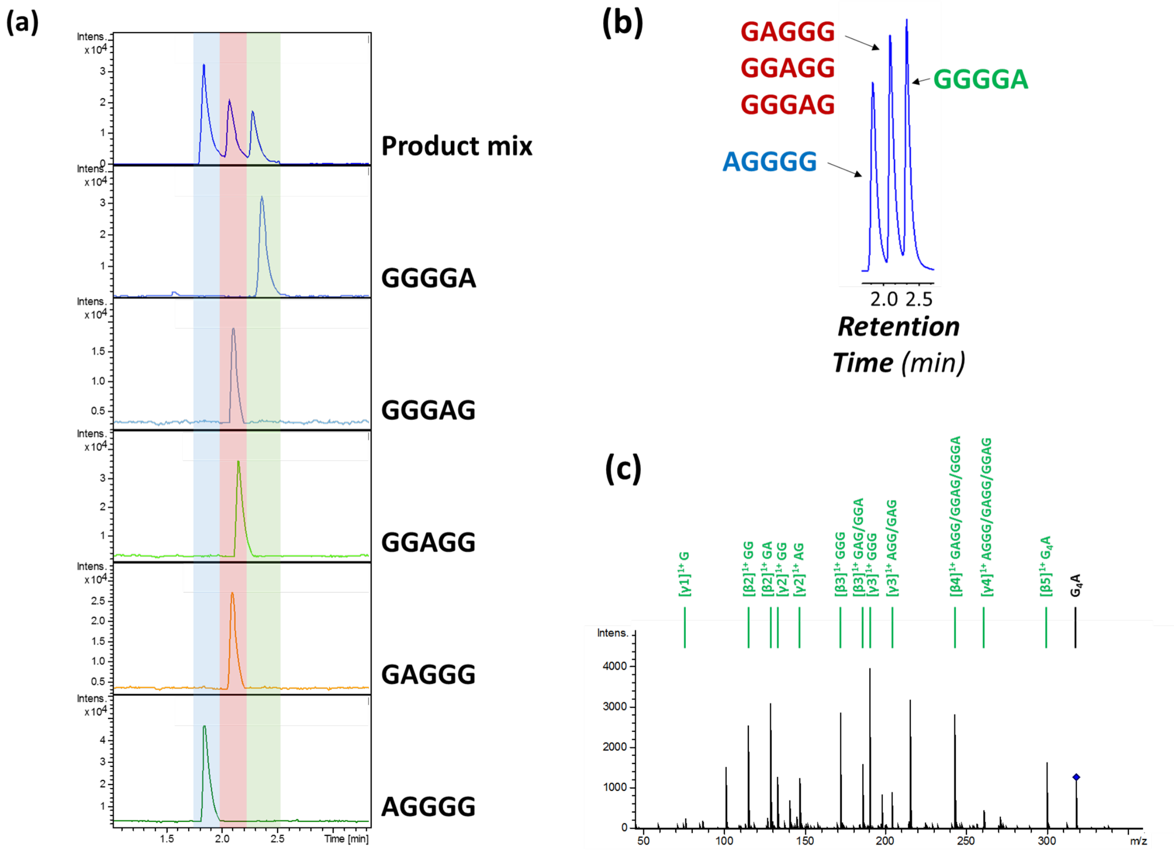

Figure S27. The basis for assignment of the three peaks observed in EICs corresponding to G$_4$A oligomers. *(a) EIC of m/z = 318.1408 +/- 0.002 in a G, A, H condensation product ensemble ("Product Mix") compared to base peak chromatograms of standards of the five sequence permutations possible in G$_4$A pentamers; this comparison confirms the assignment of peak identity shown in (b), and is consistent with data from attempts at* de novo *assignment using MS^2. (c) Example MS^2 spectrum, derived from the fragmentation of m/z = 318.1408 with retention time at 2.1 mins, showing fragmentation consistent with assignment as co-elution of GAGGG, GGAGG, and GGGAG pentamers (middle peak in S27b).*

Note: It was necessary to use synthetic standards (produced by standard SPPS) to confirm the identity of each peak, as robust unequivocal *de novo* assignment is not possible solely based on MS^2 data. MS^2 analysis of each peak did yield fragments consistent with β- and γ- series derived from the sequences finally assigned, however, other peaks were also observed which were consistent with <u>other</u> sequences. For example, MS^2 spectra of the first peak, which corresponds to AGGGG, included a strong peak with *m/z* = 151.0502: this is consistent with GG β-fragment produced from a peptide with an N-terminal GG, but inconsistent with simple β- or γ- fragments of the AGGGG sequence. We speculate that this might result from a Maclaffery Rearrangement.

We include this note to illustrate that robust unequivocal *de novo* assignment of abiotic peptide sequence, where many of the possible sequence permutations are present, is not facile, even in this case with only three monomers (in contrast to biological samples, where complexity is limited, facilitating database approaches). We refrain from drawing conclusions based on such an approach, as they are likely flawed. This – and our intentions to move beyond these simple systems – is the basis for our preferring tools developed for untargeted metabolomics (no specific product expectations), over the more obvious tools developed for proteomics.

2.3 Environment-Directed AA Condensation Experiments: Functional Examination

2.3.1 Reactivity testing using *p*NPA

In these experiments, the effects of product populations on the breakdown of para-*nitrophenyl acetate (*p*NPA, colourless) to release* para-*nitrophenol (*p*NP, yellow) were observed, following this potentially very complex reaction system through the evolution of the yellow colour characteristic of free* p*NP (absorbance at 405 nm).*

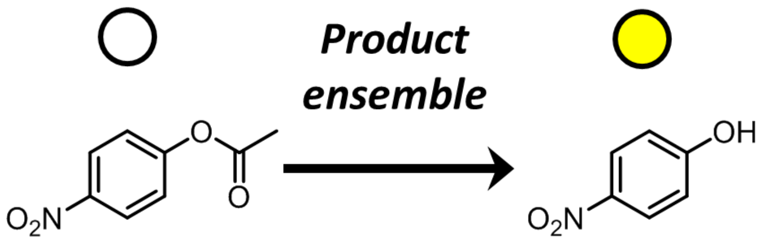

Figure S28. *Breakdown of* p*NPA to yield* p*NP, turning the solution from colourless to yellow.*

<u>Samples</u>: Unless otherwise stated, stock solutions were prepared from previously lyophilised product populations as outlined in Section 2.1, diluting the products of a reaction to a constant volume regardless of amounts of product produced. In some cases (where large amounts were yielded by all conditions tested), solutions of 0.5 mg/ml were also prepared (labelled as 'Constant Concentration, "CC", rather than 'Constant Volume', "CV").

<u>Assay</u>: A buffered substrate solution was prepared by adding 300 µl of *p*-nitrophenyl acetate 0.1 M (in acetonitrile, for ease of handling) and 300 µl of HEPES buffer 1 M to 11400 µl of water. The final amount of acetonitrile present was 1.875%.

150 µl of buffered substrate solution were then added to 50 µl of product ensemble stock solution (giving a final substrate concentration of 1.875 mM). Kinetic measurements were performed in an Infinite M200 Pro Tecan plate reader (using the accompanying software for control and data capture) monitoring the absorbance of the *p*NP at 405 nm and at 25 °C, in a 96-well plate every 5 min for 2 h. At least 12 measurements were collected for each treatment (salt, mineral, mixing history).

<u>Processing</u>: Data was output from the instrument software in a spreadsheet format. Typical time-resolved traces can be observed in Figure S29. Initial rates were extracted using Microsoft Excel as the gradient (not constrained to the origin) of the plot of Abs_{405} (in AU) against time (in seconds) over the first 60 minutes (close to linear in all cases).

Notes:

We note that while this is a common assay for esterase activity, catalysis of ester hydrolysis by the condensation products is not the only possible reaction type. We are interested in the effect of the complete ensemble of products on the reaction system and have made no attempt to identify the mechanism of p*NP release (the complete set of competing pathways may include: ionic strength effects on uncatalysed reaction, inhibition of hydrolysis by recognition, disassembly of active catalytic assemblies on* p*NPA or* p*NP recognition, and other pathways).*

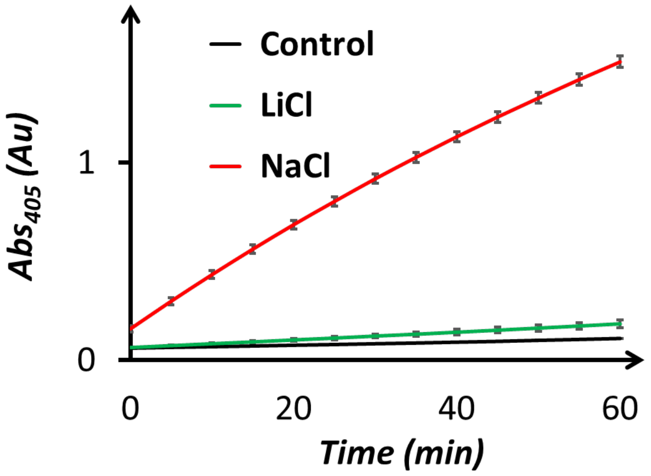

Figure S29. *Example plot of evolution of* p*NP (yellow colour, measured as Abs_{405}) over time, comparing product ensembles formed in the presence of NaCl or LiCl, and a Control lacking any products [error bars represent one standard deviation].*

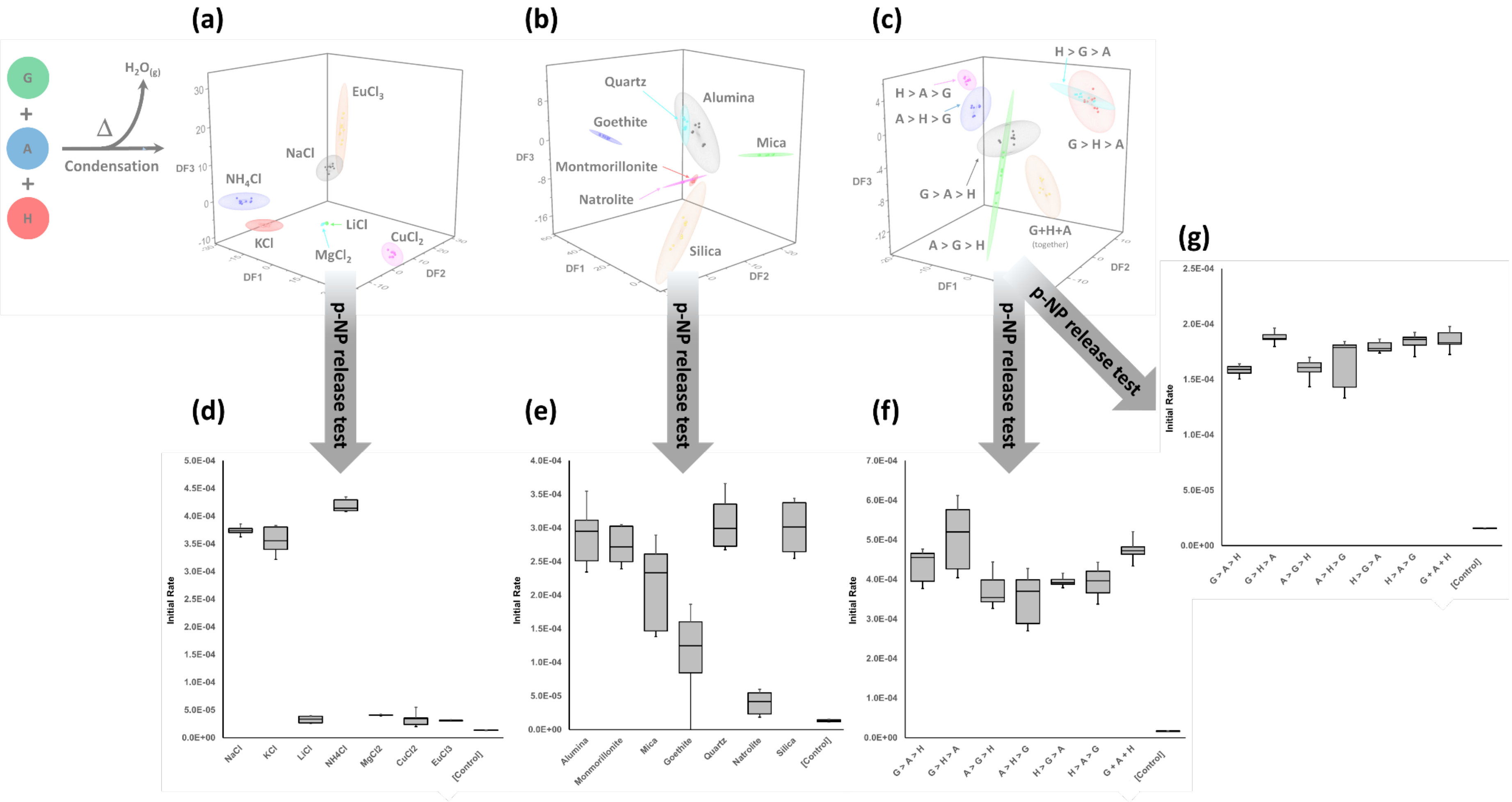

Figure S30 *Results of reactivity testing using* pNPA. *Product ensembles produced with a variety of different environments are represented: (a) varying salts present, in (b) varying minerals present and (c) varying mixing history. Box plots (d), (e) and (f) comparing rates of* pNP *release from the same ensembles. Box plot (g) compares the rate of* pNP *release from ensembles produced from reactions with different mixing histories, diluted to a constant concentration of 0.5 mg/ml (rather than dissolving whatever products are yielded by a reaction to a fixed volume).*

2.3.2 Recognition assay using ThT

This was carried out following an adaptation of an established approach.[15] A stock solution of Thioflavin T (ThT) (Sigma) was prepared by dissolving 8 mg of ThT in 10 ml Tris buffer pH8 (Sigma), followed by filtration through a 0.2 µm syringe filter. The working solution was prepared by diluting the stock into the buffer (1 ml stock to 50 ml buffer). 50 µl of the peptide solution and 20 µl of the ThT working solution were mixed in a 96 well-plate (Thermo Fisher). Fluorescence was measured after one hour of incubation using (Infinite® 200 PRO plate reader) by excitation at 444 nm and emission at 480 nm. Samples were measured in duplicate. Fluorescence values of the samples were compared to the ThT values (as a control).

2.3.3 Inspection of Assembly/Aggregation using TEM

Procedure

Carbon-coated copper grids (200 mesh) were glow discharged in air for 30 seconds. The support film was touched onto the peptide solution surface for 10 seconds, and excess solution was removed using filter paper. 20 µl of negative stain (Nanovan; Nanoprobes) was applied and the mixture was blotted again using filter paper to remove any excess stain. The dried specimens were then imaged using an FEI Tecnai T20 Tranmission Electron Microscope (TEM) operating at 200 kV fitted with Gatan 794 Multiscan camera. Images were collected and converted to .tiff files using Gatan Microscopy Suite software.

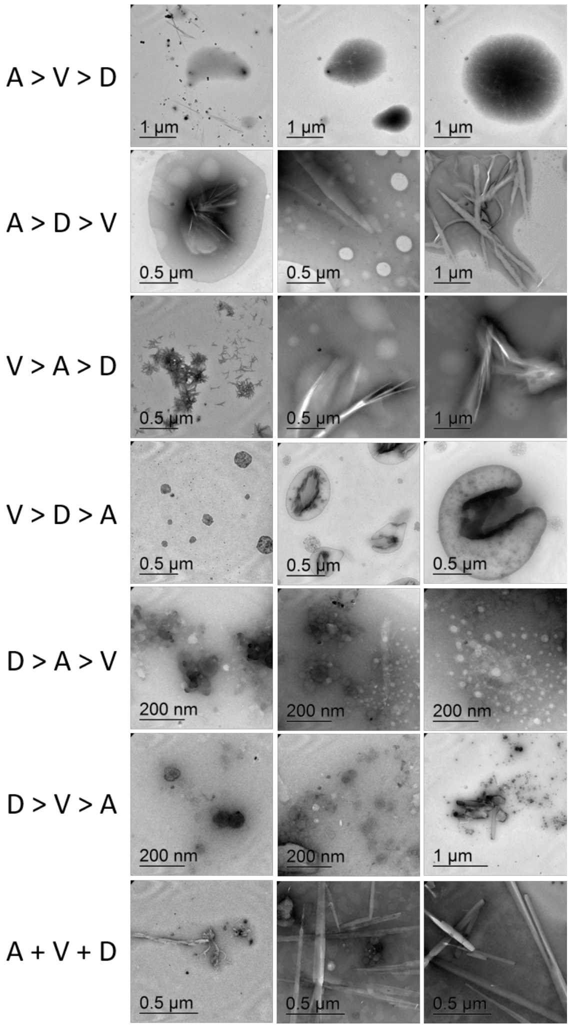

Figure S31. *Additional TEM images.*

2.3.4 Observation of different properties of gels produced on addition of Ca^{2+} salts.

Following the difference in structural formation ability from amino acids' mixing history (Section 2.3.3), the difference of gelability was studied by peptides crosslinking with Ca^{2+}. This was performed by mixing 500 µl of each peptide solution (prepared in Section (2.1.4)) together with 2.5 µl of 1 M $CaCl_2$, vortexing and leaving to stand overnight at room temperature. Gelability was verified by the inverted vial method (see Figure S32, in which those samples which are immobile were persistent in the position shown for periods > 1 h), and the products were observed using TEM following gelation, revealing dramatically different morphology (Figure S33).

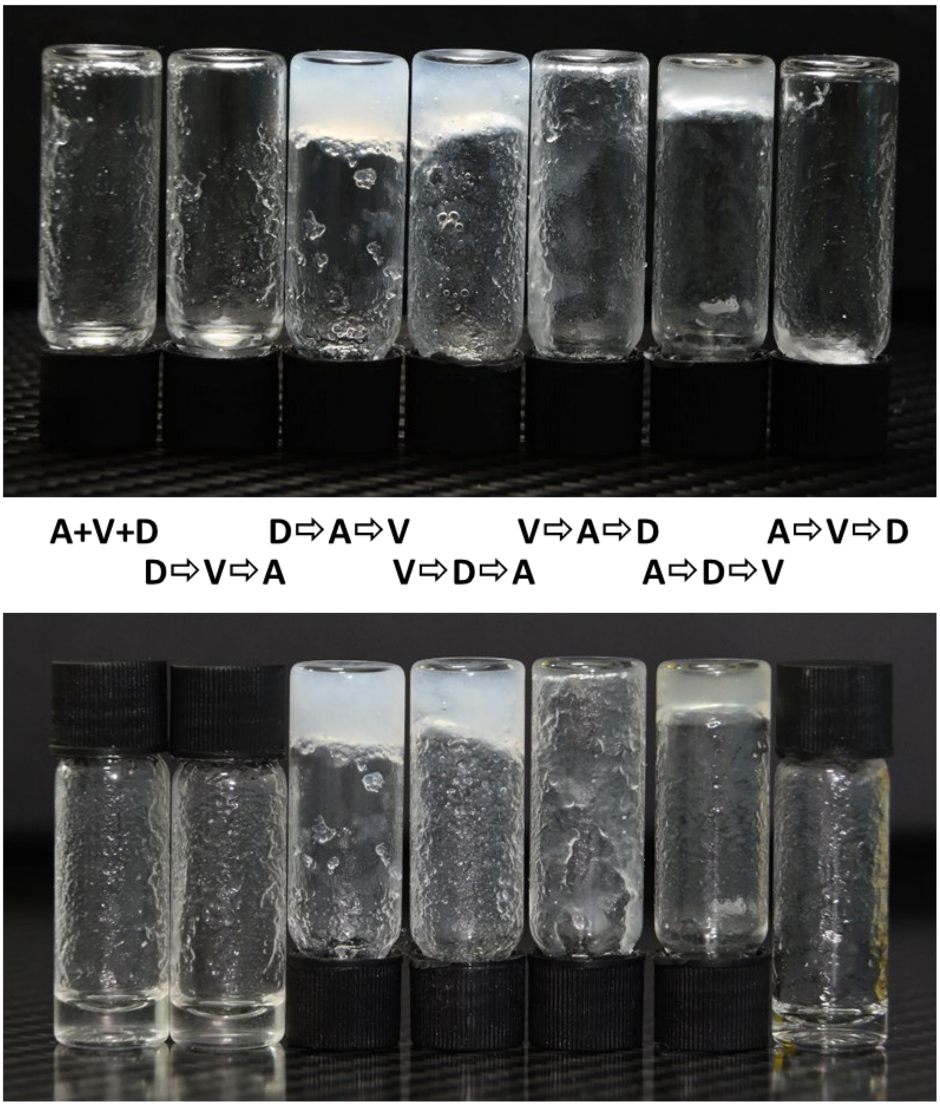

Figure S32. *Different ensembles produce materials with dramatically different degrees of gelation on addition of $CaCl_2$. Note: in image below, it is clear that in product ensembles without persistent gelling, clear solutions are observed rather than weaker gels.*

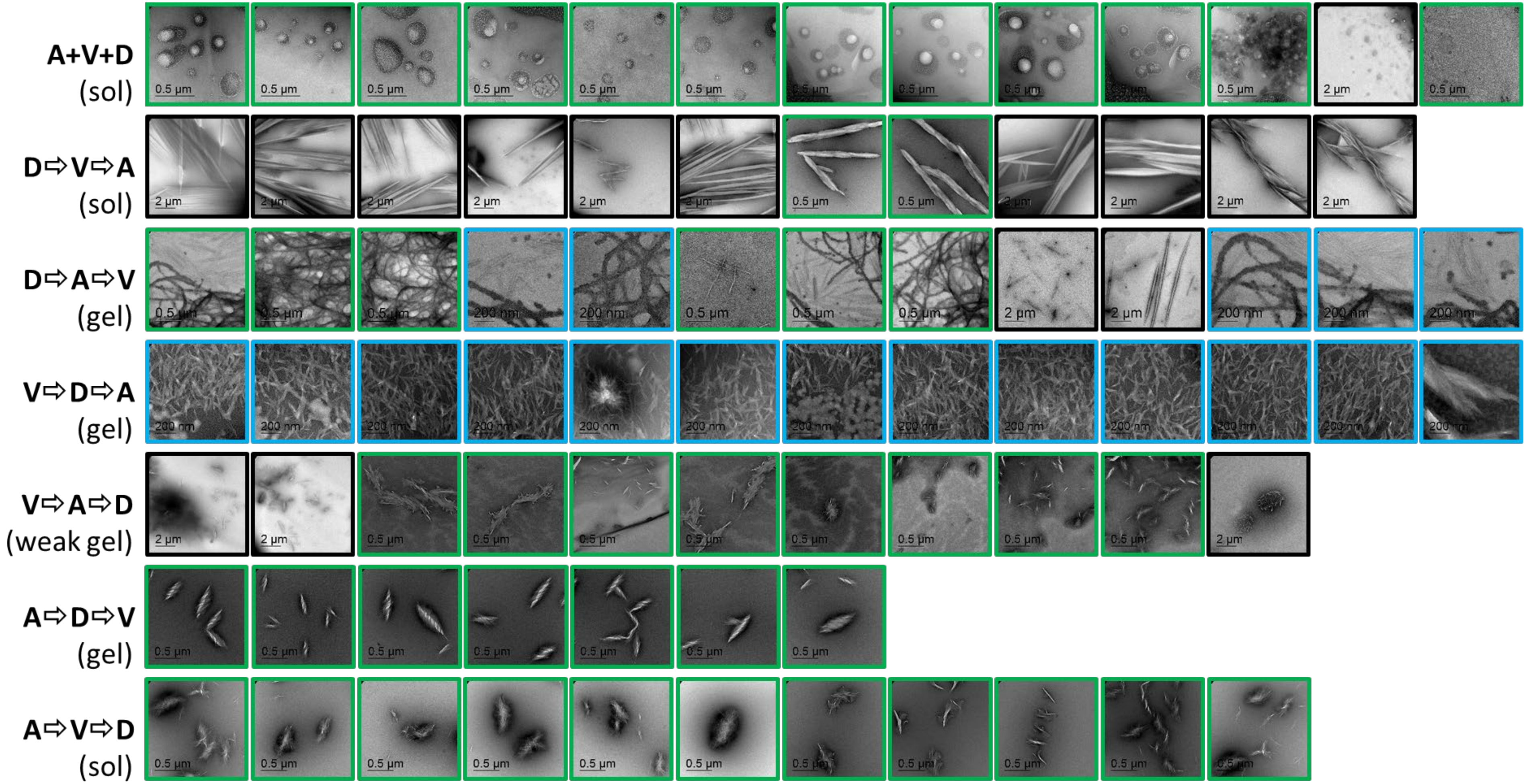

Figure S33. *TEM images showing assembly/aggregation of product ensembles on addition of Ca^{2+} salts (where outline is blue, scale bar = 0.2 μm; where outline is green, scale bar = 0.5 μm; where outline is black, scale bar = 2.0 μm).*

3 Environment-Directed Complex Mixture Condensation Experiments

3.1 Spark Discharge Mixture Preparation

Spark discharge mixtures were chosen as a complex mixture for this study, both as a classic 'intractable' complex mixture,[16] and as they are known to contain a range of species (amino acids, hydroxyl acids, amines, etc.) amenable to simple condensation reactions. The spark discharge reaction product mixture ("SD Mix") used here as a model complex mixture was prepared from the combination of several SD reaction runs, using equipment similar to that of the 1950's Miller-Urey experiment.[17] In each run, after careful cleaning and drying of the glassware, 400 mL of water (LC-MS grade) was added and the system sealed. The whole rig was pumped down three times to de-gas the water and finally after the third evacuation, the system was pressurised to 1 atm with gas mixture (40% methane, 40% ammonia and 20% hydrogen). Heating was applied to the main flask and, once boiling and recirculation was established, the 24 kV spark discharge was applied with a 10 sec alternating duty-cycle. Experiments were run for seven days, during which time the solution in the flask became deep brown in colour.

A total of ca. 0.5 L of product mixture was collected, combining the products of several runs. In order to produce a standardised mixture, free of large amounts of slowly-precipitating SiO_2 (dissolved from glassware), the mixture was then freeze-dried, redissolved in water (LC-MS grade) centrifuged (at 10k rpm for 1 h using a Beckman Coulter Avanti I-E centrifuge) and filtered (Millipore Durapore 0.22 µm, HV type membranes), freeze-dried again and re-filtered (no observable residue). This yielded a light tan-coloured solution, containing approximately 1 mg/ml of soluble material.

3.2 Environment-Directed Complex Mixture Experiments: Synthesis

In this set of experiments, 4 ml of a standard SD mixture (see Section 3.1) was condensed (by dehydration) in the presence of a series of different minerals.

1. 4 ml of the standard SD mixture was added to each reaction vessel (open vial).
2. 0.2 g of a powdered mineral were added to each individual experiment. In addition, a control reaction with no mineral was set up (known as "Control (NM)"), and a control in which no condensation reaction took place (i.e. Step 3 was omitted and the SD mixture was stored at 4 °C) was set up (known as "Control (NR)").
3. A single dehydration step was performed in a fan-assisted oven at 115 °C for 24 h (a fixed arbitrary cycle time; all reactions performed together).
4. Reactions were then removed, and cooled to room temperature.
5. Each individual product mixture was dissolved in 4 ml of water, with sonication to aid dissolution of soluble species.
6. Each individual product mixture solution was then filtered (0.2 µm, Pall Microsep centrifugal filter) to remove minerals and undissolved species.
   [n.b. centrifugal filters used to maximise and standardise product recovery]
7. The filtrate of each reaction (and washings) was dialysed with a G2 Float-a-lyser (100-500 Da) cut-off (5 ml) for 24 h, to remove any small species and soluble salts.
8. Once the dialysis was completed, the samples (and washing, to avoid loss) were left to freeze-dry for 48 h.
9. The product mixtures were then redissolved in 0.5 ml water, and used/analysed without further treatment.

3.3 Environment-Directed Complex Mixture Experiments: Product Analysis

LC-MS analysis was accomplished in an adaptation of the general procedure described in Section 1.2, in which the linear gradient mixture of solvents A (water w/0.1% *v/v* formic acid) and B (acetonitrile w/0.1% *v/v* formic acid) was as follows over 40 min: 0 min – 0% B; 4 min – 0% B; 26 min – 100% B; 30 min – 100% B; 36 min – 0% B.

This LC-MS data was then processed and plotted as described in Section 2.2.1: peak picking and grouping, and gap-filling from raw data where no peaks were observed. PCA was performed on the resulting data as earlier (*m/z* and *rt* coordinates for each feature, with corresponding intensity for each sample), again with scaling. The results of this analysis are shown below, along with some sample EICs illustrating variance.

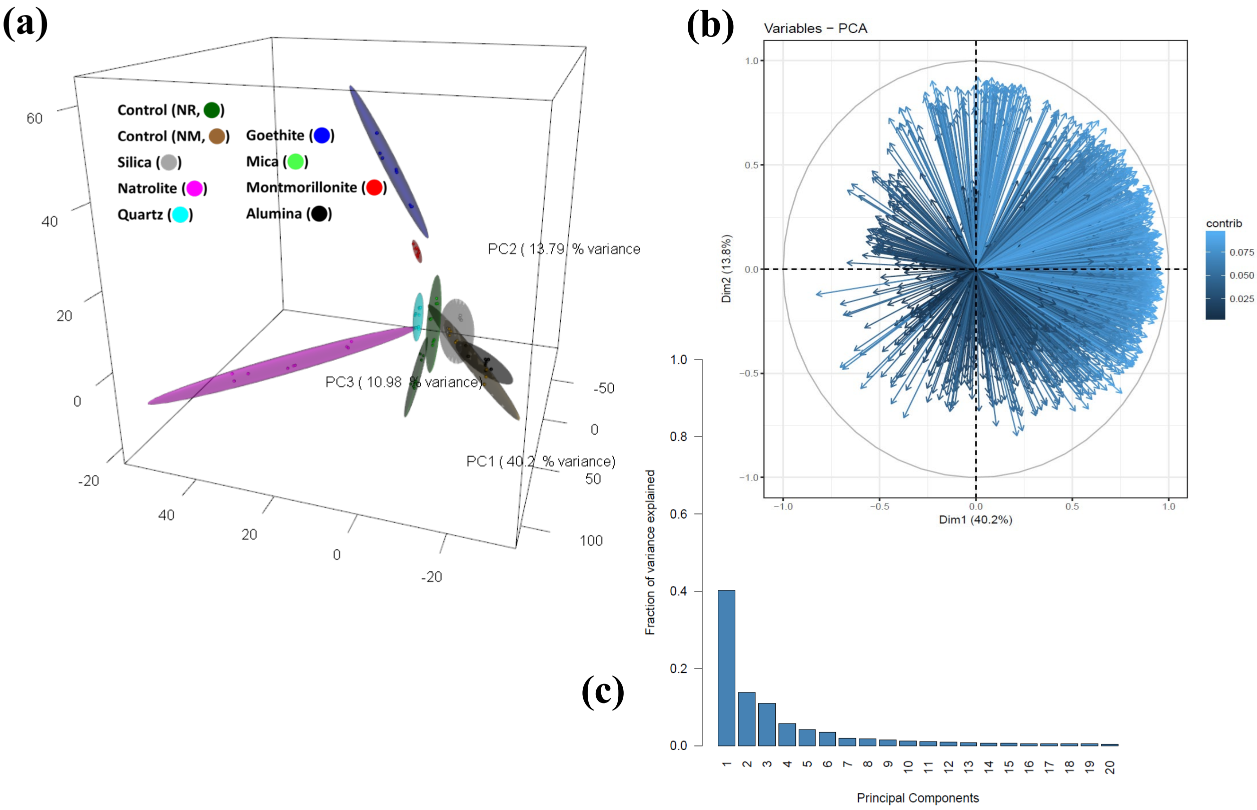

Figure S34 *Plots of PCA analysis of results from condensation of SD mixture in the presence of different minerals.* ***(a)*** *Plot of first two PCs [in each case 'bubbles' represent 95% confidence limits & 'spots' represent individual measurements.]* ***(b)*** *Distribution of contributions to the first two principal components.* ***(c)*** *Plot of fraction of variance explained by these principal components.*

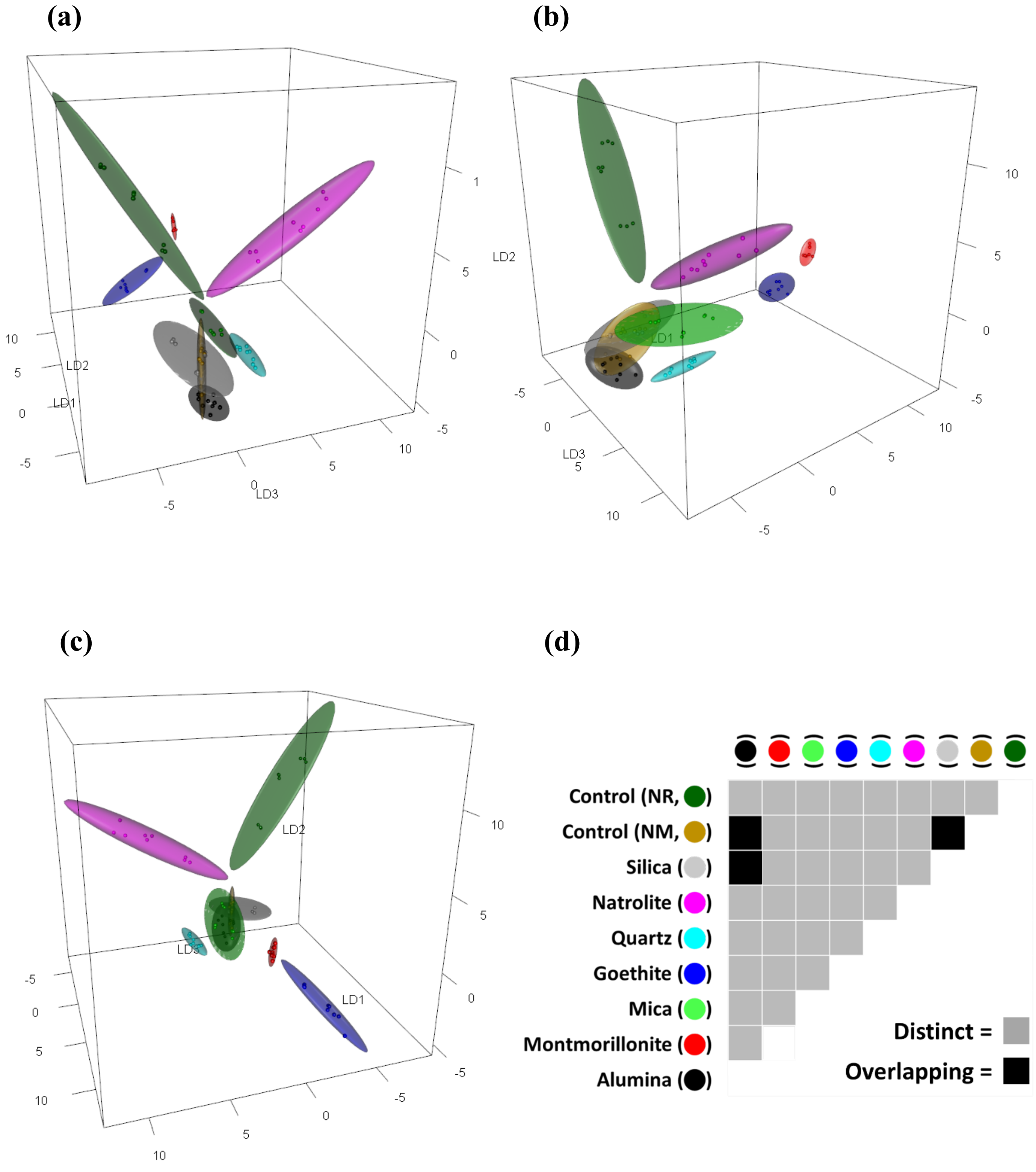

Figure S35 *(a-c) A range of plots (with different perspective) of PC-DFA analysis (using first 5 PCs) of results from condensation of SD mixture in the presence of different minerals; in each case 'spots' represent individual measurements & 'bubbles' represent two standard deviations around their mean. (d) Key to identify product ensembles, denoting mineral in whose presence they were produced, and matrix to clarify which ensembles overlap. Analysis conducted in R, calculated and plotted using rgl library.*

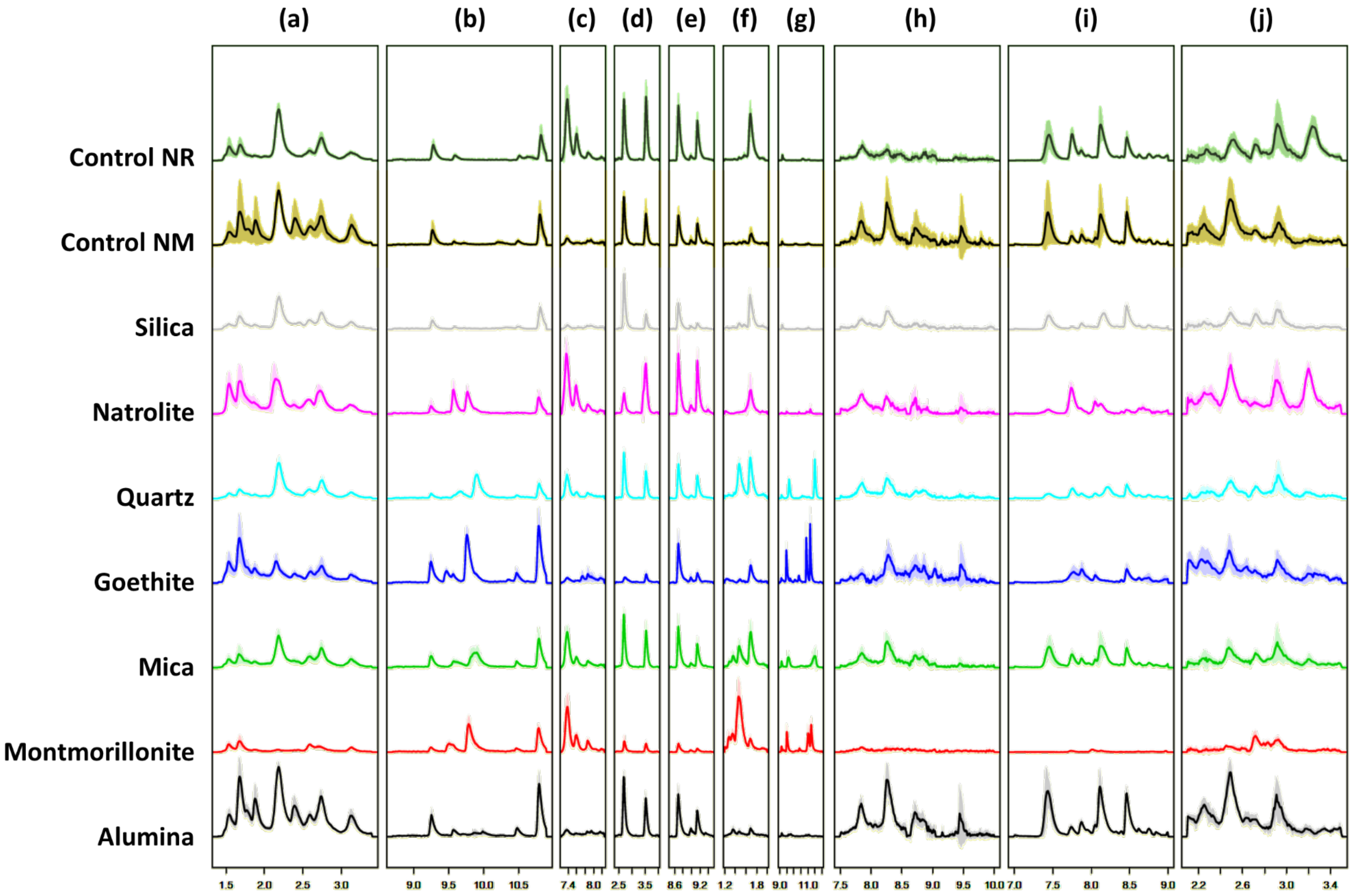

Figure S36. *Selected extracted ion chromatograms illustrating product distribution variance in products from experiments varying minerals present – Part 1 of 2. (a) m/z = 101.0715; (b) m/z = 102.0918; (c) m/z = 142.0507; (d) m/z = 166.0245; (e) m/z = 174.0582; (f) m/z = 176.0138; (g) m/z = 193.1387; (h) m/z = 203.1029; (i) m/z = 208.0464; (j) m/z = 218.1134. [lines = mean intensity from all measurements; shading around line represents one standard deviation around mean; intensities normalised relative to largest value in each plot]*

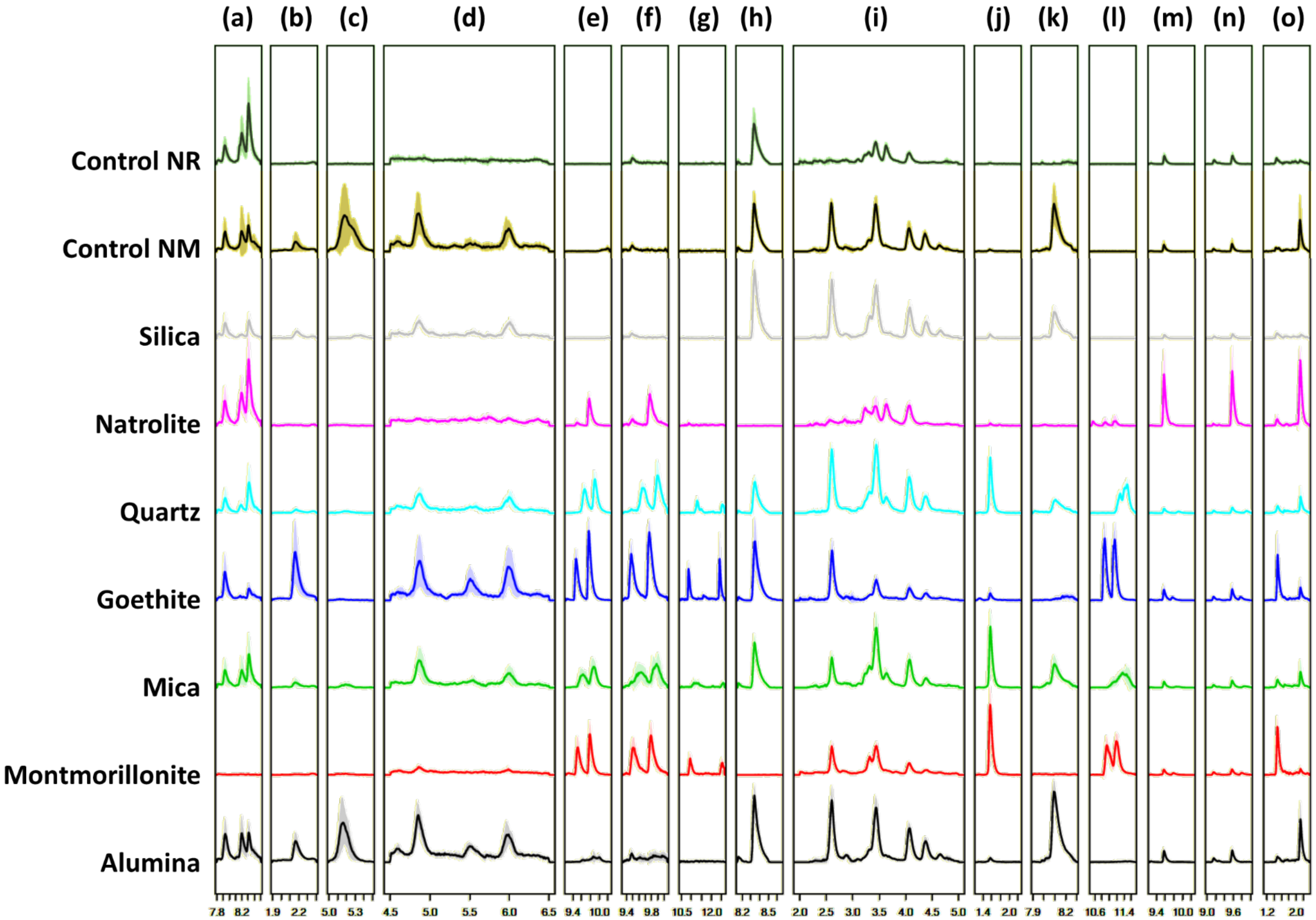

Figure S37. *Selected extracted ion chromatograms illustrating product distribution variance in products from experiments varying minerals present – Part 2 of 2. (a) m/z = 219.0614; (b) m/z = 223.1186; (c) m/z = 230.1610; (d) m/z = 242.0768; (e) m/z = 243.6842; (f) m/z = 244.1907; (g) m/z = 250.1538; (h) m/z = 252.0362; (i) m/z = 255.0590; (j) m/z = 262.0142; (k) m/z = 278.0520; (l) m/z = 300.7007; (m) m/z = 302.1963; (n) m/z = 303.2014; (o) m/z = 321.0014. [lines = mean intensity from all measurements; shading around line represents one standard deviation around mean; intensities normalised relative to largest value in each plot]*

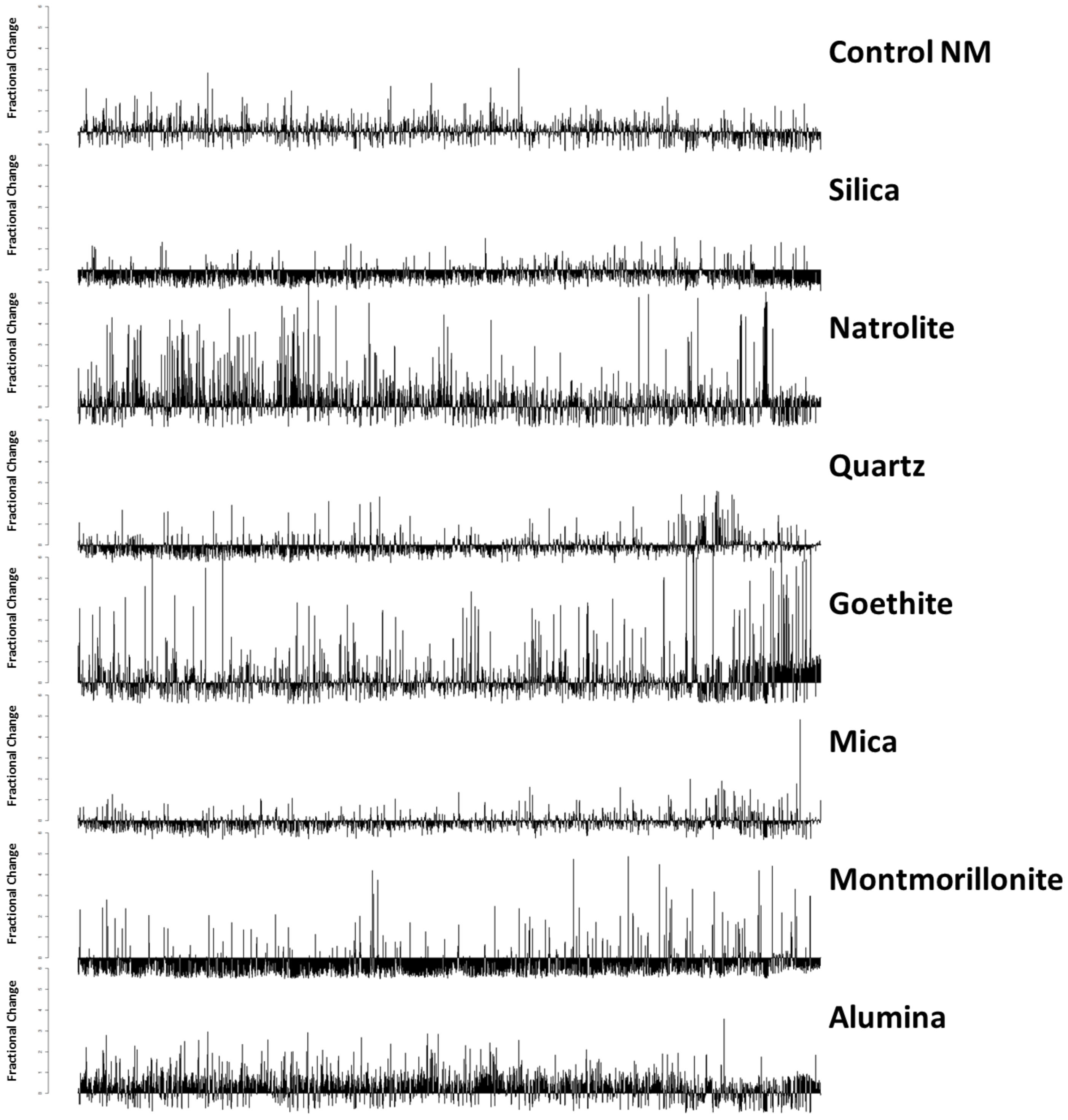

Figure S38. *The intensity of each of the features picked (on which PCA, etc was performed), expressed as a fractional difference from a mean for all the peaks within a set as a means to visualise variation in data.*

[i.e. ($Mean^{Mica} - Mean^{CtrlNM}$) / $Mean^{AllMin}$, where $Mean^{AllMin}$ is the mean intensity across all mineral environments; feature m/z and rt coordinates unlabelled, ordered by ascending m/z from left to right (as ca. 1800 features are plotted, lines are thin); Note: Fractional intensities can obscure smaller variations in intensity values below control]

m/z	RT	Alumina	Montmoril.	Mica	Goethite	Quartz	Natrolite	Silica	Control NM	Control NR
**97.9695**	1.34	4758	69677	33681	16645	16058	2196	5606	4939	5618
**127.0504**	1.81	68551	58297	43788	49177	39972	57185	46366	52738	51409
**134.0449**	1.75	99836	133759	190397	41431	222630	115551	194117	147357	150881
**139.0616**	3.53	152654	30072	144496	35296	111544	251713	55691	132252	255124
**141.9590**	1.34	4782	64603	30740	13840	13666	2288	5275	5135	5525
**146.0449**	1.78	87036	113150	34794	26925	44750	79386	55686	47217	109179
**148.0606**	1.56	120081	150471	175253	63832	155948	99580	146947	127985	140443
**153.0296**	2.24	227431	56357	121572	154220	93307	242439	89482	149671	150780
**154.9075**	1.33	51182	5449	85393	1454	281068	715202	117453	90043	111562
**159.0767**	2.18	589186	11389	263952	138981	294048	375657	266328	471051	407375
**164.9208**	1.41	5530	20731	2830	184940	423	438	2565	4133	2680
**170.0564**	2.24	225008	54874	113421	157426	87229	221947	87470	169678	148950
**171.0401**	2.24	488448	126381	265222	343306	206835	537263	199058	329210	342693
**172.0352**	3.51	129503	72064	54645	90577	39990	95659	52825	99017	76573
**173.0564**	1.82	76873	84218	54309	59553	48571	60996	56391	65510	65845
**178.0618**	7.41	2263628	6311	1034531	879851	663844	3073061	346802	1630504	1486776
**179.0643**	7.41	194134	712	93666	76034	63863	237517	32159	111906	132947
**179.9027**	1.33	42358	4730	67089	309	222620	589515	94597	72904	86542
**180.0520**	7.80	197320	486	108921	2630	45919	258156	39094	115844	128328
**180.8954**	1.42	20781	6367	18010	31555	83774	376297	24561	30865	39662
**180.9018**	1.33	50834	7588	80213	7304	272480	721551	114034	85450	106560
**181.9036**	1.33	75680	8830	122173	564	401927	1105939	171702	124189	160184
**182.9025**	1.33	495035	56202	831671	2357	1050730	6359275	1212096	872916	1100515
**182.9490**	1.42	48943	8708	65921	3760	256589	4500049	258123	184728	259250
**192.0503**	1.52	49502	64079	89095	10870	93252	50045	180044	78468	124572
**194.9665**	1.39	12819	66634	39021	746	72477	273	3946	1858	738
**197.0569**	1.99	7844	117767	6219	5885	5852	9261	20470	4817	12749
**198.0622**	7.81	408193	764	268779	75057	113953	613955	94487	361024	316185
**202.1800**	6.20	257497	86798	142268	253048	68661	191611	116563	168354	153304
**205.0820**	2.17	305726	21289	140575	70704	155592	193292	141139	247349	243870
**211.0693**	7.37	163523	3624	70417	52168	50560	47051	16703	67915	5852
**214.9171**	1.33	29425	35492	38102	137076	48345	4069	50659	24568	26907
**226.9517**	1.43	103206	15894	25314	440	10436	21491	98459	131318	110926
**230.8899**	1.33	4070	59125	34762	29247	16110	934	5968	4360	4787
**236.0672**	9.45	160959	757	11177	302990	12653	2659	113890	72676	1550
**306.8520**	1.42	2793	7342	6861	103547	2500	1044	2275	1492	930
**312.8616**	1.33	39265	2765	42828	3713	172018	110177	69433	62845	58449
**316.2121**	10.80	177734	78867	98923	192092	49484	57021	74131	101567	78077
**409.8053**	1.33	7955	331	11710	307	78316	316181	30331	20724	27234
**410.8048**	1.33	24741	1366	37358	841	251392	1069344	96763	63423	86249
**432.2813**	11.11	109273	42204	58621	119070	37839	94214	42197	76007	27493
**476.3068**	11.37	237263	76414	119518	233922	76433	182286	44337	150087	62969
**520.3320**	11.59	377373	107105	196390	350553	125579	300242	71225	219531	116134
**564.3592**	11.80	465661	120584	238242	387860	161575	329568	89390	199009	156273
**565.3618**	11.80	120140	32883	65085	108573	44280	87258	25189	52615	43625
**608.3844**	11.99	368600	105699	220423	376512	157099	288826	81510	162958	156437
**609.3892**	11.99	104814	31378	62507	107352	46885	83717	24939	48329	47797
**652.4113**	12.16	230354	76527	150709	279921	124915	185245	57244	123853	119766
**696.4368**	12.32	119185	44948	83743	152768	75849	101000	29209	70794	61350

Figure S39. *Table of selected features ordered by RT from the SD-mix experiment with different minerals. Features were selected from a full list based on absolute MS intensity (appearing in top 20 for at least one condition); this is an arbitrary reduction of data for more detailed display, and it is important to note that no conclusion should be drawn on the significance of this selection due to the non-linear relationship between abundance and intensity. Intensities are averaged over experimental and analytical replicates.*

m/z	RT	Alumina	Montmoril.	Mica	Goethite	Quartz	Natrolite	Silica	Control NM	Control NR
214.9171	**1.33**	29425	35492	38102	137076	48345	4069	50659	24568	26907
312.8616	**1.33**	39265	2765	42828	3713	172018	110177	69433	62845	58449
410.8048	**1.33**	24741	1366	37358	841	251392	1069344	96763	63423	86249
409.8053	**1.33**	7955	331	11710	307	78316	316181	30331	20724	27234
230.8899	**1.33**	4070	59125	34762	29247	16110	934	5968	4360	4787
179.9027	**1.33**	42358	4730	67089	309	222620	589515	94597	72904	86542
182.9025	**1.33**	495035	56202	831671	2357	3050730	8359275	1212096	872916	1100515
154.9075	**1.33**	51182	5449	85393	1454	281068	715202	117453	90043	111562
180.9018	**1.33**	50834	7588	80213	7304	272480	721551	114034	85450	106560
181.9036	**1.33**	75680	8830	122173	564	401927	1105939	171702	124189	160184
97.9695	**1.34**	4758	69677	33681	16645	16058	2196	5606	4939	5618
141.9590	**1.34**	4782	64603	30740	13840	13666	2288	5275	5135	5525
194.9665	**1.39**	12819	66634	39021	746	72477	273	3946	1858	738
164.9208	**1.41**	5530	20731	2830	184940	423	438	2565	4133	2680
182.9490	**1.42**	48943	8708	65921	3760	256589	4500049	258123	184728	259250
180.8954	**1.42**	20781	6367	18010	31555	83774	376297	24561	30865	39662
306.8520	**1.42**	2793	7342	6861	103547	2500	1044	2275	1492	930
226.9517	**1.43**	103206	15894	25314	440	10436	21491	98459	131318	110926
192.0503	**1.52**	49502	64079	89095	10870	93252	50045	180044	78468	124572
148.0606	**1.56**	120081	150471	175253	63832	155948	99580	146947	127985	140443
134.0449	**1.75**	99836	133759	190397	41431	222630	115551	194117	147357	150881
146.0449	**1.78**	87036	113150	34794	26925	44750	79386	55686	47217	109179
127.0504	**1.81**	68551	58297	43788	49177	39972	57185	46366	52738	51409
173.0564	**1.82**	76873	84218	54309	59553	48571	60996	56391	65510	65845
197.0569	**1.99**	7844	117767	6219	5885	5852	9261	20470	4817	12749
205.0820	**2.17**	305726	21289	140575	70704	155592	193292	141139	247349	243870
159.0767	**2.18**	589186	11389	263952	138981	294048	375657	266328	471051	407375
170.0564	**2.24**	225008	54874	113421	157426	87229	221947	87470	169678	148950
171.0401	**2.24**	488448	126381	265222	343306	206835	537263	199058	329210	342693
153.0296	**2.24**	227431	56357	121572	154220	93307	242439	89482	149671	150780
172.0352	**3.51**	129503	72064	54645	90577	39990	95659	52825	99017	76573
139.0616	**3.53**	152654	30072	144496	35296	111544	251713	55691	132252	255124
202.1800	**6.20**	257497	86798	142268	253048	68661	191611	116563	168354	153304
211.0693	**7.37**	163523	3624	70417	52168	50560	47051	16703	67915	5852
179.0643	**7.41**	194134	712	93666	76034	63863	237517	32159	111906	132947
178.0618	**7.41**	2263628	6311	1034531	879851	663844	3073061	346802	1630504	1486776
180.0520	**7.80**	197320	486	108921	2630	45919	258156	39094	115844	128328
198.0622	**7.81**	408193	764	268779	75057	113953	613955	94487	361024	316185
236.0672	**9.45**	160959	757	11177	302990	12653	2659	113890	72676	1550
316.2121	**10.80**	177734	78867	98923	192092	49484	57021	74131	101567	78077
432.2813	**11.11**	109273	42204	58621	119070	37839	94214	42197	76007	27493
476.3068	**11.37**	237263	76414	119518	233922	76433	182286	44337	150087	62969
520.3320	**11.59**	377373	107105	196390	350553	125579	300242	71225	219531	116134
565.3618	**11.80**	120140	32883	65085	108573	44280	87258	25189	52615	43625
564.3592	**11.80**	465661	120584	238242	387860	161575	329568	89390	199009	156273
608.3844	**11.99**	368600	105699	220423	376512	157099	288826	81510	162958	156437
609.3892	**11.99**	104814	31378	62507	107352	46885	83717	24939	48329	47797
652.4113	**12.16**	230354	76527	150709	279921	124915	185245	57244	123853	119766
696.4368	**12.32**	119185	44948	83743	152768	75849	101000	29209	70794	61350

Figure S40. *Table of selected features ordered by RT from the SD-mix experiment with different minerals. Features were selected from a full list based on absolute MS intensity (appearing in top 20 for at least one condition); this is an arbitrary reduction of data for more detailed display, and it is important to note that no conclusion should be drawn on the significance of this selection due to the non-linear relationship between abundance and intensity. Intensities are averaged over experimental and analytical replicates.*

Formula assignment for SD mix:

As in our previous work considering patterns in these kinds of complex mixtures, we have made very tentative formula assignments of the most influential features observed, to illustrate the kinds of compositions might be present. We followed a simplified version of a procedure we reported previously for this task.[18] This employed a script in R using the RDisop library[19] to assign compositions on the basis of m/z limiting to the elements carbon, hydrogen, nitrogen, oxygen, and sodium, and to a 10 ppm error. We discarded assignments falling outside the following elemental composition rules as implausible:[20] H/C min=0.1 max=6, N/C min=0 max=4, O/C min=0 max=3, & C min=2. All formulae are fitted to the m/z value and assumed to represent $[M+H]^+$ ions. While other adducts are possible under ESI conditions, this assumption allowed tentative assignment of possible formulae (and these are overwhelmingly the most likely in the presence of acid in the mobile phase, although we accept that others are quite possible). The plausible potential formulae with the smallest m/z difference from the observed feature is shown. Peaks where no formula is even tentatively assigned are omitted from these tables.

Since the identification of species is outside the remit of this work (and has no bearing on our conclusions), more in-depth analysis to determine formulae was not pursued.

(Table on next page)

Feature m/z	RT (min)	Formula	*ppm error*
98.0607	8.70	C5H8NO	0.92
101.0716	7.46	C4H9N2O	1.21
104.0710	1.63	C4H10NO2	1.58
115.0869	1.89	C5H11N2O	1.74
139.0255	4.63	C4H3N4O2	0.91
141.1027	2.85	C7H13N2O	0.96
153.0411	9.01	C5H5N4O2	0.83
155.0555	2.85	C3H5N7O	0.09
158.0928	5.15	C6H12N3O2	1.18
166.0981	9.95	C8H12N3O	0.20
168.0409	8.15	C6H6N3O3	0.39
171.0408	2.24	C6H7N2O4	1.03
171.0520	7.80	C5H7N4O3	0.88
171.0769	2.71	C7H11N2O3	0.38
172.0358	3.50	C5H6N3O4	0.07
179.0568	7.95	C7H7N4O2	0.79
180.0660	7.42	C9H10NO3	0.20
183.0768	5.22	C8H11N2O3	1.00
188.0795	9.95	C7H12N2O4	1.06
191.0568	7.83	C8H7N4O2	0.53
195.0882	4.51	C8H11N4O2	0.06
196.0935	8.78	C5H14N3O5	0.70
197.0913	8.90	C7H11N5O2	0.01
203.0667	8.38	C7H11N2O5	0.46
207.0532	8.72	C10H9NO4	0.14
207.0619	8.46	C6H11N2O6	0.80
218.0666	8.56	C8H12NO6	0.76
232.0695	9.95	C7H6N9O	0.19
233.0774	8.12	C8H13N2O6	0.07
236.0869	7.68	C5H12N6O5	0.01
237.0710	8.46	C5H11N5O6	0.25
239.0765	6.30	C7H9N7O3	0.75
240.0718	4.03	C6H8N8O3	0.55
248.0784	8.03	C10H10N5O3	0.04
255.0816	9.17	C5H13N5O7	0.29
256.1033	5.04	C8H18NO8	0.39
259.0931	8.71	C10H15N2O6	0.24
263.0993	9.45	C8H15N4O6	0.36
264.0972	8.59	C10H12N6O3	0.36
266.1116	8.89	C9H18N2O7	0.57
269.0973	8.89	C6H15N5O7	0.73
282.0951	7.91	C9H12N7O4	0.17
289.1109	9.17	C4H11N13O3	0.49
299.1091	1.63	C9H19N2O9	0.04
307.1378	8.70	C10H15N10O2	0.53
320.1148	8.48	C17H14N5O2	0.09
320.1191	8.62	C7H10N15O	0.47
416.2566	11.11	C9H26N19O	0.40
503.3061	11.59	C35H39N2O	0.34
547.3313	11.80	C35H41N5O	0.27

Figure S41. *Table of selected features from the SD-mix experiment with different minerals, with suggested molecular formulae based on m/z and standard constraints. Only the first 50 features with the closest fit (lowest ppm error) are shown here of several hundred picked features, chosen on the basis of the highest contributions to PC1 in PCA analysis (see section 2.2.2). Features are ordered by m/z.*

3.4 Environment-Directed Complex Mixture Experiments: Functional Examination

3.4.1 Recognition assay using ThT

Procedure:

Following the same procedure as described in Section 2.3.2, with the modification that slightly smaller amounts of all materials were used (maintaining the same ratio: 25 µl 'sample' & 10 µl ThT 'working solution'), and each sample tested in triplicate.

3.4.2 Inspection of Assembly/Aggregation using TEM

Procedure:

Following the same procedure described in Section 2.3.3, solutions of the product ensembles produced in Section 3.2 were inspected using TEM microscopy. Results are shown in Figures S41 & S42, and discussed below.

Observations:

- **Observable morphological difference between populations:** While in most samples more than one structure is present, observation of many images of the different populations at low magnification (Figure S41) shows that some populations are clearly distinct to others. (e.g. those produced in the presence of Goethite and Natrolite are clearly distinct). Observation at higher magnifications (Figure S42) reveals different structural detail in many samples, consistent with qualitative difference in the material present.
- **Morphological observations not directly correlated with recognition assay results:** Where populations have similar results in ThT recognition assay, they do not necessarily appear to produce morphologically similar assemblies when observed by TEM (e.g. those produced in the presence of Goethite and Natrolite are clearly distinct). This suggests that variation in recognition and assembly properties are not mediated by the same, simple, factor (e.g. amount of material present), but from qualitative differences between the products present.

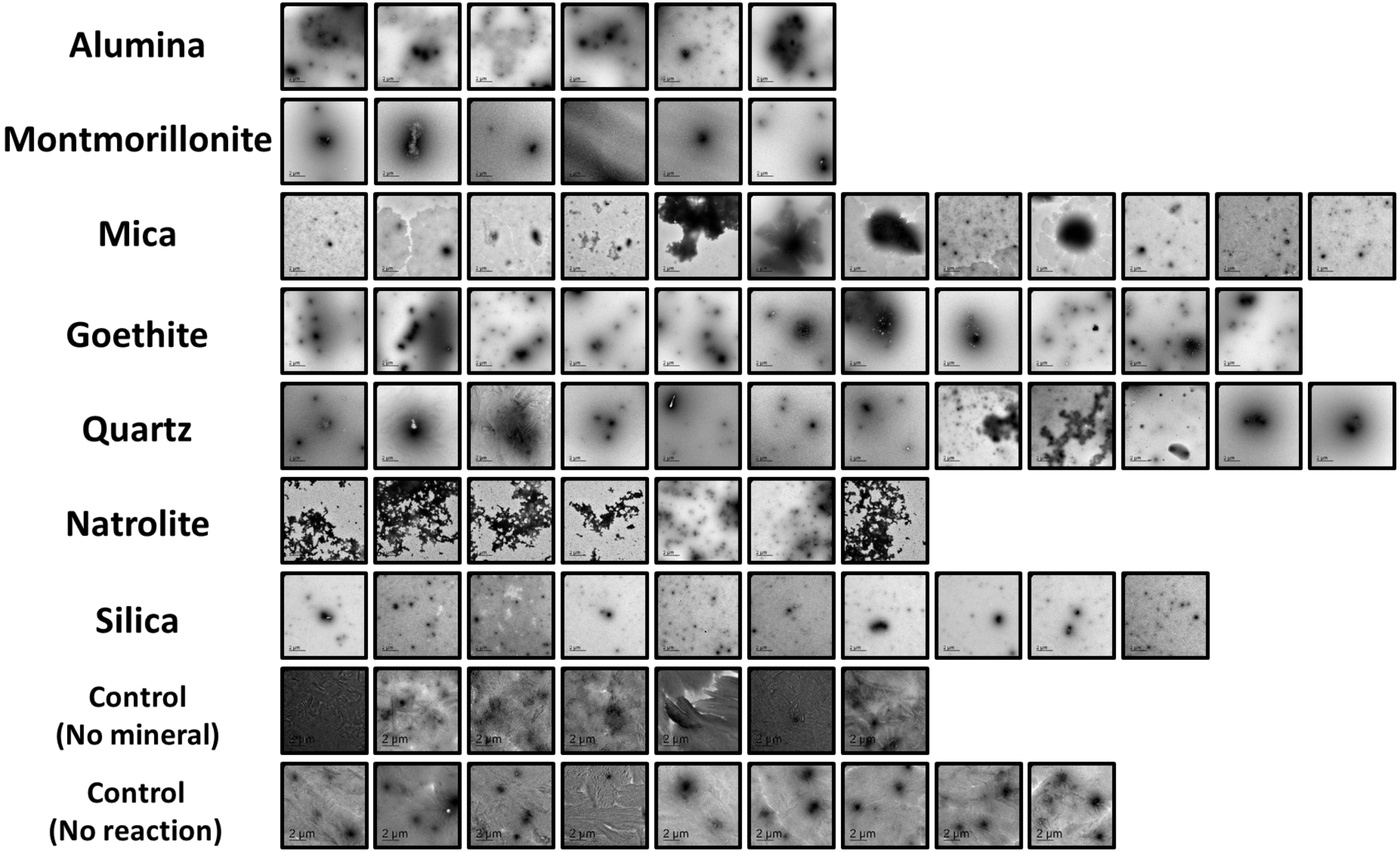

Figure S42. *TEM images showing assembly/aggregation of product ensembles from reaction of SD Mix in the presence of different minerals, at low magnification (scale bar = 2 µm).*

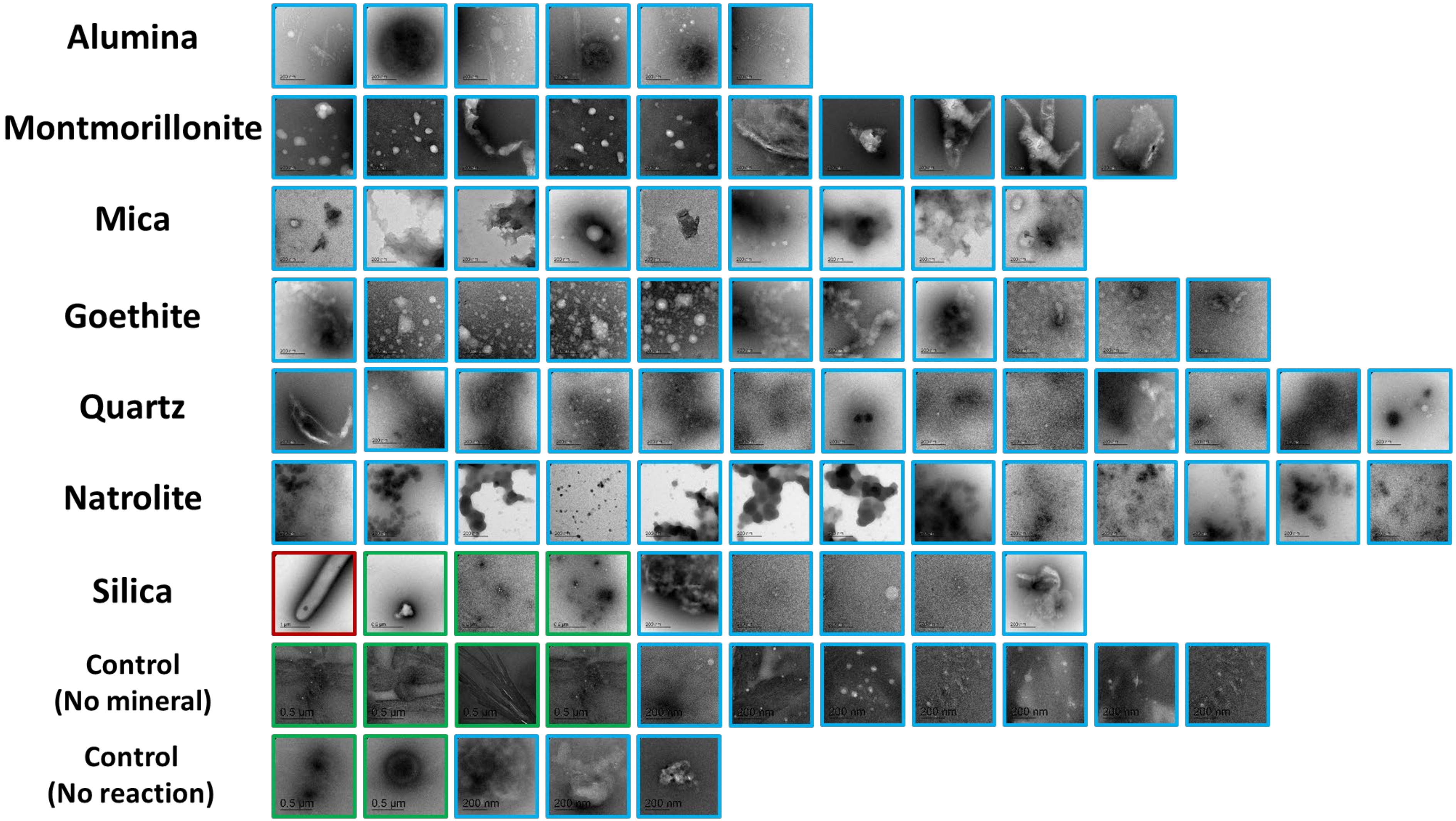

Figure S43. *TEM images showing assembly/aggregation of product ensembles from reaction in the presence of different minerals, at low magnification (where outline is blue, scale bar = 0.2 µm; where outline is green, scale bar = 0.5 µm; where outline is red, scale bar = 1.0 µm).*

4 References

1. R: A language and environment for statistical computing. (R Foundation for Statistical Computing, Vienna, Austria., 2008). http://www.r-project.org
2. Chambers, M. C. *et al.* A cross-platform toolkit for mass spectrometry and proteomics. *Nat. Biotechnol.*, **30**, 918-920 (2012).
3. Smith, C. A., Want, E. J., O'Maille, G., Abagyan, R. & Siuzdak, G. XCMS: Processing mass spectrometry data for metabolite profiling using Nonlinear peak alignment, matching, and identification. *Anal. Chem.*, **78**, 779-787 (2006).
4. Long, W. in *Application Note 5991-5571EN.* (Agilent Technologies, Inc.). http://www.agilent.com/cs/library/applications/5991-5571EN.pdf
5. Commeyras, A. *et al.* Prebiotic synthesis of sequential peptides on the Hadean beach by a molecular engine working with nitrogen oxides as energy sources. *Polym. Int.*, **51**, 661-665 (2002).
6. Dick, J. M., LaRowe, D. E. & Helgeson, H. C. Temperature, pressure, and electrochemical constraints on protein speciation: Group additivity calculation of the standard molal thermodynamic properties of ionized unfolded proteins. *Biogeosciences*, **3**, 311-336 (2006).
7. Dick, J. M. Calculation of the relative metastabilities of proteins using the CHNOSZ software package. *Geochem. Trans.*, **9** (2008).
8. Anderson, G. M. *Thermodynamics of natural systems*. (Cambridge University Press, 2009).
9. James, G., Witten, D., Hastie, T. & Tibshirani, R. *An introduction to statistical learning: with applications in R*. Vol. 103 (Springer Science & Business Media, 2013).
10. Glansdorff, P. & Prigogine, I. Structure, stability and fluctuations. *New York, NY: Interscience* (1971).
11. Le, S., Josse, J. & Husson, F. FactoMineR: An R package for multivariate analysis. *J Stat Softw*, **25**, 1-18 (2008).
12. rgl: 3D Visualization Using OpenGL (2016). https://r-forge.r-project.org/projects/rgl/
13. Origin Pro 2016 (OriginLab, Northampton, MA, 2016). http://www.originlab.com
14. Venables, W. N., Ripley, B. D. & Venables, W. N. *Modern applied statistics with S*. 4th edn, (Springer, 2002).
15. Kyle, S., Felton, S. H., McPherson, M. J., Aggeli, A. & Ingham, E. Rational molecular design of complementary self-assembling peptide hydrogels. *Adv Healthc Mater*, **1**, 640-645 (2012).
16. Schwartz, A. W. Intractable mixtures and the origin of life. *Chem. Biodiversity*, **4**, 656-664 (2007).
17. Miller, S. L. A Production of amino acids under possible primitive earth conditions. *Science*, **117**, 528-529 (1953).
18. Cooper, G. J. T., Surman, A. J., McIver, J., Colon-Santos, S., Gromski, P. S., Buchwald, S., Suarez Marina, I., Cronin, L. Miller–Urey Spark-Discharge Experiments in the Deuterium World. *Angew. Chem. Int. Ed.*, **56**, 8079-8082 (2017).
19. Böcker, S., Lipták, Z., Martin, M., Pervukhin, A., Sudek, H. Decomp—from interpreting Mass Spectrometry peaks to solving the Money Changing Problem. *Bioinformatics*, **24**, 591–593 (2008).
20. Kind, T., Fiehn, O. Seven Golden Rules for heuristic filtering of molecular formulas obtained by accurate mass spectrometry. *BMC Bioinformatics*, **8**,105-125 (2007)
